# Supplementary material for: Healthcare professionals’ perspectives of the provision of, and challenges for, eating, drinking and psychological support post stroke: findings from semistructured interviews across India
Source: BMJ Open. 2023 Oct 26;13(10):e069150. doi: 10.1136/bmjopen-2022-069150 (PMC10603406; doi:10.1136/bmjopen-2022-069150)
Supplement: Supplementary data [file bmjopen-2022-069150supp001.pdf]

Supplementary File 1: WP2 Interview Guide

Q1. Can you tell me about your role?

Q2. Can you tell me about how your service currently manages patients when they have been discharged from hospital?

Q3. What options do patients have if they need support for their stroke after they have been discharged from hospital?

Q4. Are there any services available to support patients or their relatives in relation to their eating and drinking after they have been discharged from hospital?

Q5. Are there any services available to support patients or their relatives in relation to their psychological adjustment to the stroke after they have been discharged from hospital?

Q6. What works well about support that is available for patients in the community? Are there any aspects that do not work as well?

Q7. What role do the patient's family/relatives have in caring for patients when they have been discharged from hospital?

Q8. Do the patient's family/relatives receive any information about how to provide care for their relative who has had a stroke?

Q9. Do the patient's family/relatives receive any information about how to provide care for their relative who has had a stroke?

Q10. What works well about involving the patient's family/relatives once they are in the community?

Q11. Are there any aspects that do not work well?

Q12. Is there anything else that you would like to add?

Thank you for taking the time to take part in this interview.

Jones SP, *et al. BMJ Open* 2023; 13:e069150. doi: 10.1136/bmjopen-2022-069150
