# Supplementary material for: Healthcare professionals’ perspectives of the provision of, and challenges for, eating, drinking and psychological support post stroke: findings from semistructured interviews across India
Source: BMJ Open. 2023 Oct 26;13(10):e069150. doi: 10.1136/bmjopen-2022-069150 (PMC10603406; doi:10.1136/bmjopen-2022-069150)
Supplement: Supplementary data [file bmjopen-2022-069150supp002.pdf]

Study title: **The National Institute for Health Research Global Health Research Group on improving stroke care in India at The University of Central Lancashire - Advancing the INSTRuCT Operations and Network.**

Short title: ***IMPROVIng Stroke care in India - Advancing The INSTRuCT Operations and Network*** **(IMPROVIS-ATION)**

Protocol version: No. 1
Date: 29th June 2020

Contents

i. Study team – Roles & Contact details 4
ii. Study summary 5
iii. Abbreviations 7
1. Background 8
2. Aims of the research 11
Figure 1. Study flow chart 12
2.1 Overall participant timeline 13
Figure 2. Study period timeline 13
3. WP1 Protocol: Hydration and swallowing in hospital 14
3.1 WP1 aim and objectives 14
3.2 WP1 study design 14
3.3 WP1 study setting 14
3.4 WP1 participant eligibility 15
3.5 WP1 consent procedure 15
3.6 WP1 data collection 15
3.7 WP1 outputs 156
4. WP2 Protocol: Post-discharge support 186
4.1 WP2 aims and objectives 186
4.2 WP2 study design & methods 187
4.3 WP2 study setting 197
4.4 WP2 participant eligibility 197
4.5 WP2 participant recruitment 17
4.6 WP2 consent procedures 198
4.7 WP2 data collection 18
4.8 WP2 outputs 18
5. WP3 Protocol: Multi-site ethical approval process 219
5.1 WP3 aim and objectives 219
5.2 WP3 study design 219
5.3 WP3 study setting 20
5.4 WP3 participant eligibility 20
5.5 WP3 participant recruitment 20
5.6 WP3 data collection 20
5.7 WP3 consent procedure 20
5.8 WP3 outputs 20

Protocol version: No. 1
Date: 29th June 2020

6. Overall operations across work packages 24
6.1 Analysis 24
6.2 Data management 24
6.3 Monitoring 25
6.4 Ethics and regulatory approval 25
6.5 Adverse events & safeguarding 263
6.6 Dissemination plan 26
6.7 Study management 26
7. References 288
8. Appendices and topic guides 289

Jones SP, *et al. BMJ Open* 2023; 13:e069150. doi: 10.1136/bmjopen-2022-069150

i. Study team – Roles & Contact details

Name	Role (*Specialism*)	Institution	Contact
Professor Dame Caroline Leigh Watkins	Director	UCLan	CLWatkins@uclan.ac.uk
Denise Forshaw	Deputy Director (*Operations and Governance, Lancashire CTU*)	UCLan	DForshaw@uclan.ac.uk
Professor Catherine Elizabeth Lightbody	Deputy Director (*Nursing; Stroke*)	UCLan	CELightbody@uclan.ac.uk
Dr Pallab Maulik	Deputy Director (*Psychiatry*)	George Institute For Global Health	PMaulik@georgeinstitute.org.in
Professor Jeyaraj Pandian	Deputy Director (*Neurology*)	Christian Medical College	jeyarajpandian@hotmail.com
Dr Liz Boaden	Collaborator (*Stroke*)	UCLan	EBoaden1@uclan.ac.uk
Professor Dominique Cadilhac	Collaborator (*Nursing; Research*)	Monash University, Australia	dominique.cadilhac@monash.edu
Professor Andy Clegg	Collaborator (*Health services*)	UCLan	AClegg3@uclan.ac.uk
Professor Mark Gabbay	Collaborator (*Health services*)	University of Liverpool	M.B.Gabbay@liverpool.ac.uk
Rachel Georgiou	Collaborator (*Stroke*)	UCLan	RGeorgiou@uclan.ac.uk
Dr Jo Gibson	Collaborator (*Stroke*)	UCLan	JGibson4@uclan.ac.uk
Professor Maree Hackett	Collaborator (*Epidemiology*)	UCLan	maree.hackett@sydney.edu.au
Dr Yogeshwar Kalkonde	Collaborator (*Neurology*)	Society for Education, Action and Research in Community Health, India	yvkalkonde@gmail.com
Professor Caroline Sanders	Collaborator (*Patient and Carer engagement*)	The University of Manchester	Caroline.Sanders@manchester.ac.uk
Professor Padma Srivastava	Collaborator (*Stroke*)	All India Institute of Medical Sciences	vasanthapadma123@gmail.com
Professor PN Sylaja	Collaborator (*Stroke*)	Sree Chitra Tirunal Institute for Medical Sciences and Technology	sylajapn@sctimst.ac.in
Kamran Baqai	Research Associate	UCLan	KBaqai1@uclan.ac.uk
Dr Steph Jones	Programme Manager	UCLan	SJones10@uclan.ac.uk
Dr Gordon Prescott	Lead Statistician	UCLan	GPrescott1@uclan.ac.uk
Jo Weldon	Research Associate	UCLan	jcweldon@uclan.ac.uk

Protocol version: No. 1
Date: 29th June 2020

ii. Study summary

**Title**	**IMPROVI**ng **S**troke care in India **- A**dvancing **T**he **I**NSTRUCT **O**perations and **N**etwork (**IMPROVIS-ATION**)
**Design**	Exploratory studies
**Aim**	Work package 1 (WP1) aim:<br>• To explore and gain understanding of how stroke services are set up and operate in order to identify how to implement a hydration and swallowing care bundle.<br><br>Work package 2 (WP2) aims:<br>• To map the current resources available to manage hydration and swallowing problems and psychosocial support following discharge.<br>• Identify the key components of potential interventions for the management of hydration and swallowing, and psychosocial support post-discharge, and explore potential options for interventions with clinical staff, relatives of stroke patients and stroke patients.<br><br>Work package 3 (WP3) aim:<br>• With key stakeholders, to map out the current ethics approval process, identify challenges, enablers and the feasibility of establishing a stream-lined multi-centre ethics approval process for stroke studies within the Indian Stroke Clinical Trial (INSTRuCT) network.
**Population**	WP1 & 2: Convenience sample of stroke patients and/or their relatives and staff from a range of stroke services.<br><br>WP3: Key stakeholders from INSTRuCT network, Forum for Ethics Review Committees in India (FERCI), and the Indian Council of Medical Research (ICMR)
**Research methods**	Qualitative methods – focus groups and interviews.<br><br>WP1: Focus groups and/or interviews- around 10 members of staff, 10 patients and 10 relatives at 5 sites will be included (n=≤150)<br><br>WP2: Focus groups and/or interviews- around 10 members of staff, 10 patients and 10 relatives at 8 sites will be included. Focus groups will be held on two occasions (n=≤480 overall)<br><br>WP3: Focus groups and/or interviews involving around 10 people will take place at convenient locations with around 60 key stakeholders; identified from across 30 hospital sites and 20-30 key stakeholders from organisations such as FERCI and ICMR (n=≤60).
**Outputs**	WP1:<br>• An understanding of current service provision and processes<br>• An understanding of barriers and facilitators to the implementation of a hydration and swallowing care bundle from a range of perspectives<br><br>WP2:<br>• An understanding of current service provision and resources<br>• An understanding of the needs and preferences of patients and their relatives, and clinical staff in relation to the management of hydration and swallowing and psychosocial support following discharge from hospital<br>• Identification of key components of interventions for supporting patients and their relatives following discharge from hospital

	WP3:<br>• An understanding of the current ethics approval processes<br>• Identification of potential challenges, enablers, and feasibility of developing a stream-lined multi-centre ethics approval process for stroke studies within the INSTRuCT network.
**Duration**	1 year (1 August 2020 – 31 July 2021)
**Sponsor**	The University of Central Lancashire
**Funder**	National Institute of Health Research (NIHR)
**Chief Investigator**	Professor Dame Caroline Leigh Watkins
**Collaborating Institutes**	• University of Central Lancashire, UK<br>• University of Manchester, UK<br>• University of Liverpool, UK<br>• Monash University, Australia<br>• Christian Medical College, Ludhiana, India<br>• All India Institute of Medical Sciences, New Delhi, India<br>• Sree Chitra Tirunal Institute of Medical Sciences, Trivandrum, India<br>Research sites<br>• All India Institute of Medical Sciences (AIIMS), Bhopal, India<br>• Baptist Christian Hospital (BCH), Tezpur, India<br>• Indira Gandhi Medical College & Hospital (IGMC), Shimla, India<br>• National Institute of Mental Health and Neurosciences (NIMHANS), Bangalore, India<br>• Zydus Hospital, Ahmedabad, India<br>• Government Hospital Trivandrum, India

Jones SP, *et al. BMJ Open* 2023; 13:e069150. doi: 10.1136/bmjopen-2022-069150

iii. Abbreviations

**CDSCO**	Central Drugs Standard Control Organization
**CI**	Chief Investigator
**FERCI**	Forum for Ethics Research Committees in India
**GCP**	Good Clinical Practice
**HMSC**	Health Ministry Screening Committee
**ICMR**	Indian Council of Medical Research
**IMPROVISE**	IMPROVIng StrokE care in India
**IMPROVIS-ATION**	IMPROVIng Stroke care in India - Advancing The INSTRuCT Operations and Network
**INSTRuCT**	Indian Stroke Clinical Trial Network
**NIHR**	National Institute for Health Research
**NBM**	Nil By Mouth
**NGT**	Nasogastric tube
**PI**	Principal Investigator
**SAE**	Serious Adverse Event
**SLT**	Speech and Language Therapist
**STKG**	Stakeholder Group
**WP**	Work Package

Jones SP, *et al. BMJ Open* 2023; 13:e069150. doi: 10.1136/bmjopen-2022-069150

1. Background

Stroke is a recognized global health challenge. Cardiovascular disease including stroke is now the most common cause of death globally (World Health Organisation, 2014). India is one of the three countries with the greatest number of recorded stroke deaths worldwide (Mackay and Mensah, 2004). People in India typically suffer a first stroke at a much younger age than in the UK (India, age 57 (Pandian and Sudhan, 2013); UK, age 72 (male) and age 78 (female) (The Stroke Association 2018), leading to significant socio-economic burden (Dalal, 2006).

In the last 20 years, the UK, along with other higher income countries, has achieved significant improvements in stroke outcomes, including reduced mortality and morbidity. Much of this improvement has been through the introduction of organised stroke unit care. Stroke unit care has great potential to reduce mortality and morbidity worldwide, as it is the most widely applicable and effective intervention (Stroke Unit Trialist Collaboration, 2013).

One element of stroke unit care that is thought to be effective is an organised specialist multidisciplinary team approach and patient-centered care. It has been demonstrated that better adherence to several processes of care in acute stroke is associated with reduced mortality. A recent audit (Bray et al., 2013) showed that the performance of a swallow assessment, and provision of adequate fluid and nutrition, were all associated with lower 30-day mortality. Adherence to these and other care quality indicators may be amenable to improvement via structured intervention.

One way of delivering structured interventions is via care bundles. Care bundles aim to improve standards of care and patient outcomes by promoting the consistent implementation of a group of effective interventions, which when performed together, may have a better outcome than if performed individually (Cadilhac et al., 2017). They can be used to ensure the delivery of the minimum standard of care. One of the key elements of acute stroke care which are suitable for implementation via care bundles are hydration and swallowing.

In this study we will build on the National Institute for Health Research Global Health Research Group funded project Improving Stroke Care in India (IMPROVISE), in which a Care Bundle was

implemented to prevent dehydration in hospital, and to improve staff competence to assess dysphagia and manage oral intake. This reduced the need for patients screening positive for dysphagia to stay nil by mouth (NBM) to avoid aspiration pneumonia. These elements rely heavily on having appropriately educated and skilled nurses, working within a multidisciplinary team, to perform them effectively. IMPROVISE has introduced training and care pathways to allow staff to follow the initial dysphagia screen with comprehensive assessment to inform in-hospital management of oral intake. In this study we would like to explore and gain an understanding of how stroke services are set up and operate in order to identify how to implement a hydration and swallowing care bundle through interviews and discussions across 5 hospitals.

During exploration of how best to support patients and families to engage in in-hospital care, the issue of psychosocial support post-discharge has been raised; there is little available. Our co-applicants' ATTEND Trial also found that the trial Rehabilitation Therapists were hampered in their efforts to provide rehabilitation support because patients and families wanted instead to focus on psychosocial issues (The ATTEND Collaborative Group, 2017). Although there is a paucity of data on the long-term consequences of stroke on families in India, anecdotal evidence indicates a significant burden, particularly in rural areas, and in less educated families (Dalal, 2006).

In India, most people with stroke have recovery supported by families and their psychosocial problems could differ significantly from those experienced by families in developed countries. In a study of 152 carers (mean age 40·5), 85% had carer strain, with depression in 43% and anxiety in 47% (Raju et al., 2012). Organised support for patients and families is rare but crucial.

In IMPROVIS-ATION we will explore through interviews and discussion the potential for providing post-discharge support psychosocial support as well as hydration and swallowing. This could improve patient complications and reduce psychological distress for both patients and relatives, reducing morbidity and mortality.

During IMPROVISE and through discussions with our India-based collaborators, it was identified that a streamlined ethical approvals process could help to facilitate stroke research studies. Indian research ethics requires individual approval from each site, with Health Ministry Screening Committee (HMSC) approval finally conferred only after all site approvals are gained. Preliminary discussions with members of Indian Stroke Clinical Trial

Network (INSTRuCT) the Forum for Ethics Research Committees in India (FERCI) and the Indian Council for Medical Research (ICMR) suggest that there is the potential for a multi-centre ethics processes which would facilitate stroke and other multi-site clinical research studies in the future.

Jones SP, *et al. BMJ Open* 2023; 13:e069150. doi: 10.1136/bmjopen-2022-069150

2. Aims of the research

Three exploratory work package studies (WPs) will establish the foundations to underpin future planned work to improve the delivery of acute and community stroke services that support stroke survivors.

Work package 1 (WP1) aim:

- To explore and gain an understanding of how stroke services are set up and operate in order to identify how to implement a hydration and swallowing care bundle.

Work package 2 (WP2) aims:

- To map the current resources available for hydration and swallowing problems, and psychosocial support following discharge.
- To identify the key components of potential interventions for the management of hydration and swallowing, and psychosocial support, post-discharge; and, explore potential options for interventions with clinical staff, stroke patients, and their relatives.

Work package 3 (WP3) aim:

- With key stakeholders, to map out the current ethics approval process, identify challenges, enablers and the feasibility of establishing a stream-lined multi-centre ethics approval process for stroke studies within the INSTRuCT network.

Figure 1. Study flow chart

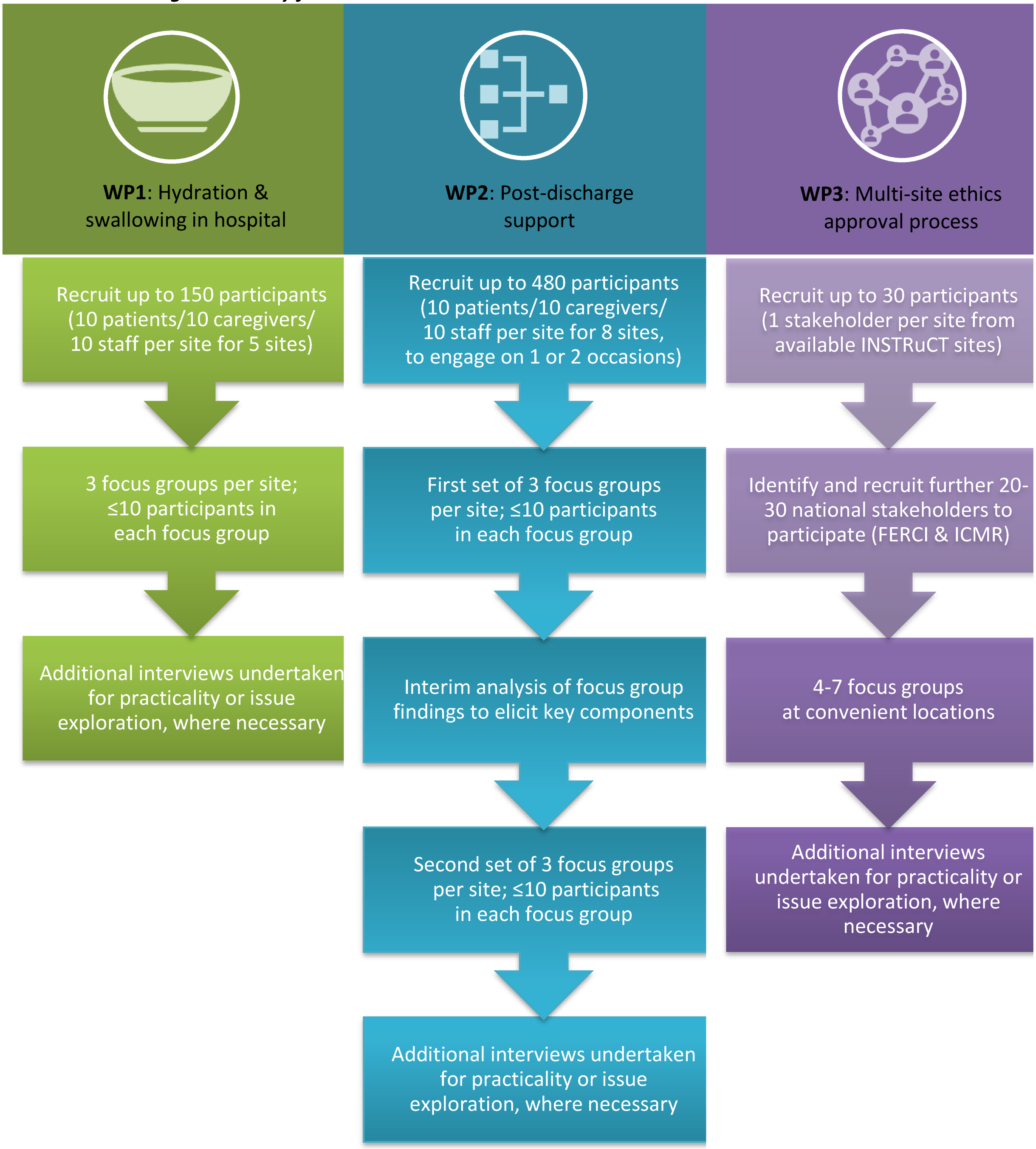

Protocol version: No. 1
Date: 29th June 2020

2.1 Overall participant timeline

Participants will be recruited between 1 November 2020 and 31 March 2021.

Figure 2. Study period timeline

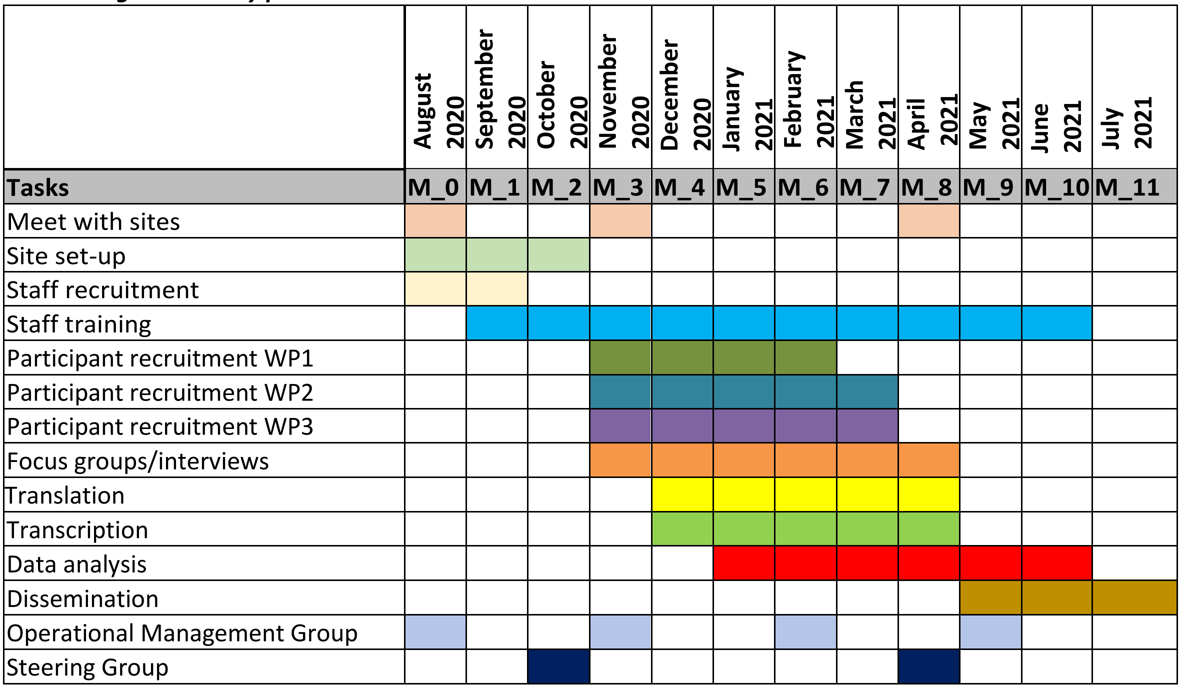

Protocol version: No. 1
Date: 29th June 2020

3. WP1 Protocol: Hydration and swallowing in hospital

3.1 WP1 aim and objectives

Aim: To explore and gain an understanding through interviews and focus groups of how stroke services are set up and operate, in order to identify how to implement a hydration and swallowing care bundle.

Objectives:

- To explore with stroke patients, families and staff, current screening, assessment and management for hydration and swallowing
- To understand challenges and solutions to the implementation of a hydration and swallowing management care bundle in hospital
- To understand regional differences
- To identify the key components for a potential implementation strategy of the hydration and swallowing management care bundle

3.2 WP1 study design

An exploratory study using qualitative research methods. Guided by a facilitator, focus groups will be conducted in local languages (with translators present to support participant engagement), with a note-taker and a secondary facilitator. Focus groups will explore topics including: experiences of access to and resources available for the assessment and management of hydration and swallowing; what people would want in relation to improving hydration and swallowing assessment and management within their locality and how this could be achieved. Focus groups will be conducted face-to-face or online using Zoom or similar technology. Semi-structured interviews will explore the same topics as in the focus groups; interviews may be undertaken to supplement focus group discussions, to follow up on focus group findings in more detail, and/or to ensure inclusivity where participant attendance at the focus groups is not feasible due to disabilities, clinical commitments and other practicalities. Interviews will also be conducted in local languages (with translators present, if required), and may be conducted either face-to-face or by telephone or using Zoom or similar technology (Topic guides Appendix 12 (staff members); Appendix 13 Patients/relatives).

3.3 WP1 study setting

This study will be conducted at five hospital sites across India:

1. All India Institute of Medical Sciences (AIIMS), Bhopal
2. Baptist Christian Hospital (BCH), Tezpur
3. Indira Gandhi Medical College & Hospital (IGMC), Shimla
4. National Institute of Mental Health and Neurosciences (NIMHANS), Bangalore
5. Zydus Hospital, Ahmedabad

3.4 WP1 participant eligibility

We will identify a convenience sample (using non-restrictive criteria) of patients and/or their relatives, and staff, with experience of stroke from a range of stroke services.

Subjects

We will recruit up to 150 participants Each site will host three focus groups, which include ≤10 participants in each staff, or patient and relative focus group (maximum individual commitment of 2-4 hours).

Patients

- Eligible for inclusion in the study are adult patients (aged ≥18 years) with experience of stroke or TIA.
- Excluded patients are those with no legally acceptable representative to consent

Relatives

- Eligible for inclusion in the study are adult (aged ≥18 years) relatives or caregivers for the person who has had the stroke or TIA.

Staff

Clinical staff (medical, nursing and therapy) will also be invited to participate.

3.5 WP1 consent procedure

The study will follow the consent procedure as listed in ICH GCP section 4.8 Informed Consent of Trial Subjects, Indian Council of Medical Research (ICMR), Ethical Guidelines for Biomedical Research on Human Participants and Indian Good Clinical Practices as laid down by Central Drugs Standard Control Organization (CDSCO).

Jones SP, *et al. BMJ Open* 2023; 13:e069150. doi: 10.1136/bmjopen-2022-069150

Each patient/relative (Appendix 1 (WP1) and Appendix 5 (WP2)) and staff member (Appendix 3 (WP1)and Appendix 7 (WP2)) will be given a Participant Information Sheet (PIS) (which describes the study by a site RA or member of the clinical team. Participants agreeing to take part will be asked to provide written informed consent (Patients/relatives: Appendix 2 (WP1; Appendix 6 (WP2); Staff members: (Appendix 4 (WP1) and Appendix 8 (WP2)) before their focus group/interview; and, prior to any data being collected.

A legally acceptable representative may sign the consent form on behalf of an illiterate patient after verbally explaining the study details to them (illiterate patients to provide their thumb impression alongside signature of the legally acceptable representative). If neither a patient nor their legally acceptable representative are literate, an impartial witness (any person completely unrelated to the clinical study) may explain the details of the PIS to the patient and sign the consent form on behalf of the patient (who will provide their thumb impression alongside the signature of the impartial witness).

A copy of the consent form will be given to the participant. The original copy will be kept in the relevant Site File.

3.6 WP1 data collection

Demographic (age; sex; occupation) and stroke detail (type of stroke; severity of stroke [mild/moderate/severe]; experience of swallowing difficulties [current/recent/previous]; psychosocial difficulties (WP2 only); time since stroke; any disability [modified Rankin scale: MRS]) will be collected for all recruited participants from the patient and/or relative (Appendix 9). If this is not possible, this information will be recorded from the patient's medical records by a member of the clinical team.

Details of relationship to patient and age will be collected for recruited relatives in the consent form.

Job role, grade and place of work will be collected for all recruited staff members in the consent form.

3.7 WP1 outputs

- An understanding of current service provision and processes
- An understanding of barriers and facilitators from a range of perspectives

4. WP2 Protocol: Post-discharge support

4.1 WP2 aims and objectives

Aim: Through interviews and focus groups to:

- Map the current resources available for hydration and swallowing problems and psychosocial support following discharge
- Identify the key components of potential interventions for the management of hydration and swallowing, and psychosocial support post-discharge, and explore potential options for interventions with clinical staff, relatives of stroke patients, and stroke patients

Objectives:

- To explore patient pathways post-discharge and understand resources available for care
- To understand patients' and relatives' concerns, contextual challenges, and solutions
- To understand regional differences
- To identify key components of potential interventions

4.2 WP2 study design

An exploratory study using qualitative research methods. Guided by a facilitator, focus groups will be conducted in local languages (with translators present to support participant engagement), with a note-taker and a secondary facilitator. Focus groups will explore topics including: experiences of, access to and resources available for the on-going assessment and management of hydration and swallowing and post-discharge support; what people would want in relation to improving post-discharge support within their locality and how this could be achieved. Focus groups will be conducted face-to-face or online using Zoom or similar technology. Semi-structured interviews will explore the same topics as in the focus groups; interviews may be undertaken to supplement focus group discussions, to follow up on focus group findings in more detail, and/or to ensure inclusivity where participant attendance at the focus groups is not feasible due to disabilities, clinical commitments and other practicalities.

Interviews will also be conducted in local languages (with translators present, if required), and may be conducted either face-to-face or by telephone or using Zoom or similar technology. (Topic guides Appendix 14 (staff members); Appendix 15 Patients/relatives).

4.3 WP2 study setting

This study will be conducted at eight hospital sites in India, the five listed above and:

1. All India Institute of Medical Sciences (AIMS), New Delhi.
2. Christian Medical College (CMC), Ludhiana, Punjab.
3. Sree Chitra Tirunal Institute of Medical Sciences (SCTIMS), Trivandrum, Kerala.

Subjects

We will recruitment a maximum of 480 participants across the 8 sites. Each site will host a maximum of six focus groups, which include ≤10 participants (staff, or patient and relative focus group).

4.4 WP2 participant eligibility

We will identify a convenience sample (using non-restrictive criteria) of patients and/or their relatives, and staff, with experience of stroke from a range of stroke services.

Patients

- Eligible for inclusion in the study are adult patients (aged ≥18 years) discharged from hospital with experience of stroke.
- Excluded patients are those with no legally acceptable representative to consent

Relatives

- Eligible for inclusion in the study are adult (Aged ≥18 years) relatives or caregivers for the person who has had the stroke following discharge home from hospital.

Staff

Clinical staff (medical, nursing and therapy) will also be invited to participate.

4.5 WP2 participant recruitment

At each hospital site, the site Research Associates (RAs) and/or clinical team will invite patients, and any relatives required to support them, to take part in a focus group or interview; where possible these participants will reflect a range of characteristics, as applicable (age/sex/severity of stroke/experience of swallowing and/or psychosocial difficulties).

The site RAs will also invite a purposive sample of staff participants to represent a range of disciplines and grades of staff involved in the management of hydration and swallowing or post-discharge support.

Participants may choose to contribute to either one or two focus groups and/or interviews (maximum individual commitment of 2-4 hours).

4.6 WP2 consent procedure

As described in WP1.

4.7 WP2 data collection

As described in WP1.

4.8 WP2 outputs

- An understanding of current service provision and resources
- An understanding of the needs and preferences of patients and their relatives, and clinical staff, in relation to the management of hydration and swallowing, and psychosocial support following discharge from hospital
- Identification of the key components of interventions for supporting patients and their relatives following discharge from hospital

5. WP3 Protocol: Multi-site ethical approval process

5.1 WP3 aim and objectives

Aim: With key stakeholders, to map out the current ethics approval process, identify challenges, enablers and the feasibility of establishing a stream-lined multi-centre ethics approval process for stroke studies within the Indian Stroke Clinical Trial (INSTRuCT) network.

Objectives:

- To understand challenges
- To identify enhanced processes for ethical approval

5.2 WP3 study design

Design: an exploratory study using qualitative research methods.

Focus groups will be guided by a facilitator, conducted in local languages (with translators present to support participant engagement), with a note-taker and a secondary facilitator (if required). Focus groups will be conducted face-to-face at convenient locations or online using Zoom or similar technology. Focus group discussion topics will include: How the current ethics approval processes currently operates; what are the current challenges; are there any changes that the group like to see made to the ethics approval process and how might these be implemented.

Covering the same topics, semi-structured interviews may be undertaken to inform focus group discussions, to follow up on focus group findings in more detail, and/or to ensure inclusivity where participant attendance at focus groups is not feasible due restrictions on movement, other practicalities and clinical commitments. Interviews will also be conducted in local languages (with translators present to support participant engagement) and may be conducted either face-to-face or by telephone or video-conferencing technology (Stakeholder topic guide Appendix 16).

5.3 WP3 study setting

The INSTRuCT network relevant organisations, members of the Forum for Ethics Review Committees in India (FERCI) and the Indian Council of Medical Research (ICMR).

5.4 WP3 participant eligibility

Key stakeholders will be invited to participate in focus groups and interviews as follows:

- Employed by an appropriate INSTRuCT site, within either stroke or ethics unit
- From relevant organisations, such as the Forum for Ethics Review Committees in India (FERCI) and the Indian Council of Medical Research (ICMR)
- Actively involved in developing multi-centre research ethics processes though IMPROVIS-ATION

5.5 WP3 participant recruitment

We will recruitment a maximum of 60 participants (30 INSTRuCT network site stakeholders (one per site) and up to 30 FERCI and/or ICMR stakeholders). The programme manager will invite site stakeholders to take part, and organisational stakeholders (FERCI/ICMR) will self-nominate themselves to participate after initial approach through the INSTRuCT network.

Between 4-7 focus groups will be conducted at convenient locations, which include ≤10 participants in each focus group (any combination of available stakeholders; maximum individual commitment of 2-4 hours).

The findings from the focus groups and interviews will be synthesised and presented at a final discussion group to identify the key components of any changes and to agree next steps.

5.6 WP3 data collection

Job role, place of work and ethics committee experience information will be collected for all recruited key stakeholders in the consent form.

5.7 WP3 consent procedure

Each identified key stakeholder will be given a PIS describing the study (Appendix 10) by a member of the research team, issued via e-mail. Any key stakeholder agreeing to take part will be asked to provide written informed consent (Appendix 11) prior to their participation in a focus group or interview.

5.8 WP3 outputs

- An understanding of the current ethics approval processes
- Identification of potential challenges, enablers, and feasibility of developing a stream-lined multi-centre ethics approval process for stroke studies within the INSTRuCT network.

6. Overall operations across work packages

6.1 Analysis

Demographic data will be analysed descriptively. Focus groups/Interviews will be transcribed, and then analysed utilising NVivo (a qualitative data analysis computer software package) to support the analysis process. Framework will be used, this permits identification and cross-classification of variables through: identification of key concepts, mapping their range and diversity, interpreting patterns of association and investigating possible reasons. This would give a local service provider perspective on barriers/facilitators to implementing the potential pathways/reforms in the future, ensuring services fit needs. Discussion notes will also be collated and summarised.

6.2 Data management

Data will be treated and stored according to the General Data Protection Regulation (Information Commissioners Office 2018), the Data Protection Act (2018), Caldicott Principles (Caldicott 2013) and the National Institute for Health Research (NIHR) Good Clinical Practice (GCP) Standards.

Written consent forms will be stored in each hospital's Site File (WPs 1 and 2) and at CMC, the Co-ordinating Centre (WP3). All other data will be stored electronically in password-protected files. Members of the research team will have access to project files and data as appropriate to their role.

Participants will be assigned a participant number, this will be used to anonymise their data. Their anonymised data will be typed up into electronic format and stored in a secure OneDrive folder. Audio files from digital voice recorders will be translated, translations checked and transcribed. Once the transcription has been checked the audio file will be deleted. Anonymised transcriptions will be stored on the secure University of Central Lancashire network for five years following completion of the study in-line with the UCLan Data Protection Code of Practice.

Any individuals' names mentioned in the focus group/interview discussions will be removed from transcription. It will not be possible to recognise individuals from information in the completed data analysis or write up of final reports and publications, and any direct quotes used will remain anonymous.

6.3 Monitoring

The Project Co-ordinator from CMC and Research Programme Manager at the University of Central Lancashire will monitor training, recruitment and consent procedures.

Monitoring will facilitate confirmation of site adherence to this protocol and Good Clinical Practice (GCP) Guidelines, relevant local and regional ethical requirements, data accuracy and quality. The study may also be audited by external government or regulatory authorities.

In WPs 1 and 2, each site is responsible for maintaining a Site File containing a delegation log, a training log, and validity of consent log. In WP3, CMC will retain a Site File containing: a delegation log and validity of consent log.

Access to files will be made available by all participating sites for monitoring and audit purposes. On-site monitoring will be undertaken by the University of Central Lancashire in the form of (i) scheduled site visits during the study; or, (ii) triggered site visits in response to concerns relating to any study research processes.

6.4 Ethics and regulatory approval

The work packages, developed under this protocol, will be designed and implemented to comply with Ethical Principles for Medical Research Involving Human Patients (World Medical Association Declaration of Helsinki), Ethical Guidelines for Biomedical Research on Human Participants by Indian Council of Medical Research (ICMR), UK

Ethical and Research Governance approval will be obtained from individual sites' ethics committees and The University of Central Lancashire. Health Ministry Screening Committee (HMSC) approval will be sought from ICMR. If appropriate, the study will be registered on www.ctri.nic.in (Clinical Trials Registry-India (CTRI)) before study (patient-related) activities commence.

The CI is responsible for communicating any protocol amendments to the site PI. The site PI is responsible for ensuring that amendments are implemented, and that all documents replaced by 'new' versions are destroyed immediately. Site staff need to be informed of, and start using, new documentation as soon as local approvals are in place.

The site PI is responsible for reporting any protocol violations and routine reporting

outcomes, reporting to the CI and to their own ethical and governing bodies according to the requirements of the study protocol and written approval of the Ethics Committee.

Consent withdrawal

Participants may withdraw from the study without providing a reason. In these circumstances, the participant will be asked if we can retain and use their data. The PIS emphasises participation is voluntary and that people have the right to withdraw up until they take part in a focus group/discussion, or to the point where their data is anonymised after taking part in an interview and the data becomes fully anonymised once the data have been combined for analysis

6.5 Adverse events & safeguarding

Any adverse events directly resulting from the study will be reported to the site PI (who will inform the Programme Manager in the UK), in accordance with ICH Good Clinical Practices.

Any identified safeguarding concerns will be managed in accordance with the policies and procedures at each individual site.

6.6 Dissemination plan

A comprehensive dissemination plan will be developed with stakeholders, including outputs in clinical and academic peer-reviewed journals, conferences and social media outlets.

Study-generated data will be made available to other researchers for secondary research purposes, as agreed by the partnership and in accordance with open access requirements. This process will be managed and assured by the University of Central Lancashire, in accordance with institutional policy and procedures.

6.7 Study management

The study will be managed in accordance with the governance structure and processes in place for the NIHR Global Health Research Group (GHRG).

Professor Dame Caroline Watkins is the Director of the NIHR GHRG, with support from Deputy Directors: Professor Liz Lightbody, Deputy Director (Nursing Stroke), UCLAN; Professor Jeyaraj Pandian, Deputy Director (Neurology), Christian Medical College; Dr Pallab Maulik, Deputy Director (Psychiatry), The George Institute for Global Health. Members of the group have worked collaboratively to deliver a number of innovative large-scale multi-disciplinary studies in stroke including in the UK and India. Further details of the group are available at uclanglobalhealth.co.uk

The progress of the project will be overseen every three months by the Operational Management Group (OMG), which will ensure effective operational management of the project with reference to agreed work plans, milestones and key deliverables. The OMG consists of the Director, Deputy Directors, Work Package and site leads, and the Programme Manager.

The overall delivery of the programme will be overseen by the Steering Group, which provides oversight, advice and support to the project team. The Steering Group will make recommendations relating to project delivery to the funder and project team, undertake periodic reviews, and is responsible for the content of formal reports to the programme funder (NIHR). The group consists of a Chair, Directors, work package leads, representative of the funder, and other experts independent of the programme team. The Steering Group will meet regularly, approximately every 3-6 months depending on the level of activity in the programme.

7. References

Bray B, Ayis S, Campbell J, Hoffman A, Roughton M, Tyrrell PJ, Wolfe CDA, Rudd AG Associations between the organisation of stroke services, process of care, and mortality in England: prospective cohort study. BMJ. 2013, 346

Cadilhac DA, Andrew NE, Lannin NA, Middleton S, Levi CR, Dewey HM, Grabsch B, Faux S, Hill K, Grimley R, Wong A, Sabet A, Butler E, Bladin CF, Bates TR, Groot P, Castley H, Donnan GA, Anderson CS and Australian Stroke Clinical Registry C. Quality of Acute Care and Long-Term Quality of Life and Survival: The Australian Stroke Clinical Registry. Stroke. 2017;48:1026-1032.

Dalal PM. Burden of stroke: Indian perspective. International Journal of Stroke. 2006; 1:164-6

MacKay J, Mensah G. Atlas of Heart Disease and Stroke. World Health Organisation. 2004.

Pandian JD, Sudhan P. Stroke epidemiology and stroke care services in India. Journal of Stroke. 2013;15(3):128-134.

Raju RS, Kaur P and Pandian JD. Psychosocial problems, quality of life, and caregiver burden among stroke caregivers in India. International Journal of Stroke. 2012;7: 100-101. doi:10.1111/j.1747-4949.

Stroke Unit Trialists' Collaboration. Organised inpatient (stroke unit) care for stroke. Cochrane Database of Systematic Reviews. 2013, Issue 9. Art. No.: CD000197

The ATTEND Collaborative Group. Family-led rehabilitation after stroke in India (ATTEND): A randomised controlled trial. The Lancet, 2017; 390:10094. doi.org/10.1016/S0140-6736(17)31447-2.

The Stroke Association. State of the Nation. Stroke statistics. 2016

World Health Organisation. Global status report on non-communicable diseases. 2014. WHO Press, Switzerland.
