# Supplementary material for: Healthcare professionals’ perspectives of the provision of, and challenges for, eating, drinking and psychological support post stroke: findings from semistructured interviews across India
Source: BMJ Open. 2023 Oct 26;13(10):e069150. doi: 10.1136/bmjopen-2022-069150 (PMC10603406; doi:10.1136/bmjopen-2022-069150)
Supplement: Author's manuscript [file bmjopen-2022-069150.draft_revisions.pdf]

BMJ Open is committed to open peer review. As part of this commitment we make the peer review history of every article we publish publicly available.

When an article is published we post the peer reviewers' comments and the authors' responses online. We also post the versions of the paper that were used during peer review. These are the versions that the peer review comments apply to.

The versions of the paper that follow are the versions that were submitted during the peer review process. They are not the versions of record or the final published versions. They should not be cited or distributed as the published version of this manuscript.

BMJ Open is an open access journal and the full, final, typeset and author-corrected version of record of the manuscript is available on our site with no access controls, subscription charges or pay-per-view fees (http://bmjopen.bmj.com).

If you have any questions on BMJ Open's open peer review process please email info.bmjopen@bmj.com

BMJ Open

Healthcare Professionals' perspectives of stroke patient discharge processes and post-discharge support across India.

Journal:	*BMJ Open*
Manuscript ID	bmjopen-2022-069150
Article Type:	Original research
Date Submitted by the Author:	19-Oct-2022

Complete List of Authors:	Jones, Stephanie ; University of Central Lancashire<br>Injety, Ranjit ; Christian Medical College and Hospital Ludhiana<br>Pandian, Jeyaraj ; Christian Medical College and Hospital Ludhiana, Neurology<br>Ratra, Sanjali; Christian Medical College and Hospital Ludhiana<br>Sylaja, PN; Sree Chitra Tirunal Institute for Medical Sciences and Technology, Neurology<br>Babu, Veena; Sree Chitra Tirunal Institute for Medical Sciences and Technology<br>Srivastava, Padma; All India Institute of Medical Sciences<br>Sharma, Sakshi; All India Institute of Medical Sciences<br>Sharma, Sudhir; Indira Gandhi Medical College<br>Kaushal, Pawna; Indira Gandhi Medical College<br>Webster, Jemin; Baptist Christian Hospital Tezpur<br>Koirala, Amrit; Baptist Christian Hospital Tezpur<br>Kulkarni, Girish Baburao; National Institute of Mental Health and Neuro Sciences<br>Dixit, Anand; National Institute of Mental Health and Neuro Sciences<br>Sharma, Arvind; Zydus Research Center<br>Prajapati, Jagruti; Zydus Research Center<br>Weldon, Jo; University of Central Lancashire<br>Kuroski, Jennifer A; University of Central Lancashire<br>Watkins, Caroline; University of Central Lancashire College of Health and Wellbeing, Faculty of Health and Wellbeing<br>Lightbody, Catherine; University of Central Lancashire, School of Nursing

Keywords:	Stroke < NEUROLOGY, STROKE MEDICINE, QUALITATIVE RESEARCH, REHABILITATION MEDICINE

SCHOLARONE™
Manuscripts

I, the Submitting Author has the right to grant and does grant on behalf of all authors of the Work (as defined in the below author licence), an exclusive licence and/or a non-exclusive licence for contributions from authors who are: i) UK Crown employees; ii) where BMJ has agreed a CC-BY licence shall apply, and/or iii) in accordance with the terms applicable for US Federal Government officers or employees acting as part of their official duties; on a worldwide, perpetual, irrevocable, royalty-free basis to BMJ Publishing Group Ltd ("BMJ") its licensees and where the relevant Journal is co-owned by BMJ to the co-owners of the Journal, to publish the Work in this journal and any other BMJ products and to exploit all rights, as set out in our licence.

The Submitting Author accepts and understands that any supply made under these terms is made by BMJ to the Submitting Author unless you are acting as an employee on behalf of your employer or a postgraduate student of an affiliated institution which is paying any applicable article publishing charge ("APC") for Open Access articles. Where the Submitting Author wishes to make the Work available on an Open Access basis (and intends to pay the relevant APC), the terms of reuse of such Open Access shall be governed by a Creative Commons licence – details of these licences and which Creative Commons licence will apply to this Work are set out in our licence referred to above.

Other than as permitted in any relevant BMJ Author's Self Archiving Policies, I confirm this Work has not been accepted for publication elsewhere, is not being considered for publication elsewhere and does not duplicate material already published. I confirm all authors consent to publication of this Work and authorise the granting of this licence.

For peer review only

Title: Healthcare Professionals' perspectives of stroke patient discharge processes and post-discharge support across India

Authors' names and affiliations

Stephanie P Jones[1], Ranjit J Injety[2], Jeyaraj D Pandian[2], Sanjali Ratra[2], P N Sylaja[3], Veena Babu[3], M V Padma Srivastava[4], Sakshi Sharma[4], Sudhir Sharma[5], Pawna Kaushal[5], Jemin J Webster[6], Amrit Koirala[6], Girish Baburao Kulkarni[7], Anand Dixit[7], Arvind Sharma[8], Jagruti Prajapati[8], Jo Catherine Weldon[1], Jennifer A Kuroski[1], Caroline Leigh Watkins[1], Catherine Elizabeth Lightbody[1] on behalf of the NIHR Global Health Research Group on IMPROVIng Stroke CarE in India (IMPROVISE) Collaboration.

[1]University of Central Lancashire, Preston, Lancashire

[2]Christian Medical College and Hospital, Neurology, Ludhiana, India,

[3]Sree Chitra Tirunal Institute for Medical Sciences and Technology, Neurology, Thiruvananthapuram, India,

[4]All India Institute of Medical Sciences, Neurology, New Delhi, India,

[5]IGMC Shimla, Neurology, Shimla, India,

[6]Baptist Christian Hospital, Department of Medicine, Tezpur, India,

[7]NIMHANS Bangalore, Neurology, Bangalore, India,

[8]Zydus Hospitals And Healthcare Research Pvt. Ltd, Neurology, Ahmedabad, India.

Named contact & address/affiliation
Dr Stephanie Jones
E-mail. Sjones10@uclan.ac.uk
Tel. +44 1772 895107
University of Central Lancashire, Preston, Lancashire, UK

Funder: National Institute for Health Research

Key words: Stroke, Post-discharge, Qualitative, India

Word count: 4092

For peer review only

Abstract

Stroke is one of the most common non-communicable diseases in India, and results in significant disability and mortality. The burden of long-term care for many stroke survivors with disabilities in India, is often the responsibility of family members. This qualitative study explores with health professionals the provision of, and challenges for, post-discharge stroke care.

Methods

Semi-structured interviews were undertaken with a purposive sample of stroke-specific healthcare professionals (HCPs) from seven hospitals across India. Interviews were recorded, translated where necessary, transcribed, and analysed in NVivo using constant comparison and thematic analysis.

Results

Interviews with 66 HCPs (23 nurses (14 staff nurses; 7 senior nurse officers; 1 Intensive Care Unit nurse; 1 palliative care nurse)); 16 doctors (10 neurologists; 6 physicians); 10 physiotherapists; 5 speech and language therapists; 4 occupational therapists; 4 dieticians; 2 psychiatrists; and 2 social workers). resulted in three main themes: Integrated inpatient discharge care planning processes; Post-discharge patient and caregiver role and challenges; and, Patient and caregiver engagement post-discharge.

Conclusion

Discharge planning was integrated and customised, but in some sites resources were limited. Task-shifting compensated for a lack of specialists, but was limited by staff education and training. Caregivers faced challenges in accessing and providing post-discharge care. Post-discharge care was mainly hospital-based, supported by tele-services, especially for rural populations. Further research is needed to understand post-discharge care provision and the needs of stroke survivors and their caregivers.

For peer review only

For peer review only - http://bmjopen.bmj.com/site/about/guidelines.xhtml

Strengths and limitations of this study

- We did not seek to interview HCPs based in the community as services varied between the included sites and were not stroke-specific.
- Due to the diversity of the included hospitals we were unable to make comparisons across services.
- It is acknowledged that one in every three patients with stroke do not access appropriate healthcare due to non-affordability, usage of alternative medicines, and difficulty in conveyance to hospital.
- Participants represented a broad range of stroke services; of which, over a third were nurses of varying seniority.
- Interview methods (telephonic or face-to-face) and interviews in local languages maximised participant engagement, and also enabled adherence to COVID-protocols, and inclusion of participants under COVID restrictions at the point of interview.

For peer review only

For peer review only - http://bmjopen.bmj.com/site/about/guidelines.xhtml

Background

Stroke is a significant global health problem and a major cause of mortality and morbidity in high income countries (HICs), and increasingly in low-middle income countries (LMICs)[1]. Seventy percent of strokes occur in LMICs; the subsequent disease burden is greater than in HICs[2]. Life expectancy in India has recently increased to over 60 years of age[3,4] leading to an increase in age-related, non-communicable diseases including stroke[5,6]. In India, the incidence of stroke ranges between 105 and 152/100,000 people per year[7]. Increasing incidence of stroke is observed among younger groups, with first ever strokes occurring around the age of 57 years [8–10], increasing families' burden.

Stroke survivors often experience many disabilities including hemiparesis, cognitive and communication problems, incontinence, dysphagia, and psychological trauma[11], having serious economic and social impacts[12]. In India, the majority of stroke survivors have disabilities, and the burden of long-term care often falls on family members [9]. This is particularly challenging when stroke survivors leave hospital with complex needs; requiring nasogastric tubes, catheters, and support with communication and psychological problems.

Stroke rehabilitation has been found to reduce mortality, decrease institutionalised care[13], and increase functional outcomes[14,15]. Despite the known benefits, India has limited resources to provide post-discharge stroke care[16]. Accessing rehabilitation services is a challenge for many stroke survivors in India. There are enduring barriers including long delays in accessing rehabilitation services, poor transport between home and hospital[17,18], and a lack of trained personnel[9]. There is a lack of data about post-discharge stroke services and the support available for patients and caregivers[14].

This study aimed to explore healthcare professionals' (HCPs') views of post-discharge stroke care provision including preparing stroke patients and their caregivers for hospital discharge and the availability of post-discharge support.

This qualitative study explores with HCPs the provision of, and challenges relating to, post-discharge stroke care.

Design

Qualitative semi-structured interviews.

For peer review only - http://bmjopen.bmj.com/site/about/guidelines.xhtml

For peer review only

Setting and subjects

Interviews were conducted with a purposive sample of HCPs (doctors, nurses, physiotherapists [PTs], occupational therapists [OTs], speech and language therapists [SLTs], dieticians, psychiatrists and social workers [SWs]) at seven hospitals across India (All India Institute of Medical Sciences, New Delhi; Baptist Christian Hospital, Tezpur; Christian Medical College, Ludhiana; Indira Gandhi Medical College & Hospital, Shimla; National Institute of Mental Health and Neurosciences, Bangalore; Sree Chitra Tirunal Institute of Medical Sciences, Trivandrum; Zydus Hospital, Ahmedabad) between July and December 2021.

Methods

Interviews explored HCPs' views of stroke patient management in preparation for discharge and post-discharge support for patients and caregivers, focussing on eating, drinking and psychological support.

The interview guide (Supplementary File 1) was informed by published research and developed collaboratively by the research team. While not formally piloted, the interview guide was revised following feedback from Research Associates (RAs) and Principal Investigators (PIs) at the included hospitals, to ensure that questions could be translated into regional languages (Assamese, Gujarati, Hindi and Malayalam). According to participant preference, interviews were conducted in English or regional languages, and took place either face-to-face or by telephone (dependent on current COVID-19 restrictions).

Data collection

All interviews were digitally recorded, translated (as required) and transcribed verbatim. All transcriptions were verified by RAs prior to analysis. Data collection continued until saturation.

Analysis

Each transcript was read in full, and data explored using open coding, using an inductive approach. Thirty interviews were coded as a group and 36 by individual project members. Confirmative and contradictory results were discussed by the project team for consensus. Data were analysed thematically using NVivo software (version 1.5.2.). This manuscript was prepared in accordance with the Consolidated Criteria for Reporting Qualitative Research (COREQ) guidelines[20] (Supplementary File 2).

For peer review only - http://bmjopen.bmj.com/site/about/guidelines.xhtml

For peer review only

Reflexivity

All interviews were undertaken by RAs (AD, AK, JP, PK, SR, SS, VB) who were known by participants, but were not someone who they worked with clinically and lasted between 15 and 47 minutes. Interviews were conducted in Assamese (n=1), Gujarati (n=1), Hindi (n=12), Malayalam, (n=3) and English (n=51). All members of the project team were involved in data analysis. Dependability of the data may vary based on any staffing changes following the research undertaken, and all interviews took place during the COVID-19 pandemic. Interviews were undertaken with participants from seven hospitals across geographically diverse areas. To support the analysis, a supplementary mapping exercise was undertaken to identify hospital and community-based services. The findings from the mapping activity enabled cross-validation of the interview findings.

Ethical considerations

Ethics approval (REF: 2020-9715) was granted by the Health Ministry's Screening Committee (HMSC), India and each hospital's ethics committee. The study conforms to ethical standards of the Indian Council of Medical Research Guidelines[19]. In the UK, ethical approval was granted by the Science, Technology, Engineering and Medicine Ethics Committee, University of Central Lancashire (STEM 939). Informed consent was obtained from all participants prior to interview.

Results

Sixty-six HCPs participated: 23 nurses (14 staff nurses; 7 senior nurse officers; 1 ICU nurse; 1 palliative care nurse)); 16 doctors (10 neurologists; 6 physicians); 10 PTs; 5 SLTs; 4 OTs; 4 dieticians; 2 psychiatrists; and 2 SWs. Three themes were identified:

1. Integrated Inpatient Discharge Care Planning Processes (5 sub-themes):
    - 1.1. Healthcare System and Resources
    - 1.2. Staff Identification, and Expectations of Caregivers
    - 1.3. Task-shifting
    - 1.4. Patient and Caregiver Training
    - 1.5. Education and Support in Preparation for Discharge
2. Post-Discharge Patient and Caregiver Roles and Challenges (3 sub-themes):
    - 2.1. Patient and Caregiver Characteristics
    - 2.2. Caregiver Roles
    - 2.3. Caregiver Challenges
3. Patient and Caregiver Engagement post-discharge (2 sub-themes):
    - 3.1. Post-Discharge Support - Hospital

For peer review only - http://bmjopen.bmj.com/site/about/guidelines.xhtml

3.2. Post-Discharge Support - Community-based

Theme 1: Integrated Discharge Care Planning Processes

Discharge planning processes were integrated throughout the patient pathway, beginning soon after admission although varied across sites. Discharge planning was frequently dependent on the availability of specialist staff, resources and facilities, and often resulted in task-shifting due to a lack of staff availability/expertise. Participants reported that HCPs provided patients and caregivers with tailored, targeted education, training, and support throughout hospitalisation.

Sub-theme 1.1. Healthcare System and Resources

All sites had localised, inpatient discharge planning processes or protocols in place, but this was largely undocumented.

"We don't have any written protocol but we are observing the patient from the admission." (01-05)

Staff identified that existing post-discharge management systems could be improved by formal documentation to facilitate a more consistent and comprehensive approach.

"I really feel like there is dire need of formulating a kind of discharge protocol for the patient." (03-02)

Sub-theme 1.2. Staff Identification, and Expectations of Caregivers

Identified by the clinical team, caregivers would most often be the spouse, another immediate or extended family member.

"We will ask about the immediate relatives, either a wife or husband, brother or sister, son or daughter. We also identify the immediate family or caregiver." (06-04)

Training took place with patients and caregivers expecting that caregivers would primarily support and provide post-discharge care.

"*They need to know how to give physiotherapy, occupational therapy, mental support, how to give food, speech related, so, they need to be a doctor, they need to be therapist, they need to be a nurse… If the caregiver is not up to the mark, the rehabilitation fails.*" (06-01)

On admission, an individualised approach was used to identify the most appropriate caregiver(s) for tailored training and support.

For peer review only - http://bmjopen.bmj.com/site/about/guidelines.xhtml

"It depends on the status of the patient according to the medical and surgical condition of patient… We educate the relatives and patients as well as possible." (07-03)

In addition to, or in the absence of, family caregivers, some patients employed the services of professional caregivers.

"Some patients have the capacity of paying, so they hire the physiotherapists, and they call them at their home…Some patients cannot pay but they need physiotherapy." (04-04)

Sub-theme 1.3. Task-shifting

Whilst some sites had well-resourced stroke teams, there was a shortage of dieticians, SWs and therapists (PTs, OTs, SLTs, psychologists) at other sites, where existing staff, predominantly nurses, provided information and support.

"At the time of discharge being a nurse, as we do not have speech and language therapist, then we [nurses] try to put patients in the upright position and ask them to speak as much as they can." (04-02)

"*We are at such a place where we do not get any psychologists, that thing [psychological problems] we [nurses] have to manage ourselves because we are very close to the patients*." (04-04)

Staff often described task-shifting as integral to their professional roles and responsibilities; however, the majority had not undertaken any formal training and had developed knowledge and skills largely through experiential learning.

"No, we didn't get any specific training for this [providing dietary advice]. Whatever we have studied during our nursing training, learned from doctors, and from the internet, on that basis we provide this information." (04-01)

Sub-theme 1.4. Patient and Caregiver Training

Patient and caregiver training usually began at admission and continued until discharge, with staff encouraging caregivers to observe all aspects of patient care, as required, including the preparation and administration of medication, positioning, changing dressings, feeding, the management of nasogastric (NG) tubes, maintaining hygiene, and rehabilitation exercises.

"We make sure the person who will be taking care of the patient at home is there from the beginning, so that they are very much part of all the processes that happen in the ward: changing dressings, changing position, and feeding and all those things." (02-06)

For peer review only - http://bmjopen.bmj.com/site/about/guidelines.xhtml

Staff felt that patients and caregivers should be prepared both mentally and physically for discharge but shared few examples of any formal psychological support or preparation.

"We tell them, start observing from now, because when you go home, you have to do it by yourself. So, they also get ready mentally and physically. (02-02)

Sub-theme 1.5. Education and Support in Preparation for Discharge

To prepare the patient and caregiver for discharge, training delivered by HCPs was predominantly provided through verbal instruction and explanation.

"When the relatives are in the hospital, we teach them verbally and some of the relatives they themselves write down [instructions]." (02-01)

Training was often supplemented by the provision of information, in a variety of formats including leaflets, videos, written instructions, diet charts, and discharge summaries. Some PTs provided pre-recorded videos of exercises, available through websites such as YouTube and/or recorded patients undertaking exercises.

"But then we do use videos like I said, sometimes pictures of exercises, but it's not uniform for all. (03-05)

Whilst training took place throughout patients' hospitalisation, education and information was largely provided within 24-48 hours of discharge. Leaflets were available in some sites, in most local languages, covering a range of topics to support the caregiver. In other sites discharge summaries and diet charts were the only source of written information and instruction.

"*In our stroke unit, we have made pamphlets regarding safe swallow techniques and how to feed the patient."* (06-02)

Accessible written information in appropriate languages was viewed as a valuable source of information, enabling information to be passed from the patient or primary caregiver in hospital to the main caregiver at home.

"We have pamphlets which are given to the patient depending on the language of their preference… that helps in dispersing information further because the caregiver who is there with the patient in the hospital may not be the same caregiver who is there at home." (01-04)

For peer review only - http://bmjopen.bmj.com/site/about/guidelines.xhtml

2. Post-Discharge Patient and Caregiver Roles and Challenges (3 sub-themes):

The patient's stroke and patients'/caregivers' levels of literacy were viewed as important factors in the subsequent 'successes' of post-discharge rehabilitation. HCPs identified multiple challenges in providing education and training, and specific challenges for the patients and caregivers themselves.

Sub-theme 2.1: Patient and Caregiver Characteristics

Female, family members were often the preferred caregivers for both male and female patients.

"W*e prefer training the female relative because the boys or males they usually go out for work.*" (02-01)

Stroke severity, post-stroke complications, cognitive and communication problems influenced the complexity and challenges of education and training for patients and their caregivers.

"*Some patients having pressure sores, second or third degree, then it will be really difficult to handle the wound, the relatives need more information about how to care for the wound, how to reduce the severity, when to change dressings, and how much time between changing the patient's position.*" (07-02)

Patients' and caregivers' educational attainments were viewed as important in effective training delivery. Patients and caregivers from rural locations often had lower levels of literacy that impacted their ability to understand, and to pay for post-discharge care needed.

"Those who come from village they are not educated and are daily wage workers who are below the poverty-line… they really cannot understand the importance of [rehabilitation] exercises." (02-03)

Sub-theme: 2.2. Caregiver Roles

Participants described post-discharge stroke care as largely the role of home-based caregivers (mainly family members) who had responsibility for a range of tasks, including rehabilitation, the prevention of complications and subsequent stroke, and the provision of psychosocial support. Caregivers supported activities of daily living such as maintaining hygiene, food preparation tailored to the patient's swallowing ability and dietary requirements (e.g. diabetes, low salt, lower calorie); the preparation and administration of medication; rehabilitation (recommended by PT or aimed at improving speech and/or cognition); facilitating follow-up appointments; seeking help in an emergency; and supporting the patient's psychological/emotional needs.

For peer review only - http://bmjopen.bmj.com/site/about/guidelines.xhtml

"They have to do the medication on time. They have to do the proper positioning, proper personal hygiene to the patient, and then they have to maintain the proper diet to the patient, to give nourishment to the patients. The entire role in the hospital, that has to be continued in the home also." (01-06)

Sub-theme 2.3. Caregiver Challenges

Hospital staff provided support and education to an identified caregiver in hospital in preparation for discharge, although these trained caregivers were not always the primary caregiver at home.

"*Sometimes in rural areas women do not come when their husband is admitted in the hospital and the caretaker is different, so all these things happen, and I feel very bad.*" (01-10)

Staff reported that caregivers encountered a multitude of challenges including: financial constraints in healthcare expenses and loss of income; a lack of time to provide care around work commitments; varying levels of literacy, ability to comprehend, and to adhere to instructions; difficulties transporting the patient to hospital appointments and so resorting to nearer sources of help (including from local healthcare centres (with or without trained professionals), informal help (pharmacists), alternative medicine (AUYSH) and unqualified professionals (faith-healers, quacks); and a dearth of community-based services. Challenges were particularly marked among rural populations.

"*One of the negative aspects of patients being in rural areas is that most caregivers go out to work in daytime... They are unable to give enough time for patient care and exercise.*" (02-03)

Theme 3: Patient and Caregiver Engagement post-discharge

Provided at discharge, follow-up appointments were individualised and based on the patient's level of disability, co-morbidities and available post-discharge support. In some sites, dedicated stroke helplines, Stroke Unit contact details, or individual staff members' phone numbers were provided.

Sub-theme 3.1. Post-Discharge Support - Hospital

Staff reported that it was common for patients to have multiple follow-up appointments with their doctor, who was able to provide advice, treatment and referral to other services, as needed. However, some patients had challenges in attending follow-up appointments, mainly due to the distance to the hospital from their home and travel costs.

"The patient comes after one month or if the patient is outside of the area, he may come after two months, three months. That is up to the patient. That is up to their condition. That is up to their circumstances." (01-01)

For peer review only - http://bmjopen.bmj.com/site/about/guidelines.xhtml

For patients who were asked to, and who were able to, attend follow-up, referral to specialist support was made available if needed or if this was part of on-going treatment.

"Once they come to come back for follow-up in the OPD they are sent to physiotherapy OPD for review. (02-03)

Tele-support was variable in availability and purpose. Staff reported that patients and caregivers were more likely to contact the hospital post-discharge when the patient had complex health needs (from their stroke and/or co-morbidities). The most common reasons for seeking help through teleservices were problems with NG tubes (feeding and blockages); advice about preparing food in different consistencies and condition specific diets (low sugar, low salt); questions about medication (dosage, frequency and side-effects); catheter care; monitoring or changes in blood pressure; clarifications about PT-based exercises; and, psychological problems (including anxiety around recurrent stroke and the impact of stroke on life expectancy).

"The patients are getting discharged, we give them our number. So, at this moment, we are trying to attend to them as much as possible with the phone. So, if they have any problem for example, if there is a catheter problem, or there is NG change we try to solve this through the phone." (02-05)

Some hospitals were able to refer patients with ongoing rehabilitation needs to rehabilitation clinics and networks; and where the patient lived some distance from the hospital to local healthcare facilities.

"There are three options. One is the inpatient programme, once they are medically stable. The second is to get admitted under a rehabilitation package. We have 7, 10 and 30-day packages where they stay in hospital and get rehabilitation. The next option is we have guest houses outside [the hospital] and they can stay there at very minimal prices and they then come on an OPD basis to the physiotherapist. The last option is our network of physios visit them at home or we send them videos through WhatsApp." (03-05)

At some hospitals there were also post-discharge outreach facilities (including palliative care and community nurses), but at others there were no such resources.

"We have palliative care nurses who do a lot of home visits, twice weekly, if there is any patient who is bedridden and fully dependent, those patients, we connect them to the palliative care nurses. Palliative care nurses visit them at home and change NG tubes, catheters, dressings and many more of those things." (02-01)

For peer review only - http://bmjopen.bmj.com/site/about/guidelines.xhtml

"We arrange a particular community level therapist, and we have that contact number too, so they can simply contact us regarding the patient's issues." (06-06)

Sub-theme: 3.2. Post-Discharge Support – Community-based

Outside of hospital-based care, post-discharge support services (such as counselling/psychological support and physiotherapy) were not stroke-specific and were considered by staff to deliver little additional benefit. Broader post-discharge support (outside hospital) was reportedly lacking in availability.

"There are some people who get people to massage [by private physiotherapists] and all the therapy techniques that were taught they will not use, they will use their own technique and do oil massage. So, it doesn't strengthen them, a weakness is there." (02-02)

Patients and caregivers, particularly in rural areas were often reported to have accessed alternative medicine, unqualified 'Doctors' or traditional faith healers post-discharge.

"The community will support more after discharge, they usually do so at the time of rituals. Pujari [priest] will advise many things that are advantageous or disadvantageous." (05-01)

Discussion

To our knowledge, this is the first qualitative study to explore post-discharge stroke care with HCPs providing stroke services across India. This study identified a number of strengths including comprehensive individualised treatment planning, task-shifting where there was a lack of specialists, comprehensive patient and caregiver training, and post-discharge care facilitated by hospital, and community-based services, where available. However, there were also some significant challenges.

Inpatient discharge care-planning processes usually began soon after admission but were often not formally documented, a process which may have facilitated a more consistent and comprehensive approach. The provision of tailored and individualised information, education and training for patients and caregivers was often dependant on staff and resource availability, which commonly resulted in task-shifting. Task-shifting describes a situation where a task is transferred to a HCP with a different or lower level of education and training, or to a person specifically trained to perform limited tasks[21]. As not all hospitals in the study had comprehensive MDTs, with reported shortages of dieticians, SWs and therapy staff, in these instances, nurses would often take on the roles of other HCPs despite limited formal training. Whilst task-shifting often enables a more efficient use of available human resources and can constitute a robust strategy to provide stroke care[22] there is currently no documented evidence

For peer review only - http://bmjopen.bmj.com/site/about/guidelines.xhtml

of stroke-specific task-shifting training for non-neurologist HCPs, with HCPs reporting that they largely developed knowledge and skills through informal or experiential learning.

The resources for post-stroke rehabilitation, especially the therapy workforce and infrastructure, are very limited in many LMICs[23]. Beyond urban tertiary centres, rehabilitation services are scarcely available. There are <10 skilled rehabilitation practitioners per 1 million people in many LMICs, which does not allow specialization to treat a particular condition or disorders[24]. In India, it estimated that there are only 41,567 PTs, 3,100 OTs, and 1,700 SLTs, and these are geographically concentrated in urban areas leaving much of the nation without resources[15]. Given the scarcity of resources, the rehabilitation needs of the stroke survivors, especially in LMICs, remain largely unmet resulting in a growing need for cost-effective, multidisciplinary rehabilitation services, particularly post-discharge[25].

Caregivers, often female family members, were responsible for all aspects of care and activities of daily living post-discharge, results echoed in previous research[26]. Their ability to deliver required healthcare and psychosocial support was often dependent on access to services (geographically and financially), and the availability of other family members generating income in their (or the stroke survivors) absence. Congruent with other reports, HCPs identified challenges in training and education, and consequently difficulties for caregivers when stroke survivors had more severe strokes, requiring complex care[27]. Whilst caregivers may have experienced increased demands, and in some cases limited preparedness for their new roles, unlike research from developed countries[28], this was viewed by HCPs to be a necessary responsibility rather than a burden.

As post-discharge care is frequently the responsibility of family members in LMICs[29], the preparation of caregivers was a vital aspect of in-hospital discharge planning. Training was usually delivered verbally, sometimes supported by alternative formats provided in local languages to enhance accessibility, comprehension and adherence. However, patient and caregiver characteristics (demographic, socio-economic, stroke severity) and social roles (employment status, family structure and dynamics), all had an impact on the subsequent accessibility, availability and provision of post-discharge care. Caregiver literacy was viewed as an importance aspect of successful post-discharge care. Previous research has demonstrated that low functional literacy may contribute to poor health status through a reduced ability to use written instructions and advice, and reduced adherence to medication and rehabilitation regimens[30]. Low literacy levels were viewed as a particular challenge in rural areas.

Post-discharge support provided by hospitals included multiple follow-up appointments, the frequency and number determined by the patient's condition. Post-discharge care included further service referrals (to rehabilitation clinics, local healthcare centres or post-discharge outreach facilities where available).

For peer review only - http://bmjopen.bmj.com/site/about/guidelines.xhtml

However, post-discharge support beyond hospital settings was lacking in the community, as a result alternative medicine, unlicensed practitioners and faith-healers were often sought, particularly among rural populations. In addition, other non stroke-specific rehabilitation services with limited patient benefit were relied upon. Complementary and alternative medicine (CAM) approaches for post-stroke recovery are frequently accessed in India and often include ayurvedic massage, herbal medicine, marma therapy, reiki therapy, homeopathy, intravenous fluids, and opium[31].

Further data and research are needed to understand the support available and needs of stroke survivors and caregivers, to inform the development of innovative post-stroke interventions that are accessible, patient-centered, and culturally sensitive[18].

This study has a number of limitations. Firstly, the study did not seek to interview HCPs based in the community as these services varied between the included sites and were not stroke-specific. Hospital staff broadly lacked awareness of the spectrum of stroke care services available in-hospital (outside of their own profession) and in the community. Due to the diversity of the included hospitals we were unable to make comparisons across services. Finally, it is acknowledged that one in every 3 patients with stroke do not access appropriate healthcare due to non-affordability, usage of alternative medicines, and difficulty in conveyance to hospital[9].

We have also identified several strengths: Interviews were conducted in local languages to maximise engagement. Participants represented a broad range of stroke services; of which, over a third were nurses of varying seniority. By providing participants with a choice of interview method (telephonic or face-to-face), this enabled adherence to COVID-protocols, and supported inclusion of participants under COVID restrictions at the point of interview.

Conclusion

Discharge planning was integrated and customised but in some sites there were limited resources.

Task-shifting among HCPs compensated for a lack of specialists, but limitations were identified in staff education and training. Caregivers faced many challenges in both accessing and providing post-discharge care. Post-discharge care was mainly hospital-based, supported by tele-services, especially for rural populations. Further research is needed to gain an understanding of post-discharge care provision and the needs of stroke survivors and their caregivers.

Funding statement

This research was funded by the National Institute for Health Research (NIHR) [Global Health Research Group on Improving Stroke Care, University of Central Lancashire (16/137/16)] using UK aid from the

For peer review only - http://bmjopen.bmj.com/site/about/guidelines.xhtml

UK Government to support global health research. The views expressed in this publication are those of the author(s) and not necessarily those of the NIHR or the UK government.

Competing interests statement

There are no conflicts of interest to declare.

Data availability statement

No additional data available.

Patient and Public Involvement

No patients involved.

Author contributions

Authors CEL, PMV, PJD, SPN and CLW contributed to the study conception and study design. Data collection was undertaken by PK, SR, SS, AK, AD, JP, VB . Analysis was performed by SJ, RI, JW, WK, PK, SR, SS, AK, AD, JP, CEL, PMV, PJD, SPN, CLW, JW, AS, SS, GK. The first draft of the manuscript was written by SJ, RI and JW. All authors commented on previous versions of the manuscript. All authors read and approved the final manuscript.

For peer review only

For peer review only - http://bmjopen.bmj.com/site/about/guidelines.xhtml

References

1. Feigin VL, Forouzanfar MH, Krishnamurthi R, et al. Global and regional burden of stroke during 1990-2010: findings from the Global Burden of Disease Study 2010. *Lancet*. 2014;383(9913):245-254. doi:10.1016/s0140-6736(13)61953-4

2. Roth GA, Abate D, Abate KH, et al. Global, regional, and national age-sex-specific mortality for 282 causes of death in 195 countries and territories, 1980–2017: a systematic analysis for the Global Burden of Disease Study 2017. *The Lancet*. 2018;392(10159):1736-1788. doi:10.1016/S0140-6736(18)32203-7

3. Yamunadevi A, Sulaja S. Old Age Mortality in India –An Exploration from Life Expectancy at Age 60. *International Journal of Asian Social Science*. 2016;6(12):698-704. doi:10.18488/journal.1/2016.6.12/1.12.698.704

4. Horton R, Das P. Indian health: the path from crisis to progress. *The Lancet*. Published online 2011. doi:10.1016/S0140-6736(10)62179-4

5. Dalal P, Bhattacharjee M, Vairale J, Bhat P. UN millennium development goals: Can we halt the stroke epidemic in India? *Annals of Indian Academy of Neurology*. 2007;10(3):130. doi:10.4103/0972-2327.34791

6. Naik K. Challenges in delivering stroke care in India. *Indian j health sci*. 2016;9(3):245. doi:10.4103/2349-5006.196342

7. Jones SP, Baqai K, Clegg A, et al. Stroke in India: A systematic review of the incidence, prevalence, and case fatality. *International Journal of Stroke*. 2022;17(2):132-140. doi:10.1177/17474930211027834

8. Pandian JD, Jaison A, Deepak SS, et al. Public awareness of warning symptoms, risk factors, and treatment of stroke in northwest India. *Stroke*. 2005;36(3):644-648. doi:10.1161/01.STR.0000154876.08468.a0

9. Pandian JD, Sudhan P. Stroke epidemiology and stroke care services in India. *J Stroke*. 2013;15(3):128-134. doi:10.5853/jos.2013.15.3.128

10. Tripathi M, Padhy UP, Vibha D, et al. Predictors of refractory epilepsy in north India: a case-control study. *Seizure*. 2011;20(10):779-783. doi:10.1016/j.seizure.2011.07.010

11. Lloyd J, Pinto AM, Nair S, Tarey S. A Qualitative Study on Palliative Needs of Stroke Patients in an Indian Tertiary Care Setting - Doctors' Perspective. *Indian J Palliat Care*. 2019;25(1):84-91. doi:10.4103/IJPC.IJPC_161_18

12. Walsh ME, Galvin R, Loughnane C, Macey C, Horgan NF. Community re-integration and long-term need in the first five years after stroke: results from a national survey. *Disability and Rehabilitation*. 2015;37(20):1834-1838. doi:10.3109/09638288.2014.981302

13. Langhorne P, Ramachandra S, Stroke Unit Trialists' Collaboration. Organised inpatient (stroke unit) care for stroke: network meta-analysis. *Cochrane Database Syst Rev*. 2020;4:CD000197. doi:10.1002/14651858.CD000197.pub4

14. Lindley RI, Anderson CS, Billot L, et al. Family-led rehabilitation after stroke in India (ATTEND): a randomised controlled trial. *The Lancet*. 2017;390(10094):588-599. doi:10.1016/S0140-6736(17)31447-2

For peer review only - http://bmjopen.bmj.com/site/about/guidelines.xhtml

15. Pandian JD, Gandhi DBC, Lindley RI, Bettger JP. Informal Caregiving. *Stroke*. 2016;47(12):3057-3062. doi:10.1161/STROKEAHA.116.013701

16. Sylaja PN, Srivastava MVP, Shah S, et al. The SARS-CoV-2/COVID-19 pandemic and challenges in stroke care in India. *Ann N Y Acad Sci*. 2020;1473(1):3-10. doi:10.1111/nyas.14379

17. Banerjee TK, Das SK. Fifty years of stroke researches in India. *Ann Indian Acad Neurol*. 2016;19(1):1-8. doi:10.4103/0972-2327.168631

18. Kamalakannan S, Gudlavalleti Venkata M, Prost A, et al. Rehabilitation Needs of Stroke Survivors After Discharge From Hospital in India. *Arch Phys Med Rehabil*. 2016;97(9):1526-1532.e9. doi:10.1016/j.apmr.2016.02.008

19. Guidelines | Indian Council of Medical Research | Government of India. Indian Council of Medical Research. Published 2017. Accessed June 29, 2022. https://main.icmr.nic.in/content/guidelines-0

20. Tong A, Sainsbury P, & Craig J. Consolidated criteria for reporting qualitative research (COREQ): A 32-item checklist for interviews and focus groups. *International Journal for Quality in Health Care*, 2017;19(6), 349–357. https://doi.org/10.1093/intqhc/ mzm042

21. Joshi R, Alim M, Kengne AP, et al. Task shifting for non-communicable disease management in low and middle income countries--a systematic review. *PLoS One*. 2014;9(8):e103754. doi:10.1371/journal.pone.0103754

22. Lindsay P, Furie KL, Davis SM, Donnan GA, Norrving B. World Stroke Organization global stroke services guidelines and action plan. *International journal of stroke : official journal of the International Stroke Society*. 2014;9 Suppl A100. doi:10.1111/ijs.12371

23. World Health Organization, World Heart Federation, World Stroke Organization. Global atlas on cardiovascular disease prevention and control. *Global atlas on cardiovascular disease prevention and control*. Published online 2011:155-155.

24. World Health Organization. *Global Diffusion of EHealth: Making Universal Health Coverage Achievable: Report of the Third Global Survey on EHealth*. World Health Organization; 2017.

25. Bonita R, Beaglehole R. Stroke prevention in poor countries: time for action. *Stroke*. 2007;38(11):2871-2872. doi:10.1161/STROKEAHA.107.504589

26. Mandowara B, Patel AN, Amin AA, Phatak A, Desai S. Burden Faced by Caregivers of Stroke Patients Who Attend Rural-based Medical Teaching Hospital in Western India. *Ann Indian Acad Neurol*. 2020;23(1):38-43. doi:10.4103/aian.AIAN_406_18

27. Rigby H, Gubitz G, Phillips S. A Systematic Review of Caregiver Burden following Stroke. *International Journal of Stroke*. 2009;4(4):285-292. doi:10.1111/j.1747-4949.2009.00289.x

28. LeLaurin JH, Lamba AH, Eliazar-Macke ND, et al. Postdischarge Intervention for Stroke Caregivers: Protocol for a Randomized Controlled Trial. *JMIR Res Protoc*. 2020;9(11):e21799. doi:10.2196/21799

29. Das SK, Banerjee TK, Biswas A, et al. A Prospective Community-Based Study of Stroke in Kolkata, India. *Stroke*. 2007;38(3):906-910. doi:10.1161/01.STR.0000258111.00319.58

30. Easton P, Entwistle VA, Williams B. Health in the "hidden population" of people with low literacy. A systematic review of the literature. *BMC Public Health*. 2010;10(1):459. doi:10.1186/1471-2458-10-459

For peer review only - http://bmjopen.bmj.com/site/about/guidelines.xhtml

31. Pandian JD, Toor G, Arora R, et al. Complementary and Alternative Medicine Treatments Among Stroke Patients in India. *Topics in Stroke Rehabilitation*. 2012;19(5):384-394. doi:10.1310/tsr1905-384

For peer review only

Supplementary File 1: WP2 Interview Guide

Q1. Can you tell me about your role?

Q2. Can you tell me about how your service currently manages patients when they have been discharged from hospital?

Q3. What options do patients have if they need support for their stroke after they have been discharged from hospital?

Q4. Are there any services available to support patients or their relatives in relation to their eating and drinking after they have been discharged from hospital?

Q5. Are there any services available to support patients or their relatives in relation to their psychological adjustment to the stroke after they have been discharged from hospital?

Q6. What works well about support that is available for patients in the community? Are there any aspects that do not work as well?

Q7. What role do the patient's family/relatives have in caring for patients when they have been discharged from hospital?

Q8. Do the patient's family/relatives receive any information about how to provide care for their relative who has had a stroke?

Q9. Do the patient's family/relatives receive any information about how to provide care for their relative who has had a stroke?

Q10. What works well about involving the patient's family/relatives once they are in the community?

Q11. Are there any aspects that do not work well?

Q12. Is there anything else that you would like to add?

Thank you for taking the time to take part in this interview.

For peer review only - http://bmjopen.bmj.com/site/about/guidelines.xhtml

Study title: **The National Institute for Health Research Global Health Research Group on improving stroke care in India at The University of Central Lancashire - Advancing the INSTRuCT Operations and Network.**

Short title: ***IMPROVIng Stroke care in India - Advancing The INSTRuCT Operations and Network*** **(IMPROVIS-ATION)**

Protocol version: No. 1
Date: 29th June 2020

For peer review only

Contents

i. Study team – Roles & Contact details 4

ii. Study summary 5

iii. Abbreviations 7

1. Background 8

2. Aims of the research 11

Figure 1. Study flow chart 12

2.1 Overall participant timeline 13

Figure 2. Study period timeline 13

3. WP1 Protocol: Hydration and swallowing in hospital 14

3.1 WP1 aim and objectives 14

3.2 WP1 study design 14

3.3 WP1 study setting 14

3.4 WP1 participant eligibility 15

3.5 WP1 consent procedure 15

3.6 WP1 data collection 15

3.7 WP1 outputs 156

4. WP2 Protocol: Post-discharge support 186

4.1 WP2 aims and objectives 186

4.2 WP2 study design & methods 187

4.3 WP2 study setting 197

4.4 WP2 participant eligibility 197

4.5 WP2 participant recruitment 17

4.6 WP2 consent procedures 198

4.7 WP2 data collection 18

4.8 WP2 outputs 18

5. WP3 Protocol: Multi-site ethical approval process 219

5.1 WP3 aim and objectives 219

5.2 WP3 study design 219

5.3 WP3 study setting 20

5.4 WP3 participant eligibility 20

5.5 WP3 participant recruitment 20

5.6 WP3 data collection 20

5.7 WP3 consent procedure 20

5.8 WP3 outputs 20

Protocol version: No. 1
Date: 29th June 2020

For peer review only - http://bmjopen.bmj.com/site/about/guidelines.xhtml

6. Overall operations across work packages 24
6.1 Analysis 24
6.2 Data management 24
6.3 Monitoring 25
6.4 Ethics and regulatory approval 25
6.5 Adverse events & safeguarding 263
6.6 Dissemination plan 26
6.7 Study management 26
7. References 288
8. Appendices and topic guides 289

For peer review only

For peer review only - http://bmjopen.bmj.com/site/about/guidelines.xhtml

i. Study team – Roles & Contact details

Name	Role (*Specialism*)	Institution	Contact
Professor Dame Caroline Leigh Watkins	Director	UCLan	CLWatkins@uclan.ac.uk
Denise Forshaw	Deputy Director (*Operations and Governance, Lancashire CTU*)	UCLan	DForshaw@uclan.ac.uk
Professor Catherine Elizabeth Lightbody	Deputy Director (*Nursing; Stroke*)	UCLan	CELightbody@uclan.ac.uk
Dr Pallab Maulik	Deputy Director (*Psychiatry*)	George Institute For Global Health	PMaulik@georgeinstitute.org.in
Professor Jeyaraj Pandian	Deputy Director (*Neurology*)	Christian Medical College	jeyarajpandian@hotmail.com
Dr Liz Boaden	Collaborator (*Stroke*)	UCLan	EBoaden1@uclan.ac.uk
Professor Dominique Cadilhac	Collaborator (*Nursing; Research*)	Monash University, Australia	dominique.cadilhac@monash.edu
Professor Andy Clegg	Collaborator (*Health services*)	UCLan	AClegg3@uclan.ac.uk
Professor Mark Gabbay	Collaborator (*Health services*)	University of Liverpool	M.B.Gabbay@liverpool.ac.uk
Rachel Georgiou	Collaborator (*Stroke*)	UCLan	RGeorgiou@uclan.ac.uk
Dr Jo Gibson	Collaborator (*Stroke*)	UCLan	JGibson4@uclan.ac.uk
Professor Maree Hackett	Collaborator (*Epidemiology*)	UCLan	maree.hackett@sydney.edu.au
Dr Yogeshwar Kalkonde	Collaborator (*Neurology*)	Society for Education, Action and Research in Community Health, India	yvkalkonde@gmail.com
Professor Caroline Sanders	Collaborator (*Patient and Carer engagement*)	The University of Manchester	Caroline.Sanders@manchester.ac.uk
Professor Padma Srivastava	Collaborator (*Stroke*)	All India Institute of Medical Sciences	vasanthapadma123@gmail.com
Professor PN Sylaja	Collaborator (*Stroke*)	Sree Chitra Tirunal Institute for Medical Sciences and Technology	sylajapn@sctimst.ac.in
Kamran Baqai	Research Associate	UCLan	KBaqai1@uclan.ac.uk
Dr Steph Jones	Programme Manager	UCLan	SJones10@uclan.ac.uk
Dr Gordon Prescott	Lead Statistician	UCLan	GPrescott1@uclan.ac.uk
Jo Weldon	Research Associate	UCLan	jcweldon@uclan.ac.uk

Protocol version: No. 1
Date: 29th June 2020

For peer review only - http://bmjopen.bmj.com/site/about/guidelines.xhtml

ii. Study summary

**Title**	**IMPROVI**ng **S**troke care in India **- A**dvancing **T**he **I**NSTRUCT **O**perations and **N**etwork (**IMPROVIS-ATION**)
**Design**	Exploratory studies
**Aim**	Work package 1 (WP1) aim:<br>• To explore and gain understanding of how stroke services are set up and operate in order to identify how to implement a hydration and swallowing care bundle.<br><br>Work package 2 (WP2) aims:<br>• To map the current resources available to manage hydration and swallowing problems and psychosocial support following discharge.<br>• Identify the key components of potential interventions for the management of hydration and swallowing, and psychosocial support post-discharge, and explore potential options for interventions with clinical staff, relatives of stroke patients and stroke patients.<br><br>Work package 3 (WP3) aim:<br>• With key stakeholders, to map out the current ethics approval process, identify challenges, enablers and the feasibility of establishing a stream-lined multi-centre ethics approval process for stroke studies within the Indian Stroke Clinical Trial (INSTRuCT) network.
**Population**	WP1 & 2: Convenience sample of stroke patients and/or their relatives and staff from a range of stroke services.<br><br>WP3: Key stakeholders from INSTRuCT network, Forum for Ethics Review Committees in India (FERCI), and the Indian Council of Medical Research (ICMR)
**Research methods**	Qualitative methods – focus groups and interviews.<br><br>WP1: Focus groups and/or interviews- around 10 members of staff, 10 patients and 10 relatives at 5 sites will be included (n=≤150)<br><br>WP2: Focus groups and/or interviews- around 10 members of staff, 10 patients and 10 relatives at 8 sites will be included. Focus groups will be held on two occasions (n=≤480 overall)<br><br>WP3: Focus groups and/or interviews involving around 10 people will take place at convenient locations with around 60 key stakeholders; identified from across 30 hospital sites and 20-30 key stakeholders from organisations such as FERCI and ICMR (n=≤60).
**Outputs**	WP1:<br>• An understanding of current service provision and processes<br>• An understanding of barriers and facilitators to the implementation of a hydration and swallowing care bundle from a range of perspectives<br><br>WP2:<br>• An understanding of current service provision and resources<br>• An understanding of the needs and preferences of patients and their relatives, and clinical staff in relation to the management of hydration and swallowing and psychosocial support following discharge from hospital<br>• Identification of key components of interventions for supporting patients and their relatives following discharge from hospital

Protocol version: No. 1
Date: 29th June 2020

For peer review only - http://bmjopen.bmj.com/site/about/guidelines.xhtml

	WP3:<br>• An understanding of the current ethics approval processes<br>• Identification of potential challenges, enablers, and feasibility of developing a stream-lined multi-centre ethics approval process for stroke studies within the INSTRuCT network.
**Duration**	1 year (1 August 2020 – 31 July 2021)
**Sponsor**	The University of Central Lancashire
**Funder**	National Institute of Health Research (NIHR)
**Chief Investigator**	Professor Dame Caroline Leigh Watkins
**Collaborating Institutes**	• University of Central Lancashire, UK<br>• University of Manchester, UK<br>• University of Liverpool, UK<br>• Monash University, Australia<br>• Christian Medical College, Ludhiana, India<br>• All India Institute of Medical Sciences, New Delhi, India<br>• Sree Chitra Tirunal Institute of Medical Sciences, Trivandrum, India<br>Research sites<br>• All India Institute of Medical Sciences (AIIMS), Bhopal, India<br>• Baptist Christian Hospital (BCH), Tezpur, India<br>• Indira Gandhi Medical College & Hospital (IGMC), Shimla, India<br>• National Institute of Mental Health and Neurosciences (NIMHANS), Bangalore, India<br>• Zydus Hospital, Ahmedabad, India<br>• Government Hospital Trivandrum, India

For peer review only

For peer review only - http://bmjopen.bmj.com/site/about/guidelines.xhtml

iii. Abbreviations

**CDSCO**	Central Drugs Standard Control Organization
**CI**	Chief Investigator
**FERCI**	Forum for Ethics Research Committees in India
**GCP**	Good Clinical Practice
**HMSC**	Health Ministry Screening Committee
**ICMR**	Indian Council of Medical Research
**IMPROVISE**	IMPROVIng StrokE care in India
**IMPROVIS-ATION**	IMPROVIng Stroke care in India - Advancing The INSTRuCT Operations and Network
**INSTRuCT**	Indian Stroke Clinical Trial Network
**NIHR**	National Institute for Health Research
**NBM**	Nil By Mouth
**NGT**	Nasogastric tube
**PI**	Principal Investigator
**SAE**	Serious Adverse Event
**SLT**	Speech and Language Therapist
**STKG**	Stakeholder Group
**WP**	Work Package

For peer review only - http://bmjopen.bmj.com/site/about/guidelines.xhtml

1. Background

Stroke is a recognized global health challenge. Cardiovascular disease including stroke is now the most common cause of death globally (World Health Organisation, 2014). India is one of the three countries with the greatest number of recorded stroke deaths worldwide (Mackay and Mensah, 2004). People in India typically suffer a first stroke at a much younger age than in the UK (India, age 57 (Pandian and Sudhan, 2013); UK, age 72 (male) and age 78 (female) (The Stroke Association 2018), leading to significant socio-economic burden (Dalal, 2006).

In the last 20 years, the UK, along with other higher income countries, has achieved significant improvements in stroke outcomes, including reduced mortality and morbidity. Much of this improvement has been through the introduction of organised stroke unit care. Stroke unit care has great potential to reduce mortality and morbidity worldwide, as it is the most widely applicable and effective intervention (Stroke Unit Trialist Collaboration, 2013).

One element of stroke unit care that is thought to be effective is an organised specialist multidisciplinary team approach and patient-centered care. It has been demonstrated that better adherence to several processes of care in acute stroke is associated with reduced mortality. A recent audit (Bray et al., 2013) showed that the performance of a swallow assessment, and provision of adequate fluid and nutrition, were all associated with lower 30-day mortality. Adherence to these and other care quality indicators may be amenable to improvement via structured intervention.

One way of delivering structured interventions is via care bundles. Care bundles aim to improve standards of care and patient outcomes by promoting the consistent implementation of a group of effective interventions, which when performed together, may have a better outcome than if performed individually (Cadilhac et al., 2017). They can be used to ensure the delivery of the minimum standard of care. One of the key elements of acute stroke care which are suitable for implementation via care bundles are hydration and swallowing.

In this study we will build on the National Institute for Health Research Global Health Research Group funded project Improving Stroke Care in India (IMPROVISE), in which a Care Bundle was

For peer review only - http://bmjopen.bmj.com/site/about/guidelines.xhtml

implemented to prevent dehydration in hospital, and to improve staff competence to assess dysphagia and manage oral intake. This reduced the need for patients screening positive for dysphagia to stay nil by mouth (NBM) to avoid aspiration pneumonia. These elements rely heavily on having appropriately educated and skilled nurses, working within a multidisciplinary team, to perform them effectively. IMPROVISE has introduced training and care pathways to allow staff to follow the initial dysphagia screen with comprehensive assessment to inform in-hospital management of oral intake. In this study we would like to explore and gain an understanding of how stroke services are set up and operate in order to identify how to implement a hydration and swallowing care bundle through interviews and discussions across 5 hospitals.

During exploration of how best to support patients and families to engage in in-hospital care, the issue of psychosocial support post-discharge has been raised; there is little available. Our co-applicants' ATTEND Trial also found that the trial Rehabilitation Therapists were hampered in their efforts to provide rehabilitation support because patients and families wanted instead to focus on psychosocial issues (The ATTEND Collaborative Group, 2017). Although there is a paucity of data on the long-term consequences of stroke on families in India, anecdotal evidence indicates a significant burden, particularly in rural areas, and in less educated families (Dalal, 2006).

In India, most people with stroke have recovery supported by families and their psychosocial problems could differ significantly from those experienced by families in developed countries. In a study of 152 carers (mean age 40·5), 85% had carer strain, with depression in 43% and anxiety in 47% (Raju et al., 2012). Organised support for patients and families is rare but crucial.

In IMPROVIS-ATION we will explore through interviews and discussion the potential for providing post-discharge support psychosocial support as well as hydration and swallowing. This could improve patient complications and reduce psychological distress for both patients and relatives, reducing morbidity and mortality.

During IMPROVISE and through discussions with our India-based collaborators, it was identified that a streamlined ethical approvals process could help to facilitate stroke research studies. Indian research ethics requires individual approval from each site, with Health Ministry Screening Committee (HMSC) approval finally conferred only after all site approvals are gained. Preliminary discussions with members of Indian Stroke Clinical Trial

For peer review only - http://bmjopen.bmj.com/site/about/guidelines.xhtml

Network (INSTRuCT) the Forum for Ethics Research Committees in India (FERCI) and the Indian Council for Medical Research (ICMR) suggest that there is the potential for a multi-centre ethics processes which would facilitate stroke and other multi-site clinical research studies in the future.

For peer review only

For peer review only - http://bmjopen.bmj.com/site/about/guidelines.xhtml

2. Aims of the research

Three exploratory work package studies (WPs) will establish the foundations to underpin future planned work to improve the delivery of acute and community stroke services that support stroke survivors.

Work package 1 (WP1) aim:

- To explore and gain an understanding of how stroke services are set up and operate in order to identify how to implement a hydration and swallowing care bundle.

Work package 2 (WP2) aims:

- To map the current resources available for hydration and swallowing problems, and psychosocial support following discharge.
- To identify the key components of potential interventions for the management of hydration and swallowing, and psychosocial support, post-discharge; and, explore potential options for interventions with clinical staff, stroke patients, and their relatives.

Work package 3 (WP3) aim:

- With key stakeholders, to map out the current ethics approval process, identify challenges, enablers and the feasibility of establishing a stream-lined multi-centre ethics approval process for stroke studies within the INSTRuCT network.

For peer review only - http://bmjopen.bmj.com/site/about/guidelines.xhtml

Figure 1. Study flow chart

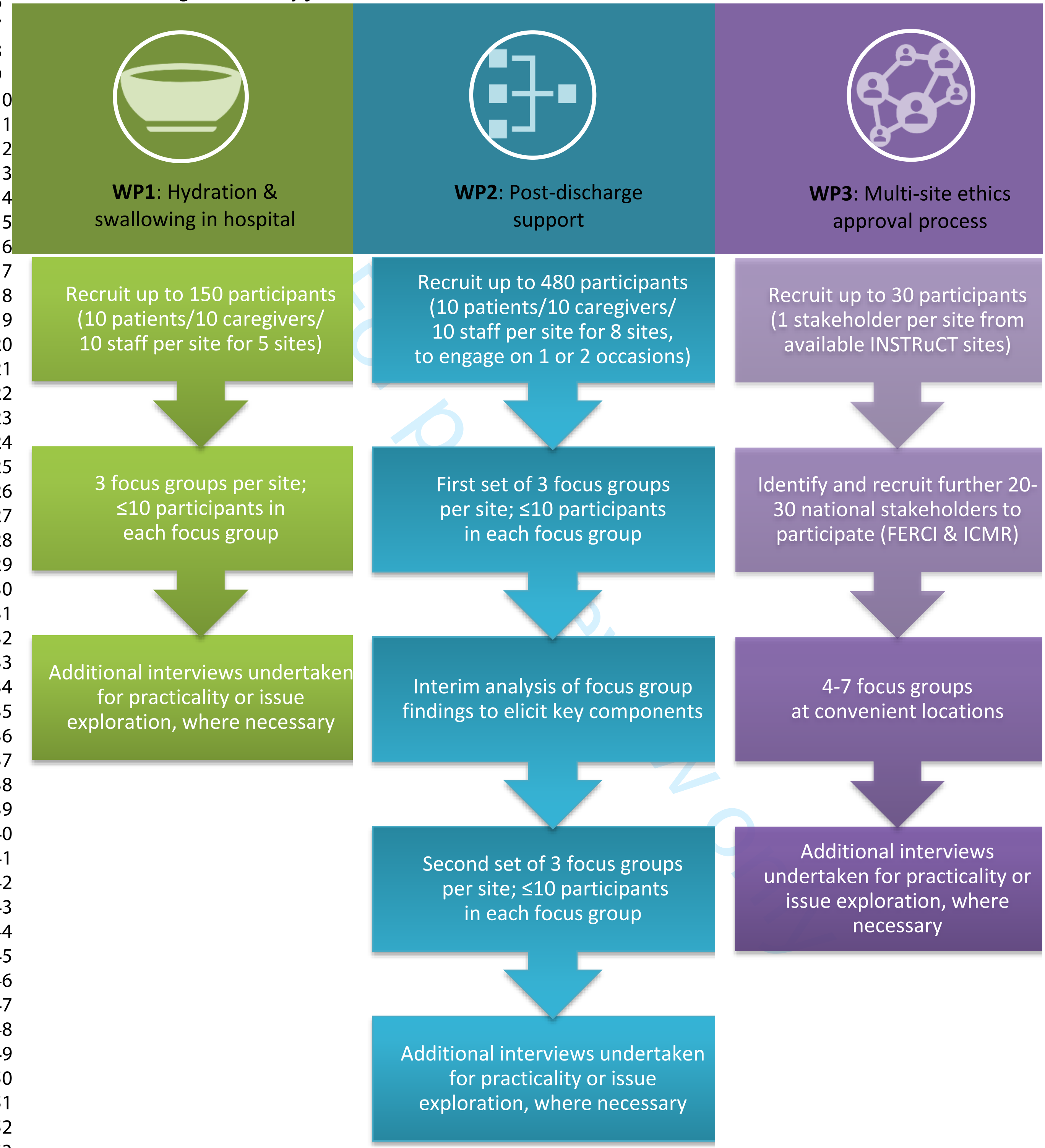

Protocol version: No. 1
Date: 29th June 2020

2.1 Overall participant timeline

Participants will be recruited between 1 November 2020 and 31 March 2021.

Figure 2. Study period timeline

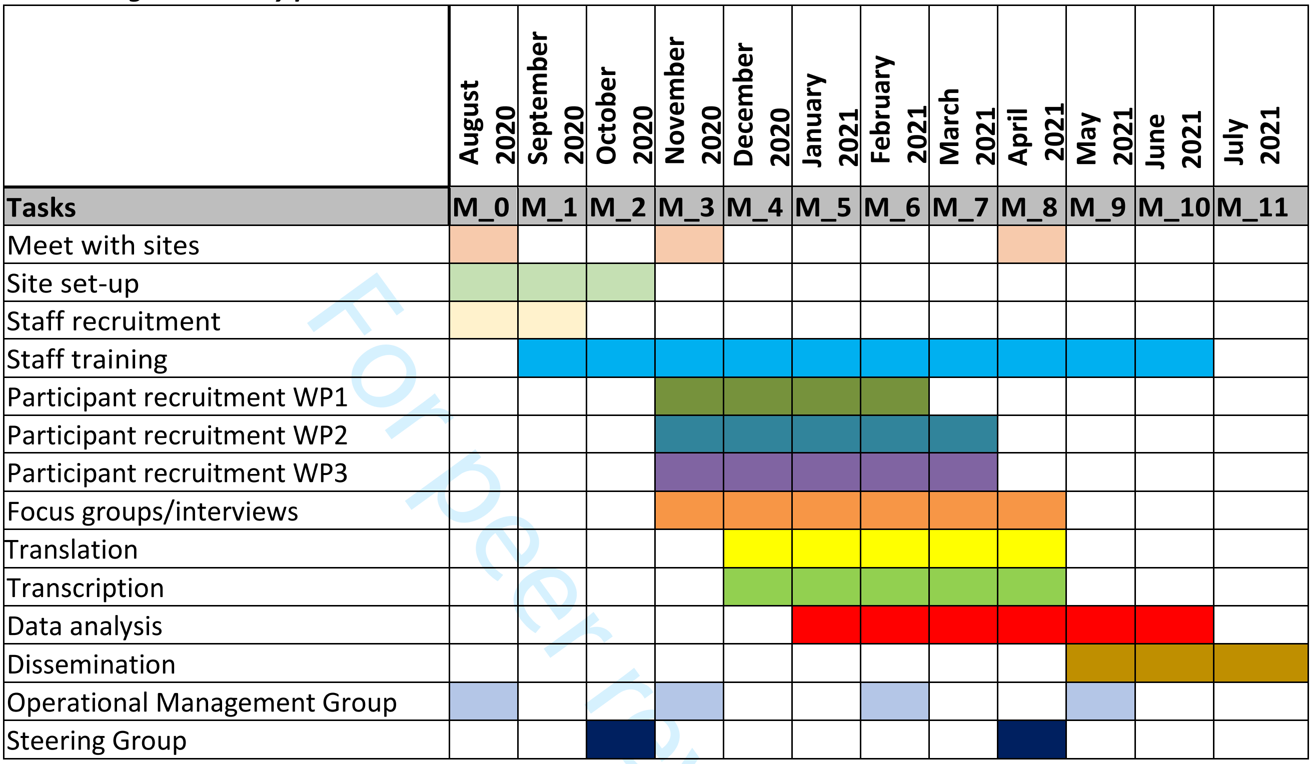

Protocol version: No. 1
Date: 29th June 2020

3. WP1 Protocol: Hydration and swallowing in hospital

3.1 WP1 aim and objectives

Aim: To explore and gain an understanding through interviews and focus groups of how stroke services are set up and operate, in order to identify how to implement a hydration and swallowing care bundle.

Objectives:

- To explore with stroke patients, families and staff, current screening, assessment and management for hydration and swallowing
- To understand challenges and solutions to the implementation of a hydration and swallowing management care bundle in hospital
- To understand regional differences
- To identify the key components for a potential implementation strategy of the hydration and swallowing management care bundle

3.2 WP1 study design

An exploratory study using qualitative research methods. Guided by a facilitator, focus groups will be conducted in local languages (with translators present to support participant engagement), with a note-taker and a secondary facilitator. Focus groups will explore topics including: experiences of access to and resources available for the assessment and management of hydration and swallowing; what people would want in relation to improving hydration and swallowing assessment and management within their locality and how this could be achieved. Focus groups will be conducted face-to-face or online using Zoom or similar technology. Semi-structured interviews will explore the same topics as in the focus groups; interviews may be undertaken to supplement focus group discussions, to follow up on focus group findings in more detail, and/or to ensure inclusivity where participant attendance at the focus groups is not feasible due to disabilities, clinical commitments and other practicalities. Interviews will also be conducted in local languages (with translators present, if required), and may be conducted either face-to-face or by telephone or using Zoom or similar technology (Topic guides Appendix 12 (staff members); Appendix 13 Patients/relatives).

3.3 WP1 study setting

This study will be conducted at five hospital sites across India:

For peer review only - http://bmjopen.bmj.com/site/about/guidelines.xhtml

1. All India Institute of Medical Sciences (AIIMS), Bhopal
2. Baptist Christian Hospital (BCH), Tezpur
3. Indira Gandhi Medical College & Hospital (IGMC), Shimla
4. National Institute of Mental Health and Neurosciences (NIMHANS), Bangalore
5. Zydus Hospital, Ahmedabad

3.4 WP1 participant eligibility

We will identify a convenience sample (using non-restrictive criteria) of patients and/or their relatives, and staff, with experience of stroke from a range of stroke services.

Subjects

We will recruit up to 150 participants Each site will host three focus groups, which include ≤10 participants in each staff, or patient and relative focus group (maximum individual commitment of 2-4 hours).

Patients

- Eligible for inclusion in the study are adult patients (aged ≥18 years) with experience of stroke or TIA.
- Excluded patients are those with no legally acceptable representative to consent

Relatives

- Eligible for inclusion in the study are adult (aged ≥18 years) relatives or caregivers for the person who has had the stroke or TIA.

Staff

Clinical staff (medical, nursing and therapy) will also be invited to participate.

3.5 WP1 consent procedure

The study will follow the consent procedure as listed in ICH GCP section 4.8 Informed Consent of Trial Subjects, Indian Council of Medical Research (ICMR), Ethical Guidelines for Biomedical Research on Human Participants and Indian Good Clinical Practices as laid down by Central Drugs Standard Control Organization (CDSCO).

Protocol version: No. 1
Date: 29th June 2020

For peer review only - http://bmjopen.bmj.com/site/about/guidelines.xhtml

Each patient/relative (Appendix 1 (WP1) and Appendix 5 (WP2)) and staff member (Appendix 3 (WP1)and Appendix 7 (WP2)) will be given a Participant Information Sheet (PIS) (which describes the study by a site RA or member of the clinical team. Participants agreeing to take part will be asked to provide written informed consent (Patients/relatives: Appendix 2 (WP1; Appendix 6 (WP2); Staff members: (Appendix 4 (WP1) and Appendix 8 (WP2)) before their focus group/interview; and, prior to any data being collected.

A legally acceptable representative may sign the consent form on behalf of an illiterate patient after verbally explaining the study details to them (illiterate patients to provide their thumb impression alongside signature of the legally acceptable representative). If neither a patient nor their legally acceptable representative are literate, an impartial witness (any person completely unrelated to the clinical study) may explain the details of the PIS to the patient and sign the consent form on behalf of the patient (who will provide their thumb impression alongside the signature of the impartial witness).

A copy of the consent form will be given to the participant. The original copy will be kept in the relevant Site File.

3.6 WP1 data collection

Demographic (age; sex; occupation) and stroke detail (type of stroke; severity of stroke [mild/moderate/severe]; experience of swallowing difficulties [current/recent/previous]; psychosocial difficulties (WP2 only); time since stroke; any disability [modified Rankin scale: MRS]) will be collected for all recruited participants from the patient and/or relative (Appendix 9). If this is not possible, this information will be recorded from the patient's medical records by a member of the clinical team.

Details of relationship to patient and age will be collected for recruited relatives in the consent form.

Job role, grade and place of work will be collected for all recruited staff members in the consent form.

3.7 WP1 outputs

- An understanding of current service provision and processes
- An understanding of barriers and facilitators from a range of perspectives

For peer review only

For peer review only

Protocol version: No. 1
Date: 29th June 2020

For peer review only - http://bmjopen.bmj.com/site/about/guidelines.xhtml

4. WP2 Protocol: Post-discharge support

4.1 WP2 aims and objectives

Aim: Through interviews and focus groups to:

- Map the current resources available for hydration and swallowing problems and psychosocial support following discharge
- Identify the key components of potential interventions for the management of hydration and swallowing, and psychosocial support post-discharge, and explore potential options for interventions with clinical staff, relatives of stroke patients, and stroke patients

Objectives:

- To explore patient pathways post-discharge and understand resources available for care
- To understand patients' and relatives' concerns, contextual challenges, and solutions
- To understand regional differences
- To identify key components of potential interventions

4.2 WP2 study design

An exploratory study using qualitative research methods. Guided by a facilitator, focus groups will be conducted in local languages (with translators present to support participant engagement), with a note-taker and a secondary facilitator. Focus groups will explore topics including: experiences of, access to and resources available for the on-going assessment and management of hydration and swallowing and post-discharge support; what people would want in relation to improving post-discharge support within their locality and how this could be achieved. Focus groups will be conducted face-to-face or online using Zoom or similar technology. Semi-structured interviews will explore the same topics as in the focus groups; interviews may be undertaken to supplement focus group discussions, to follow up on focus group findings in more detail, and/or to ensure inclusivity where participant attendance at the focus groups is not feasible due to disabilities, clinical commitments and other practicalities.

For peer review only - http://bmjopen.bmj.com/site/about/guidelines.xhtml

Interviews will also be conducted in local languages (with translators present, if required), and may be conducted either face-to-face or by telephone or using Zoom or similar technology. (Topic guides Appendix 14 (staff members); Appendix 15 Patients/relatives).

4.3 WP2 study setting

This study will be conducted at eight hospital sites in India, the five listed above and:

1. All India Institute of Medical Sciences (AIMS), New Delhi.
2. Christian Medical College (CMC), Ludhiana, Punjab.
3. Sree Chitra Tirunal Institute of Medical Sciences (SCTIMS), Trivandrum, Kerala.

Subjects

We will recruitment a maximum of 480 participants across the 8 sites. Each site will host a maximum of six focus groups, which include ≤10 participants (staff, or patient and relative focus group).

4.4 WP2 participant eligibility

We will identify a convenience sample (using non-restrictive criteria) of patients and/or their relatives, and staff, with experience of stroke from a range of stroke services.

Patients

- Eligible for inclusion in the study are adult patients (aged ≥18 years) discharged from hospital with experience of stroke.
- Excluded patients are those with no legally acceptable representative to consent

Relatives

- Eligible for inclusion in the study are adult (Aged ≥18 years) relatives or caregivers for the person who has had the stroke following discharge home from hospital.

Staff

Clinical staff (medical, nursing and therapy) will also be invited to participate.

Protocol version: No. 1
Date: 29th June 2020

For peer review only - http://bmjopen.bmj.com/site/about/guidelines.xhtml

4.5 WP2 participant recruitment

At each hospital site, the site Research Associates (RAs) and/or clinical team will invite patients, and any relatives required to support them, to take part in a focus group or interview; where possible these participants will reflect a range of characteristics, as applicable (age/sex/severity of stroke/experience of swallowing and/or psychosocial difficulties).

The site RAs will also invite a purposive sample of staff participants to represent a range of disciplines and grades of staff involved in the management of hydration and swallowing or post-discharge support.

Participants may choose to contribute to either one or two focus groups and/or interviews (maximum individual commitment of 2-4 hours).

4.6 WP2 consent procedure

As described in WP1.

4.7 WP2 data collection

As described in WP1.

4.8 WP2 outputs

- An understanding of current service provision and resources
- An understanding of the needs and preferences of patients and their relatives, and clinical staff, in relation to the management of hydration and swallowing, and psychosocial support following discharge from hospital
- Identification of the key components of interventions for supporting patients and their relatives following discharge from hospital

For peer review only - http://bmjopen.bmj.com/site/about/guidelines.xhtml

5. WP3 Protocol: Multi-site ethical approval process

5.1 WP3 aim and objectives

Aim: With key stakeholders, to map out the current ethics approval process, identify challenges, enablers and the feasibility of establishing a stream-lined multi-centre ethics approval process for stroke studies within the Indian Stroke Clinical Trial (INSTRuCT) network.

Objectives:

- To understand challenges
- To identify enhanced processes for ethical approval

5.2 WP3 study design

Design: an exploratory study using qualitative research methods.

Focus groups will be guided by a facilitator, conducted in local languages (with translators present to support participant engagement), with a note-taker and a secondary facilitator (if required). Focus groups will be conducted face-to-face at convenient locations or online using Zoom or similar technology. Focus group discussion topics will include: How the current ethics approval processes currently operates; what are the current challenges; are there any changes that the group like to see made to the ethics approval process and how might these be implemented.

Covering the same topics, semi-structured interviews may be undertaken to inform focus group discussions, to follow up on focus group findings in more detail, and/or to ensure inclusivity where participant attendance at focus groups is not feasible due restrictions on movement, other practicalities and clinical commitments. Interviews will also be conducted in local languages (with translators present to support participant engagement) and may be conducted either face-to-face or by telephone or video-conferencing technology (Stakeholder topic guide Appendix 16).

5.3 WP3 study setting

The INSTRuCT network relevant organisations, members of the Forum for Ethics Review Committees in India (FERCI) and the Indian Council of Medical Research (ICMR).

For peer review only - http://bmjopen.bmj.com/site/about/guidelines.xhtml

5.4 WP3 participant eligibility

Key stakeholders will be invited to participate in focus groups and interviews as follows:

- Employed by an appropriate INSTRuCT site, within either stroke or ethics unit
- From relevant organisations, such as the Forum for Ethics Review Committees in India (FERCI) and the Indian Council of Medical Research (ICMR)
- Actively involved in developing multi-centre research ethics processes though IMPROVIS-ATION

For peer review only

5.5 WP3 participant recruitment

We will recruitment a maximum of 60 participants (30 INSTRuCT network site stakeholders (one per site) and up to 30 FERCI and/or ICMR stakeholders). The programme manager will invite site stakeholders to take part, and organisational stakeholders (FERCI/ICMR) will self-nominate themselves to participate after initial approach through the INSTRuCT network.

Between 4-7 focus groups will be conducted at convenient locations, which include ≤10 participants in each focus group (any combination of available stakeholders; maximum individual commitment of 2-4 hours).

The findings from the focus groups and interviews will be synthesised and presented at a final discussion group to identify the key components of any changes and to agree next steps.

5.6 WP3 data collection

Job role, place of work and ethics committee experience information will be collected for all recruited key stakeholders in the consent form.

5.7 WP3 consent procedure

Each identified key stakeholder will be given a PIS describing the study (Appendix 10) by a member of the research team, issued via e-mail. Any key stakeholder agreeing to take part will be asked to provide written informed consent (Appendix 11) prior to their participation in a focus group or interview.

5.8 WP3 outputs

- An understanding of the current ethics approval processes
- Identification of potential challenges, enablers, and feasibility of developing a stream-lined multi-centre ethics approval process for stroke studies within the INSTRuCT network.

For peer review only

Protocol version: No. 1
Date: 29th June 2020

For peer review only - http://bmjopen.bmj.com/site/about/guidelines.xhtml

6. Overall operations across work packages

6.1 Analysis

Demographic data will be analysed descriptively. Focus groups/Interviews will be transcribed, and then analysed utilising NVivo (a qualitative data analysis computer software package) to support the analysis process. Framework will be used, this permits identification and cross-classification of variables through: identification of key concepts, mapping their range and diversity, interpreting patterns of association and investigating possible reasons. This would give a local service provider perspective on barriers/facilitators to implementing the potential pathways/reforms in the future, ensuring services fit needs. Discussion notes will also be collated and summarised.

6.2 Data management

Data will be treated and stored according to the General Data Protection Regulation (Information Commissioners Office 2018), the Data Protection Act (2018), Caldicott Principles (Caldicott 2013) and the National Institute for Health Research (NIHR) Good Clinical Practice (GCP) Standards.

Written consent forms will be stored in each hospital's Site File (WPs 1 and 2) and at CMC, the Co-ordinating Centre (WP3). All other data will be stored electronically in password-protected files. Members of the research team will have access to project files and data as appropriate to their role.

Participants will be assigned a participant number, this will be used to anonymise their data. Their anonymised data will be typed up into electronic format and stored in a secure OneDrive folder. Audio files from digital voice recorders will be translated, translations checked and transcribed. Once the transcription has been checked the audio file will be deleted. Anonymised transcriptions will be stored on the secure University of Central Lancashire network for five years following completion of the study in-line with the UCLan Data Protection Code of Practice.

Any individuals' names mentioned in the focus group/interview discussions will be removed from transcription. It will not be possible to recognise individuals from information in the completed data analysis or write up of final reports and publications, and any direct quotes used will remain anonymous.

For peer review only - http://bmjopen.bmj.com/site/about/guidelines.xhtml

6.3 Monitoring

The Project Co-ordinator from CMC and Research Programme Manager at the University of Central Lancashire will monitor training, recruitment and consent procedures.

Monitoring will facilitate confirmation of site adherence to this protocol and Good Clinical Practice (GCP) Guidelines, relevant local and regional ethical requirements, data accuracy and quality. The study may also be audited by external government or regulatory authorities.

In WPs 1 and 2, each site is responsible for maintaining a Site File containing a delegation log, a training log, and validity of consent log. In WP3, CMC will retain a Site File containing: a delegation log and validity of consent log.

Access to files will be made available by all participating sites for monitoring and audit purposes. On-site monitoring will be undertaken by the University of Central Lancashire in the form of (i) scheduled site visits during the study; or, (ii) triggered site visits in response to concerns relating to any study research processes.

6.4 Ethics and regulatory approval

The work packages, developed under this protocol, will be designed and implemented to comply with Ethical Principles for Medical Research Involving Human Patients (World Medical Association Declaration of Helsinki), Ethical Guidelines for Biomedical Research on Human Participants by Indian Council of Medical Research (ICMR), UK

Ethical and Research Governance approval will be obtained from individual sites' ethics committees and The University of Central Lancashire. Health Ministry Screening Committee (HMSC) approval will be sought from ICMR. If appropriate, the study will be registered on www.ctri.nic.in (Clinical Trials Registry-India (CTRI)) before study (patient-related) activities commence.

The CI is responsible for communicating any protocol amendments to the site PI. The site PI is responsible for ensuring that amendments are implemented, and that all documents replaced by 'new' versions are destroyed immediately. Site staff need to be informed of, and start using, new documentation as soon as local approvals are in place.

The site PI is responsible for reporting any protocol violations and routine reporting

For peer review only - http://bmjopen.bmj.com/site/about/guidelines.xhtml

outcomes, reporting to the CI and to their own ethical and governing bodies according to the requirements of the study protocol and written approval of the Ethics Committee.

Consent withdrawal

Participants may withdraw from the study without providing a reason. In these circumstances, the participant will be asked if we can retain and use their data. The PIS emphasises participation is voluntary and that people have the right to withdraw up until they take part in a focus group/discussion, or to the point where their data is anonymised after taking part in an interview and the data becomes fully anonymised once the data have been combined for analysis

6.5 Adverse events & safeguarding

Any adverse events directly resulting from the study will be reported to the site PI (who will inform the Programme Manager in the UK), in accordance with ICH Good Clinical Practices.

Any identified safeguarding concerns will be managed in accordance with the policies and procedures at each individual site.

6.6 Dissemination plan

A comprehensive dissemination plan will be developed with stakeholders, including outputs in clinical and academic peer-reviewed journals, conferences and social media outlets.

Study-generated data will be made available to other researchers for secondary research purposes, as agreed by the partnership and in accordance with open access requirements. This process will be managed and assured by the University of Central Lancashire, in accordance with institutional policy and procedures.

6.7 Study management

The study will be managed in accordance with the governance structure and processes in place for the NIHR Global Health Research Group (GHRG).

For peer review only - http://bmjopen.bmj.com/site/about/guidelines.xhtml

Professor Dame Caroline Watkins is the Director of the NIHR GHRG, with support from Deputy Directors: Professor Liz Lightbody, Deputy Director (Nursing Stroke), UCLAN; Professor Jeyaraj Pandian, Deputy Director (Neurology), Christian Medical College; Dr Pallab Maulik, Deputy Director (Psychiatry), The George Institute for Global Health. Members of the group have worked collaboratively to deliver a number of innovative large-scale multi-disciplinary studies in stroke including in the UK and India. Further details of the group are available at uclanglobalhealth.co.uk

The progress of the project will be overseen every three months by the Operational Management Group (OMG), which will ensure effective operational management of the project with reference to agreed work plans, milestones and key deliverables. The OMG consists of the Director, Deputy Directors, Work Package and site leads, and the Programme Manager.

The overall delivery of the programme will be overseen by the Steering Group, which provides oversight, advice and support to the project team. The Steering Group will make recommendations relating to project delivery to the funder and project team, undertake periodic reviews, and is responsible for the content of formal reports to the programme funder (NIHR). The group consists of a Chair, Directors, work package leads, representative of the funder, and other experts independent of the programme team. The Steering Group will meet regularly, approximately every 3-6 months depending on the level of activity in the programme.

For peer review only

For peer review only - http://bmjopen.bmj.com/site/about/guidelines.xhtml

7. References

Bray B, Ayis S, Campbell J, Hoffman A, Roughton M, Tyrrell PJ, Wolfe CDA, Rudd AG Associations between the organisation of stroke services, process of care, and mortality in England: prospective cohort study. BMJ. 2013, 346

Cadilhac DA, Andrew NE, Lannin NA, Middleton S, Levi CR, Dewey HM, Grabsch B, Faux S, Hill K, Grimley R, Wong A, Sabet A, Butler E, Bladin CF, Bates TR, Groot P, Castley H, Donnan GA, Anderson CS and Australian Stroke Clinical Registry C. Quality of Acute Care and Long-Term Quality of Life and Survival: The Australian Stroke Clinical Registry. Stroke. 2017;48:1026-1032.

Dalal PM. Burden of stroke: Indian perspective. International Journal of Stroke. 2006; 1:164-6

MacKay J, Mensah G. Atlas of Heart Disease and Stroke. World Health Organisation. 2004.

Pandian JD, Sudhan P. Stroke epidemiology and stroke care services in India. Journal of Stroke. 2013;15(3):128-134.

Raju RS, Kaur P and Pandian JD. Psychosocial problems, quality of life, and caregiver burden among stroke caregivers in India. International Journal of Stroke. 2012;7: 100-101. doi:10.1111/j.1747-4949.

Stroke Unit Trialists' Collaboration. Organised inpatient (stroke unit) care for stroke. Cochrane Database of Systematic Reviews. 2013, Issue 9. Art. No.: CD000197

The ATTEND Collaborative Group. Family-led rehabilitation after stroke in India (ATTEND): A randomised controlled trial. The Lancet, 2017; 390:10094. doi.org/10.1016/S0140-6736(17)31447-2.

The Stroke Association. State of the Nation. Stroke statistics. 2016

World Health Organisation. Global status report on non-communicable diseases. 2014. WHO Press, Switzerland.

Protocol version: No. 1
Date: 29th June 2020

For peer review only - http://bmjopen.bmj.com/site/about/guidelines.xhtml

For peer review only

COREQ (COnsolidated criteria for REporting Qualitative research) Checklist

A checklist of items that should be included in reports of qualitative research. You must report the page number in your manuscript where you consider each of the items listed in this checklist. If you have not included this information, either revise your manuscript accordingly before submitting or note N/A.

Topic	Item No.	Guide Questions/Description	Reported on Page No.
**Domain 1: Research team and reflexivity**			
Personal characteristics			
Interviewer/facilitator	1	Which author/s conducted the interview or focus group?	
Credentials	2	What were the researcher's credentials? E.g. PhD, MD	
Occupation	3	What was their occupation at the time of the study?	
Gender	4	Was the researcher male or female?	
Experience and training	5	What experience or training did the researcher have?	
Relationship with participants			
Relationship established	6	Was a relationship established prior to study commencement?	
Participant knowledge of the interviewer	7	What did the participants know about the researcher? e.g. personal goals, reasons for doing the research	
Interviewer characteristics	8	What characteristics were reported about the inter viewer/facilitator? e.g. Bias, assumptions, reasons and interests in the research topic	
**Domain 2: Study design**			
Theoretical framework			
Methodological orientation and Theory	9	What methodological orientation was stated to underpin the study? e.g. grounded theory, discourse analysis, ethnography, phenomenology, content analysis	
Participant selection			
Sampling	10	How were participants selected? e.g. purposive, convenience, consecutive, snowball	
Method of approach	11	How were participants approached? e.g. face-to-face, telephone, mail, email	
Sample size	12	How many participants were in the study?	
Non-participation	13	How many people refused to participate or dropped out? Reasons?	
Setting			
Setting of data collection	14	Where was the data collected? e.g. home, clinic, workplace	
Presence of non-participants	15	Was anyone else present besides the participants and researchers?	
Description of sample	16	What are the important characteristics of the sample? e.g. demographic data, date	
Data collection			
Interview guide	17	Were questions, prompts, guides provided by the authors? Was it pilot tested?	
Repeat interviews	18	Were repeat inter views carried out? If yes, how many?	
Audio/visual recording	19	Did the research use audio or visual recording to collect the data?	
Field notes	20	Were field notes made during and/or after the inter view or focus group?	
Duration	21	What was the duration of the inter views or focus group?	
Data saturation	22	Was data saturation discussed?	
Transcripts returned	23	Were transcripts returned to participants for comment and/or	

For peer review only

Topic	Item No.	Guide Questions/Description	Reported on Page No.
		correction?	
**Domain 3: analysis and findings**			
Data analysis			
Number of data coders	24	How many data coders coded the data?	
Description of the coding tree	25	Did authors provide a description of the coding tree?	
Derivation of themes	26	Were themes identified in advance or derived from the data?	
Software	27	What software, if applicable, was used to manage the data?	
Participant checking	28	Did participants provide feedback on the findings?	
Reporting			
Quotations presented	29	Were participant quotations presented to illustrate the themes/findings? Was each quotation identified? e.g. participant number	
Data and findings consistent	30	Was there consistency between the data presented and the findings?	
Clarity of major themes	31	Were major themes clearly presented in the findings?	
Clarity of minor themes	32	Is there a description of diverse cases or discussion of minor themes?	

Developed from: Tong A, Sainsbury P, Craig J. Consolidated criteria for reporting qualitative research (COREQ): a 32-item checklist for interviews and focus groups. *International Journal for Quality in Health Care*. 2007. Volume 19, Number 6: pp. 349 – 357

Once you have completed this checklist, please save a copy and upload it as part of your submission. DO NOT include this checklist as part of the main manuscript document. It must be uploaded as a separate file.

For peer review only

BMJ Open

Healthcare professionals' perspectives of the provision of, and challenges for, eating, drinking and psychological support post-stroke: findings from semi-structured interviews across India.

Journal:	*BMJ Open*
Manuscript ID	bmjopen-2022-069150.R1
Article Type:	Original research
Date Submitted by the Author:	28-Mar-2023

Complete List of Authors:	Jones, Stephanie ; University of Central Lancashire<br>Injety, Ranjit ; Christian Medical College and Hospital Ludhiana<br>Pandian, Jeyaraj ; Christian Medical College and Hospital Ludhiana, Neurology<br>Ratra, Sanjali; Christian Medical College and Hospital Ludhiana<br>Sylaja, PN; Sree Chitra Tirunal Institute for Medical Sciences and Technology, Neurology<br>Babu, Veena; Sree Chitra Tirunal Institute for Medical Sciences and Technology<br>Srivastava, Padma; All India Institute of Medical Sciences<br>Sharma, Sakshi; All India Institute of Medical Sciences<br>Sharma, Sudhir; Indira Gandhi Medical College<br>Kaushal, Pawna; Indira Gandhi Medical College<br>Webster, Jemin; Baptist Christian Hospital Tezpur<br>Koirala, Amrit; Baptist Christian Hospital Tezpur<br>Kulkarni, Girish Baburao; National Institute of Mental Health and Neuro Sciences<br>Dixit, Anand; National Institute of Mental Health and Neuro Sciences<br>Sharma, Arvind; Zydus Research Center<br>Prajapati, Jagruti; Zydus Research Center<br>Weldon, Jo; University of Central Lancashire<br>Kuroski, Jennifer A; University of Central Lancashire<br>Watkins, Caroline; University of Central Lancashire College of Health and Wellbeing, Faculty of Health and Wellbeing<br>Lightbody, Catherine; University of Central Lancashire, School of Nursing

**Primary Subject Heading**:	Global health
Secondary Subject Heading:	Health services research, Qualitative research
Keywords:	Stroke < NEUROLOGY, STROKE MEDICINE, QUALITATIVE RESEARCH, REHABILITATION MEDICINE

SCHOLARONE™
Manuscripts

I, the Submitting Author has the right to grant and does grant on behalf of all authors of the Work (as defined in the below author licence), an exclusive licence and/or a non-exclusive licence for contributions from authors who are: i) UK Crown employees; ii) where BMJ has agreed a CC-BY licence shall apply, and/or iii) in accordance with the terms applicable for US Federal Government officers or employees acting as part of their official duties; on a worldwide, perpetual, irrevocable, royalty-free basis to BMJ Publishing Group Ltd ("BMJ") its licensees and where the relevant Journal is co-owned by BMJ to the co-owners of the Journal, to publish the Work in this journal and any other BMJ products and to exploit all rights, as set out in our licence.

The Submitting Author accepts and understands that any supply made under these terms is made by BMJ to the Submitting Author unless you are acting as an employee on behalf of your employer or a postgraduate student of an affiliated institution which is paying any applicable article publishing charge ("APC") for Open Access articles. Where the Submitting Author wishes to make the Work available on an Open Access basis (and intends to pay the relevant APC), the terms of reuse of such Open Access shall be governed by a Creative Commons licence – details of these licences and which Creative Commons licence will apply to this Work are set out in our licence referred to above.

Other than as permitted in any relevant BMJ Author's Self Archiving Policies, I confirm this Work has not been accepted for publication elsewhere, is not being considered for publication elsewhere and does not duplicate material already published. I confirm all authors consent to publication of this Work and authorise the granting of this licence.

For peer review only

Title: Healthcare professionals' perspectives of the provision of, and challenges for, eating, drinking and psychological support post-stroke: findings from semi-structured interviews across India.

Authors' names and affiliations

Stephanie P Jones[1], Ranjit J Injety[2], Jeyaraj D Pandian[2], Sanjali Ratra[2], P N Sylaja[3], Veena Babu[3], M V Padma Srivastava[4], Sakshi Sharma[4], Sudhir Sharma[5], Pawna Kaushal[5], Jemin J Webster[6], Amrit Koirala[6], Girish Baburao Kulkarni[7], Anand Dixit[7], Arvind Sharma[8], Jagruti Prajapati[8], Jo Catherine Weldon[1], Jennifer A Kuroski[1], Caroline Leigh Watkins[1], Catherine Elizabeth Lightbody[1] on behalf of the NIHR Global Health Research Group on IMPROVIng Stroke CarE in India (IMPROVISE) Collaboration.

[1]University of Central Lancashire, Preston, Lancashire

[2]Christian Medical College and Hospital, Neurology, Ludhiana, India,

[3]Sree Chitra Tirunal Institute for Medical Sciences and Technology, Neurology, Thiruvananthapuram, India,

[4]All India Institute of Medical Sciences, Neurology, New Delhi, India,

[5]IGMC Shimla, Neurology, Shimla, India,

[6]Baptist Christian Hospital, Department of Medicine, Tezpur, India,

[7]NIMHANS Bangalore, Neurology, Bangalore, India,

[8]Zydus Hospitals And Healthcare Research Pvt. Ltd, Neurology, Ahmedabad, India.

Named contact & address/affiliation
Dr Stephanie Jones
E-mail. Sjones10@uclan.ac.uk
Tel. +44 1772 895107
University of Central Lancashire, Preston, Lancashire, UK

Funder: National Institute for Health Research

Key words: Stroke, Post-discharge, Qualitative, India

Word count: 4092

For peer review only

Abstract

Aim: This qualitative study explores with health professionals the provision of, and challenges for, post-discharge stroke care, focussing on eating, drinking and psychological support across India.

Design: Qualitative semi-structured interviews.

Setting: Seven geographically diverse hospitals taking part in a Global Health Research Programme on Improving Stroke Care in India

Participants: A purposive sample of healthcare professionals with current experience of working with stroke patients.

Results: Interviews with 66 healthcare professionals (23 nurses (14 staff nurses; 7 senior nurse officers; 1 Intensive Care Unit nurse; 1 palliative care nurse)); 16 doctors (10 neurologists; 6 physicians); 10 physiotherapists; 5 speech and language therapists; 4 occupational therapists; 4 dieticians; 2 psychiatrists; and 2 social workers) resulted in three main themes: Integrated inpatient discharge care planning processes; Post-discharge patient and caregiver role and challenges; Patient and caregiver engagement post-discharge.

Conclusions: Discharge planning was integrated and customised, although resources were limited in some sites. Task-shifting compensated for a lack of specialists but was limited by staff education and training. Caregivers faced challenges in accessing and providing post-discharge care. Post-discharge care was mainly hospital-based, supported by tele-services, especially for rural populations. Further research is needed to understand post-discharge care provision and the needs of stroke survivors and their caregivers.

For peer review only - http://bmjopen.bmj.com/site/about/guidelines.xhtml

Strengths and limitations of this study

- We did not seek to interview HCPs based in the community as services varied between the included sites and were not stroke-specific.
- Due to the diversity of the included hospitals we were unable to make comparisons across services.
- It is acknowledged that one in every three patients with stroke do not access appropriate healthcare due to non-affordability, usage of alternative medicines, and difficulty in conveyance to hospital.
- Participants represented a broad range of stroke services; of which, over a third were nurses of varying seniority.
- Interview methods (telephonic or face-to-face) and interviews in local languages maximised participant engagement, and also enabled adherence to COVID-protocols, and inclusion of participants under COVID restrictions at the point of interview.

For peer review only

For peer review only - http://bmjopen.bmj.com/site/about/guidelines.xhtml

Background

Stroke is a significant global health problem and a major cause of mortality and morbidity in high income countries (HICs), and increasingly in low-middle income countries (LMICs)[1]. Seventy percent of strokes occur in LMICs; the subsequent disease burden is greater than in HICs[2]. Life expectancy in India has recently increased to over 60 years of age[3,4] leading to an increase in age-related, non-communicable diseases including stroke[5,6]. In India, the incidence of stroke ranges between 105 and 152/100,000 people per year[7]. Increasing incidence of stroke is observed among younger groups, with first ever strokes occurring around the age of 57 years [8–10], increasing families' burden.

Stroke survivors often experience many disabilities including hemiparesis, cognitive and communication problems, incontinence, dysphagia, and psychological trauma[11], having serious economic and social impacts[12]. In India, the majority of stroke survivors have disabilities, and the burden of long-term care often falls on family members [9]. This is particularly challenging when stroke survivors leave hospital with complex needs; requiring nasogastric tubes, catheters, and support with communication and psychological problems.

Stroke rehabilitation has been found to reduce mortality, decrease institutionalised care[13], and increase functional outcomes[14,15]. Despite the known benefits, India has limited resources to provide post-discharge stroke care[16]. Accessing rehabilitation services is a challenge for many stroke survivors in India. There are enduring barriers including long delays in accessing rehabilitation services, poor transport between home and hospital[17,18], and a lack of trained personnel[9]. There is a lack of data about post-discharge stroke services and the support available for patients and caregivers[14].

The resources for post-stroke rehabilitation, especially the therapy workforce and infrastructure, are very limited in many LMICs[19]. Beyond urban tertiary centres, rehabilitation services are scarcely available. There are <10 skilled rehabilitation practitioners per 1 million people in many LMICs, which does not allow specialization to treat a particular condition or disorders[20]. In India, it estimated that there are only 41,567 PTs, 3,100 OTs, and 1,700 SLTs, and these are geographically concentrated in urban areas leaving much of the nation without resources[15]. Given the scarcity of resources, the rehabilitation needs of the stroke survivors, especially in LMICs, remain largely unmet resulting in a growing need for cost-effective, multidisciplinary rehabilitation services, particularly post-discharge[21].

This qualitative study aimed to explore with health professionals the provision of, and challenges for, post-discharge stroke focussing on eating, drinking and psychological support across India.

For peer review only - http://bmjopen.bmj.com/site/about/guidelines.xhtml

Design

Qualitative semi-structured interviews.

Setting and subjects

Interviews were conducted with a purposive sample of HCPs (doctors, nurses, physiotherapists [PTs], occupational therapists [OTs], speech and language therapists [SLTs], dieticians, psychiatrists and social workers [SWs]) with current experience of working with stroke patients at seven geographically diverse hospitals taking part in a Global Health Research Programme on Improving Stroke Care in India (All India Institute of Medical Sciences, New Delhi; Baptist Christian Hospital, Tezpur; Christian Medical College, Ludhiana; Indira Gandhi Medical College & Hospital, Shimla; National Institute of Mental Health and Neurosciences, Bangalore; Sree Chitra Tirunal Institute of Medical Sciences, Trivandrum; Zydus Hospital, Ahmedabad) between July and December 2021.

Methods

Interviews explored HCPs' views of stroke patient management in preparation for discharge and post-discharge support for patients and caregivers, focussing on eating, drinking and psychological support.

The interview guide (Supplementary File 1) was informed by published research and developed collaboratively by the research team. While not formally piloted, the interview guide was revised following feedback from Research Associates (RAs) and Principal Investigators (PIs) at the included hospitals, to ensure that questions could be translated into regional languages (Assamese, Gujarati, Hindi and Malayalam). According to participant preference, interviews were conducted in English or regional languages, and took place either face-to-face or by telephone (dependent on current COVID-19 restrictions).

Data collection

All interviews were digitally recorded, translated (as required) and transcribed verbatim. All transcriptions were verified by RAs prior to analysis. Data collection continued until saturation, determined as no new information provided from across the participants recruited.

For peer review only - http://bmjopen.bmj.com/site/about/guidelines.xhtml

Patient and Public Involvement

Patients and the public were involved in discussions to agree the priority areas that were the focus of post-discharge support interviews with healthcare professionals (eating, drinking and psychological support).

Analysis

Each transcript was read in full, and data explored using inductive coding and analysed using a reflexive thematic method. Thirty interviews were coded as a group and 36 by individual project members. Confirmative and contradictory results were discussed by the project team for consensus. Data were analysed thematically using NVivo software (version 1.5.2.). This manuscript was prepared in accordance with the Consolidated Criteria for Reporting Qualitative Research (COREQ) guidelines[22] (Supplementary File 2).

Reflexivity

All interviews were undertaken by RAs (AD, AK, JP, PK, SR, SS, VB) who were known by participants, but were not someone who they worked with clinically and lasted between 15 and 47 minutes. Interviews were conducted in Assamese (n=1), Gujarati (n=1), Hindi (n=12), Malayalam, (n=3) and English (n=51). All members of the project team were involved in data analysis. Dependability of the data may vary based on any staffing changes following the research undertaken, and all interviews took place during the COVID-19 pandemic. Interviews were undertaken with participants from seven hospitals across geographically diverse areas. To support the analysis, a supplementary mapping exercise was undertaken to identify hospital and community-based services. The findings from the mapping activity enabled cross-validation of the interview findings.

Ethical considerations

Ethics approval (REF: 2020-9715) was granted by the Health Ministry's Screening Committee (HMSC), India and each hospital's ethics committee. The study conforms to ethical standards of the Indian Council of Medical Research Guidelines[23]. In the UK, ethical approval was granted by the Science, Technology, Engineering and Medicine Ethics Committee, University of Central Lancashire (STEM 939). Informed consent was obtained from all participants prior to interview.

Results

Sixty-six HCPs participated, 39 (59%) female, representing a range of health professionals (Table 1).

For peer review only - http://bmjopen.bmj.com/site/about/guidelines.xhtml

Table 1: Participant demographics

**Healthcare Professionals**	**N (%)**
**Nurses**	**23 (35%)**
Staff Nurses	14 (21%)
Senior nurse officers	7 (11%)
Intensive care nurse	1 (1.5%)
Palliative care nurse	1 (1.5%)
**Doctors**	**16 (24%)**
Neurologists	10 (15%)
Physicians	6 (9%)
Physiotherapists	10 (15%)
Speech and language therapists	5 (8%)
Occupational therapists	4 (6%)
Dieticians	4 (6%)
Psychiatrists	2 (3%)
Social workers	2 (3%)

Three themes were identified:

1. Integrated Inpatient Discharge Care Planning Processes (5 sub-themes):
    - 1.1. Healthcare System and Resources
    - 1.2. Staff Identification, and Expectations of Caregivers
    - 1.3. Task-shifting
    - 1.4. Patient and Caregiver Training
    - 1.5. Education and Support in Preparation for Discharge
2. Post-Discharge Patient and Caregiver Roles and Challenges (3 sub-themes):
    - 2.1. Patient and Caregiver Characteristics
    - 2.2. Caregiver Roles
    - 2.3. Caregiver Challenges
3. Patient and Caregiver Engagement post-discharge (2 sub-themes):
    - 3.1. Post-Discharge Support - Hospital
    - 3.2. Post-Discharge Support - Community-based

For peer review only

Theme 1: Integrated Discharge Care Planning Processes

Discharge planning processes were integrated throughout the patient pathway, beginning soon after admission although varied across sites. Discharge planning was frequently dependent on the availability of specialist staff, resources and facilities, and often resulted in task-shifting due to a lack of staff availability/expertise. Participants reported that HCPs provided patients and caregivers with tailored, targeted education, training, and support throughout hospitalisation.

Sub-theme 1.1. Healthcare System and Resources

All sites had localised, inpatient discharge planning processes or protocols in place, but this was largely undocumented.

"We have discharge processes that the team work to but we don't have any written protocol but we are observing the patient from the admission." (01-05)

Staff identified that existing post-discharge management systems could be improved by formal documentation to facilitate a more consistent and comprehensive approach.

*"I really feel like there is dire need of formulating a kind of discharge protocol for the patient." (*03-02*)*

Sub-theme 1.2. Staff Identification, and Expectations of Caregivers

Identified by the clinical team, caregivers would most often be the spouse, another immediate or extended family member.

"We will ask about the immediate relatives, either a wife or husband, brother or sister, son or daughter. We also identify the immediate family or caregiver." (06-04)

Training took place with patients and caregivers expecting that caregivers would primarily support and provide post-discharge care.

"*They need to know how to give physiotherapy, occupational therapy, mental support, how to give food, speech related, so, they need to be a doctor, they need to be therapist, they need to be a nurse… If the caregiver is not up to the mark, the rehabilitation fails.*" (06-01)

On admission, an individualised approach was used to identify the most appropriate caregiver(s) for tailored training and support.

For peer review only - http://bmjopen.bmj.com/site/about/guidelines.xhtml

"It depends on the status of the patient according to the medical and surgical condition of patient… We educate the relatives and patients as well as possible." (07-03)

In addition to, or in the absence of, family caregivers, some patients employed the services of professional caregivers.

"Some patients have the capacity of paying, so they hire the physiotherapists, and they call them at their home…Some patients cannot pay but they need physiotherapy." (04-04)

Sub-theme 1.3. Task-shifting

Whilst some sites had well-resourced stroke teams, there was a shortage of dieticians, SWs and therapists (PTs, OTs, SLTs, psychologists) at other sites, where existing staff, predominantly nurses, provided information and support.

"At the time of discharge being a nurse, as we do not have speech and language therapist, then we [nurses] try to put patients in the upright position and ask them to speak as much as they can." (04-02)

"*We are at such a place where we do not get any psychologists, that thing [psychological problems] we [nurses] have to manage ourselves because we are very close to the patients*." (04-04)

Staff often described task-shifting as integral to their professional roles and responsibilities; however, the majority had not undertaken any formal training and had developed knowledge and skills largely through experiential learning.

"No, we didn't get any specific training for this [providing dietary advice]. Whatever we have studied during our nursing training, learned from doctors, and from the internet, on that basis we provide this information." (04-01)

Sub-theme 1.4. Patient and Caregiver Training

Patient and caregiver training usually began at admission and continued until discharge, with staff encouraging caregivers to observe all aspects of patient care, as required, including the preparation and administration of medication, positioning, changing dressings, feeding, the management of nasogastric (NG) tubes, maintaining hygiene, and rehabilitation exercises.

"We make sure the person who will be taking care of the patient at home is there from the beginning, so that they are very much part of all the processes that happen in the ward: changing dressings, changing position, and feeding and all those things." (02-06)

For peer review only - http://bmjopen.bmj.com/site/about/guidelines.xhtml

For peer review only

Staff felt that patients and caregivers should be prepared both mentally and physically for discharge but shared few examples of any formal psychological support or preparation.

"We tell them, start observing from now, because when you go home, you have to do it by yourself. So, they also get ready mentally and physically. (02-02)

Sub-theme 1.5. Education and Support in Preparation for Discharge

To prepare the patient and caregiver for discharge, training delivered by HCPs was predominantly provided through verbal instruction and explanation.

"When the relatives are in the hospital, we teach them verbally and some of the relatives they themselves write down [instructions]." (02-01)

Training was often supplemented by the provision of information, in a variety of formats including leaflets, videos, written instructions, diet charts, and discharge summaries. Some PTs provided pre-recorded videos of exercises, available through websites such as YouTube and/or recorded patients undertaking exercises.

"But then we do use videos like I said, sometimes pictures of exercises, but it's not uniform for all. (03-05)

Whilst training took place throughout patients' hospitalisation, education and information was largely provided within 24-48 hours of discharge. Leaflets were available in some sites, in most local languages, covering a range of topics to support the caregiver. In other sites discharge summaries and diet charts were the only source of written information and instruction.

"*In our stroke unit, we have made pamphlets regarding safe swallow techniques and how to feed the patient."* (06-02)

Accessible written information in appropriate languages was viewed as a valuable source of information, enabling information to be passed from the patient or primary caregiver in hospital to the main caregiver at home.

"We have pamphlets which are given to the patient depending on the language of their preference… that helps in dispersing information further because the caregiver who is there with the patient in the hospital may not be the same caregiver who is there at home." (01-04)

For peer review only - http://bmjopen.bmj.com/site/about/guidelines.xhtml

2. Post-Discharge Patient and Caregiver Roles and Challenges (3 sub-themes):

The patient's stroke and patients'/caregivers' levels of literacy were viewed as important factors in the subsequent 'successes' of post-discharge rehabilitation. HCPs identified multiple challenges in providing education and training, and specific challenges for the patients and caregivers themselves.

Sub-theme 2.1: Patient and Caregiver Characteristics

Female, family members were often the preferred caregivers for both male and female patients.

"W*e prefer training the female relative because the boys or males they usually go out for work.*" (02-01)

Stroke severity, post-stroke complications, cognitive and communication problems influenced the complexity and challenges of education and training for patients and their caregivers.

"*Some patients having pressure sores, second or third degree, then it will be really difficult to handle the wound, the relatives need more information about how to care for the wound, how to reduce the severity, when to change dressings, and how much time between changing the patient's position.*" (07-02)

Patients' and caregivers' educational attainments were viewed as important in effective training delivery. Patients and caregivers from rural locations often had lower levels of literacy that impacted their ability to understand, and to pay for post-discharge care needed.

"Those who come from village they are not educated and are daily wage workers who are below the poverty-line… they really cannot understand the importance of [rehabilitation] exercises." (02-03)

Sub-theme: 2.2. Caregiver Roles

Participants described post-discharge stroke care as largely the role of home-based caregivers (mainly family members) who had responsibility for a range of tasks, including rehabilitation, the prevention of complications and subsequent stroke, and the provision of psychosocial support. Caregivers supported activities of daily living such as maintaining hygiene, food preparation tailored to the patient's swallowing ability and dietary requirements (e.g. diabetes, low salt, lower calorie); the preparation and administration of medication; rehabilitation (recommended by PT or aimed at improving speech and/or cognition); facilitating follow-up appointments; seeking help in an emergency; and supporting the patient's psychological/emotional needs.

For peer review only - http://bmjopen.bmj.com/site/about/guidelines.xhtml

For peer review only

"They have to do the medication on time. They have to do the proper positioning, proper personal hygiene to the patient, and then they have to maintain the proper diet to the patient, to give nourishment to the patients. The entire role in the hospital, that has to be continued in the home also." (01-06)

Sub-theme 2.3. Caregiver Challenges

Hospital staff provided support and education to an identified caregiver in hospital in preparation for discharge, although these trained caregivers were not always the primary caregiver at home.

"*Sometimes in rural areas women do not come when their husband is admitted in the hospital and the caretaker is different, so all these things happen, and I feel very bad.*" (01-10)

Staff reported that caregivers encountered a multitude of challenges including: financial constraints in healthcare expenses and loss of income; a lack of time to provide care around work commitments; varying levels of literacy, ability to comprehend, and to adhere to instructions; difficulties transporting the patient to hospital appointments and so resorting to nearer sources of help (including from local healthcare centres (with or without trained professionals), informal help (pharmacists), alternative medicine (AUYSH) and unqualified professionals (faith-healers, quacks); and a dearth of community-based services. Challenges were particularly marked among rural populations.

"*One of the negative aspects of patients being in rural areas is that most caregivers go out to work in daytime... They are unable to give enough time for patient care and exercise.*" (02-03)

Theme 3: Patient and Caregiver Engagement post-discharge

Provided at discharge, follow-up appointments were individualised and based on the patient's level of disability, co-morbidities and available post-discharge support. In some sites, dedicated stroke helplines, Stroke Unit contact details, or individual staff members' phone numbers were provided.

Sub-theme 3.1. Post-Discharge Support – Hospital

Staff reported that it was common for patients to have multiple follow-up appointments with their doctor, who was able to provide advice, treatment and referral to other services, as needed. Follow-up appointments usually took place at one-month post-discharge but varied according to patient need. However, some patients had challenges in attending follow-up appointments, mainly due to the distance to the hospital from their home and travel costs.

For peer review only - http://bmjopen.bmj.com/site/about/guidelines.xhtml

"The patient comes after one month or if the patient is outside of the area, he may come after two months, three months. That is up to the patient. That is up to their condition. That is up to their circumstances." (01-01)

For patients who were asked to, and who were able to, attend follow-up, referral to specialist support was made available if needed or if this was part of on-going treatment.

"Once they come to come back for follow-up in the OPD they are sent to physiotherapy OPD for review. (02-03)

Tele-support was variable in availability and purpose. Staff reported that patients and caregivers were more likely to contact the hospital post-discharge when the patient had complex health needs (from their stroke and/or co-morbidities). The most common reasons for seeking help through teleservices were problems with NG tubes (feeding and blockages); advice about preparing food in different consistencies and condition specific diets (low sugar, low salt); questions about medication (dosage, frequency and side-effects); catheter care; monitoring or changes in blood pressure; clarifications about PT-based exercises; and, psychological problems (including anxiety around recurrent stroke and the impact of stroke on life expectancy).

"The patients are getting discharged, we give them our number. So, at this moment, we are trying to attend to them as much as possible with the phone. So, if they have any problem for example, if there is a catheter problem, or there is NG change we try to solve this through the phone." (02-05)

Some hospitals were able to refer patients with ongoing rehabilitation needs to rehabilitation clinics and networks; and where the patient lived some distance from the hospital to local healthcare facilities.

"There are three options. One is the inpatient programme, once they are medically stable. The second is to get admitted under a rehabilitation package. We have 7, 10 and 30-day packages where they stay in hospital and get rehabilitation. The next option is we have guest houses outside [the hospital] and they can stay there at very minimal prices and they then come on an OPD basis to the physiotherapist. The last option is our network of physios visit them at home or we send them videos through WhatsApp." (03-05)

At some hospitals there were also post-discharge outreach facilities (including palliative care and community nurses), but at others there were no such resources.

For peer review only - http://bmjopen.bmj.com/site/about/guidelines.xhtml

"We have palliative care nurses who do a lot of home visits, twice weekly, if there is any patient who is bedridden and fully dependent, those patients, we connect them to the palliative care nurses. Palliative care nurses visit them at home and change NG tubes, catheters, dressings and many more of those things." (02-01)

"We arrange a particular community level therapist, and we have that contact number too, so they can simply contact us regarding the patient's issues." (06-06)

Sub-theme: 3.2. Post-Discharge Support – Community-based

Outside of hospital-based care, post-discharge support services (such as counselling/psychological support and physiotherapy) were not stroke-specific and were considered by staff to deliver little additional benefit. Broader post-discharge support (outside hospital) was reportedly lacking in availability.

"There are some people who get people to massage [by private physiotherapists] and all the therapy techniques that were taught they will not use, they will use their own technique and do oil massage. So, it doesn't strengthen them, a weakness is there." (02-02)

Patients and caregivers, particularly in rural areas were often reported to have accessed alternative medicine, unqualified 'Doctors' or traditional faith healers post-discharge.

"The community will support more after discharge, they usually do so at the time of rituals. Pujari [priest] will advise many things that are advantageous or disadvantageous." (05-01)

Discussion

To our knowledge, this is the first qualitative study to explore post-discharge stroke care with HCPs providing stroke services across India. This study identified a number of strengths including comprehensive individualised treatment planning, task-shifting (defined as the rational redistribution of tasks among health workforce teams) where there was a lack of specialists, comprehensive patient and caregiver training, and post-discharge care facilitated by hospital, and community-based services, where available. However, there were also some significant challenges.

Inpatient discharge care-planning processes usually began soon after admission but were often not formally documented, a process which may have facilitated a more consistent and comprehensive approach. The provision of tailored and individualised information, education and training for patients

For peer review only - http://bmjopen.bmj.com/site/about/guidelines.xhtml

and caregivers was often dependant on staff and resource availability, which commonly resulted in task-shifting. Task-shifting describes a situation where a task is transferred to a HCP with a different or lower level of education and training, or to a person specifically trained to perform limited tasks[24]. As not all hospitals in the study had comprehensive MDTs, with reported shortages of dieticians, SWs and therapy staff, in these instances, nurses would often take on the roles of other HCPs despite limited formal training. Whilst task-shifting often enables a more efficient use of available human resources and can constitute a robust strategy to provide stroke care[25] there is currently no documented evidence of stroke-specific task-shifting training for non-neurologist HCPs, with HCPs reporting that they largely developed knowledge and skills through informal or experiential learning.

~~The resources for post-stroke rehabilitation, especially the therapy workforce and infrastructure, are very limited in many LMICs[23]. Beyond urban tertiary centres, rehabilitation services are scarcely available. There are <10 skilled rehabilitation practitioners per 1 million people in many LMICs, which does not allow specialization to treat a particular condition or disorders[24]. In India, it estimated that there are only 41,567 PTs, 3,100 OTs, and 1,700 SLTs, and these are geographically concentrated in urban areas leaving much of the nation without resources[15]. Given the scarcity of resources, the rehabilitation needs of the stroke survivors, especially in LMICs, remain largely unmet resulting in a growing need for cost-effective, multidisciplinary rehabilitation services, particularly post-discharge[25].~~

Caregivers, often female family members, were responsible for all aspects of care and activities of daily living post-discharge, results echoed in previous research[26]. Their ability to deliver required healthcare and psychosocial support was often dependent on access to services (geographically and financially), and the availability of other family members generating income in their (or the stroke survivors) absence. Congruent with other reports, HCPs identified challenges in training and education, and consequently difficulties for caregivers when stroke survivors had more severe strokes, requiring complex care[27]. Whilst caregivers may have experienced increased demands, and in some cases limited preparedness for their new roles, unlike research from developed countries[28], this was viewed by HCPs to be a necessary responsibility rather than a burden.

As post-discharge care is frequently the responsibility of family members in LMICs[29], the preparation of caregivers was a vital aspect of in-hospital discharge planning. Training was usually delivered verbally, sometimes supported by alternative formats provided in local languages to enhance accessibility, comprehension and adherence. However, patient and caregiver characteristics (demographic, socio-economic, stroke severity) and social roles (employment status, family structure and dynamics), all had an impact on the subsequent accessibility, availability and provision of post-discharge care. Caregiver literacy was viewed as an importance aspect of successful post-discharge care. Previous research has demonstrated that low functional literacy may contribute to poor health

For peer review only - http://bmjopen.bmj.com/site/about/guidelines.xhtml

status through a reduced ability to use written instructions and advice, and reduced adherence to medication and rehabilitation regimens[30]. Low literacy levels were viewed as a particular challenge in rural areas.

Post-discharge support provided by hospitals included multiple follow-up appointments, the frequency and number determined by the patient's condition. Post-discharge care included further service referrals (to rehabilitation clinics, local healthcare centres or post-discharge outreach facilities where available). However, post-discharge support beyond hospital settings was lacking in the community, as a result alternative medicine, unlicensed practitioners and faith-healers were often sought, particularly among rural populations. In addition, other non stroke-specific rehabilitation services with limited patient benefit were relied upon. Complementary and alternative medicine (CAM) approaches for post-stroke recovery are frequently accessed in India and often include ayurvedic massage, herbal medicine, marma therapy, reiki therapy, homeopathy, intravenous fluids, and opium[31].

Further data and research are needed to understand the support available and needs of stroke survivors and caregivers, to inform the development of innovative post-stroke interventions that are accessible, patient-centered, and culturally sensitive[18].

This study has a number of limitations. Firstly, the study did not seek to interview HCPs based in the community as these services varied between the included sites and were not stroke-specific. Due to the diversity of the included hospitals we were unable to make comparisons across services. Hospital staff broadly lacked awareness of the spectrum of stroke care services available in-hospital (outside of their own profession) and in the community. Finally, it is acknowledged that one in every 3 patients with stroke do not access appropriate healthcare due to non-affordability, usage of alternative medicines, and difficulty in conveyance to hospital[9].

We have also identified several strengths: Participants represented a broad range of stroke services; of which, over a third were nurses of varying seniority. Interviews were conducted in local languages to maximise engagement and by providing participants with a choice of interview method (telephonic or face-to-face), this enabled adherence to COVID-protocols, and supported inclusion of participants under COVID restrictions at the point of interview.

Conclusion

Discharge planning was integrated and customised but in some sites there were limited resources.

Task-shifting among HCPs compensated for a lack of specialists, but limitations were identified in staff education and training. Caregivers faced many challenges in both accessing and providing post-discharge care. Post-discharge care was mainly hospital-based, supported by tele-services, especially

For peer review only - http://bmjopen.bmj.com/site/about/guidelines.xhtml

for rural populations. Further research is needed to gain an understanding of post-discharge care provision and the needs of stroke survivors and their caregivers.

Funding statement

This research was funded by the National Institute for Health Research (NIHR) [Global Health Research Group on Improving Stroke Care, University of Central Lancashire (16/137/16)] using UK aid from the UK Government to support global health research. The views expressed in this publication are those of the author(s) and not necessarily those of the NIHR or the UK government.

Competing interests statement

There are no conflicts of interest to declare.

Data availability statement

No additional data available.

Author contributions

Authors CEL, PMV, PJD, SPN and CLW contributed to the study conception and study design. Data collection was undertaken by PK, SR, SS, AK, AD, JP, VB . Analysis was performed by SJ, RI, JW, WK, PK, SR, SS, AK, AD, JP, CEL, PMV, PJD, SPN, CLW, JW, AS, SS, GK. The first draft of the manuscript was written by SJ, RI and JW. All authors commented on previous versions of the manuscript. All authors read and approved the final manuscript.

Acknowledgements

We would like to thank the health professionals who took the time to take part in this study. We would also like to thank the members of the NIHR Global Health Research Group on IMPROVIng Stroke CarE in India (IMPROVISE) Collaboration.

For peer review only

References

1. Feigin VL, Forouzanfar MH, Krishnamurthi R, et al. Global and regional burden of stroke during 1990-2010: findings from the Global Burden of Disease Study 2010. *Lancet*. 2014;383(9913):245-254. doi:10.1016/s0140-6736(13)61953-4

2. Roth GA, Abate D, Abate KH, et al. Global, regional, and national age-sex-specific mortality for 282 causes of death in 195 countries and territories, 1980–2017: a systematic analysis for the Global Burden of Disease Study 2017. *The Lancet*. 2018;392(10159):1736-1788. doi:10.1016/S0140-6736(18)32203-7

3. Yamunadevi A, Sulaja S. Old Age Mortality in India –An Exploration from Life Expectancy at Age 60. *International Journal of Asian Social Science*. 2016;6(12):698-704. doi:10.18488/journal.1/2016.6.12/1.12.698.704

4. Horton R, Das P. Indian health: the path from crisis to progress. *The Lancet*. Published online 2011. doi:10.1016/S0140-6736(10)62179-4

5. Dalal P, Bhattacharjee M, Vairale J, Bhat P. UN millennium development goals: Can we halt the stroke epidemic in India? *Annals of Indian Academy of Neurology*. 2007;10(3):130. doi:10.4103/0972-2327.34791

6. Naik K. Challenges in delivering stroke care in India. *Indian j health sci*. 2016;9(3):245. doi:10.4103/2349-5006.196342

7. Jones SP, Baqai K, Clegg A, et al. Stroke in India: A systematic review of the incidence, prevalence, and case fatality. *International Journal of Stroke*. 2022;17(2):132-140. doi:10.1177/17474930211027834

8. Pandian JD, Jaison A, Deepak SS, et al. Public awareness of warning symptoms, risk factors, and treatment of stroke in northwest India. *Stroke*. 2005;36(3):644-648. doi:10.1161/01.STR.0000154876.08468.a0

9. Pandian JD, Sudhan P. Stroke epidemiology and stroke care services in India. *J Stroke*. 2013;15(3):128-134. doi:10.5853/jos.2013.15.3.128

10. Tripathi M, Padhy UP, Vibha D, et al. Predictors of refractory epilepsy in north India: a case-control study. *Seizure*. 2011;20(10):779-783. doi:10.1016/j.seizure.2011.07.010

11. Lloyd J, Pinto AM, Nair S, Tarey S. A Qualitative Study on Palliative Needs of Stroke Patients in an Indian Tertiary Care Setting - Doctors' Perspective. *Indian J Palliat Care*. 2019;25(1):84-91. doi:10.4103/IJPC.IJPC_161_18

12. Walsh ME, Galvin R, Loughnane C, Macey C, Horgan NF. Community re-integration and long-term need in the first five years after stroke: results from a national survey. *Disability and Rehabilitation*. 2015;37(20):1834-1838. doi:10.3109/09638288.2014.981302

13. Langhorne P, Ramachandra S, Stroke Unit Trialists' Collaboration. Organised inpatient (stroke unit) care for stroke: network meta-analysis. *Cochrane Database Syst Rev*. 2020;4:CD000197. doi:10.1002/14651858.CD000197.pub4

14. Lindley RI, Anderson CS, Billot L, et al. Family-led rehabilitation after stroke in India (ATTEND): a randomised controlled trial. *The Lancet*. 2017;390(10094):588-599. doi:10.1016/S0140-6736(17)31447-2

For peer review only

15. Pandian JD, Gandhi DBC, Lindley RI, Bettger JP. Informal Caregiving. *Stroke*. 2016;47(12):3057-3062. doi:10.1161/STROKEAHA.116.013701

16. Sylaja PN, Srivastava MVP, Shah S, et al. The SARS-CoV-2/COVID-19 pandemic and challenges in stroke care in India. *Ann N Y Acad Sci*. 2020;1473(1):3-10. doi:10.1111/nyas.14379

17. Banerjee TK, Das SK. Fifty years of stroke researches in India. *Ann Indian Acad Neurol*. 2016;19(1):1-8. doi:10.4103/0972-2327.168631

18. Kamalakannan S, Gudlavalleti Venkata M, Prost A, et al. Rehabilitation Needs of Stroke Survivors After Discharge From Hospital in India. *Arch Phys Med Rehabil*. 2016;97(9):1526-1532.e9. doi:10.1016/j.apmr.2016.02.008

19. World Health Organization, World Heart Federation, World Stroke Organization. Global atlas on cardiovascular disease prevention and control. *Global atlas on cardiovascular disease prevention and control*. Published online 2011:155-155.

20. World Health Organization. *Global Diffusion of EHealth: Making Universal Health Coverage Achievable: Report of the Third Global Survey on EHealth*. World Health Organization; 2017.

21. Bonita R, Beaglehole R. Stroke prevention in poor countries: time for action. *Stroke*. 2007;38(11):2871-2872. doi:10.1161/STROKEAHA.107.504589

22. Tong A, Sainsbury P, & Craig J. Consolidated criteria for reporting qualitative research (COREQ): A 32-item checklist for interviews and focus groups. *International Journal for Quality in Health Care*, 2017;19(6), 349–357. https://doi.org/10.1093/intqhc/ mzm042

23. Guidelines | Indian Council of Medical Research | Government of India. Indian Council of Medical Research. Published 2017. Accessed June 29, 2022. https://main.icmr.nic.in/content/guidelines-0

24. Joshi R, Alim M, Kengne AP, et al. Task shifting for non-communicable disease management in low and middle income countries--a systematic review. *PLoS One*. 2014;9(8):e103754. doi:10.1371/journal.pone.0103754

25. Lindsay P, Furie KL, Davis SM, Donnan GA, Norrving B. World Stroke Organization global stroke services guidelines and action plan. *International journal of stroke : official journal of the International Stroke Society*. 2014;9 Suppl A100. doi:10.1111/ijs.12371

26. Mandowara B, Patel AN, Amin AA, Phatak A, Desai S. Burden Faced by Caregivers of Stroke Patients Who Attend Rural-based Medical Teaching Hospital in Western India. *Ann Indian Acad Neurol*. 2020;23(1):38-43. doi:10.4103/aian.AIAN_406_18

27. Rigby H, Gubitz G, Phillips S. A Systematic Review of Caregiver Burden following Stroke. *International Journal of Stroke*. 2009;4(4):285-292. doi:10.1111/j.1747-4949.2009.00289.x

28. LeLaurin JH, Lamba AH, Eliazar-Macke ND, et al. Postdischarge Intervention for Stroke Caregivers: Protocol for a Randomized Controlled Trial. *JMIR Res Protoc*. 2020;9(11):e21799. doi:10.2196/21799

29. Das SK, Banerjee TK, Biswas A, et al. A Prospective Community-Based Study of Stroke in Kolkata, India. *Stroke*. 2007;38(3):906-910. doi:10.1161/01.STR.0000258111.00319.58

30. Easton P, Entwistle VA, Williams B. Health in the "hidden population" of people with low literacy. A systematic review of the literature. *BMC Public Health*. 2010;10(1):459. doi:10.1186/1471-2458-10-459

For peer review only

For peer review only - http://bmjopen.bmj.com/site/about/guidelines.xhtml

31. Pandian JD, Toor G, Arora R, et al. Complementary and Alternative Medicine Treatments Among Stroke Patients in India. *Topics in Stroke Rehabilitation*. 2012;19(5):384-394. doi:10.1310/tsr1905-384

For peer review only

Supplementary File 1: WP2 Interview Guide

Q1. Can you tell me about your role?

Q2. Can you tell me about how your service currently manages patients when they have been discharged from hospital?

Q3. What options do patients have if they need support for their stroke after they have been discharged from hospital?

Q4. Are there any services available to support patients or their relatives in relation to their eating and drinking after they have been discharged from hospital?

Q5. Are there any services available to support patients or their relatives in relation to their psychological adjustment to the stroke after they have been discharged from hospital?

Q6. What works well about support that is available for patients in the community? Are there any aspects that do not work as well?

Q7. What role do the patient's family/relatives have in caring for patients when they have been discharged from hospital?

Q8. Do the patient's family/relatives receive any information about how to provide care for their relative who has had a stroke?

Q9. Do the patient's family/relatives receive any information about how to provide care for their relative who has had a stroke?

Q10. What works well about involving the patient's family/relatives once they are in the community?

Q11. Are there any aspects that do not work well?

Q12. Is there anything else that you would like to add?

Thank you for taking the time to take part in this interview.

For peer review only - http://bmjopen.bmj.com/site/about/guidelines.xhtml

Study title: **The National Institute for Health Research Global Health Research Group on improving stroke care in India at The University of Central Lancashire - Advancing the INSTRuCT Operations and Network.**

Short title: ***IMPROVIng Stroke care in India - Advancing The INSTRuCT Operations and Network*** **(IMPROVIS-ATION)**

Protocol version: No. 1
Date: 29th June 2020

For peer review only

For peer review only - http://bmjopen.bmj.com/site/about/guidelines.xhtml

Contents

i. Study team – Roles & Contact details 4
ii. Study summary 5
iii. Abbreviations 7
1. Background 8
2. Aims of the research 11
Figure 1. Study flow chart 12
2.1 Overall participant timeline 13
Figure 2. Study period timeline 13
3. WP1 Protocol: Hydration and swallowing in hospital 14
3.1 WP1 aim and objectives 14
3.2 WP1 study design 14
3.3 WP1 study setting 14
3.4 WP1 participant eligibility 15
3.5 WP1 consent procedure 15
3.6 WP1 data collection 15
3.7 WP1 outputs 156
4. WP2 Protocol: Post-discharge support 186
4.1 WP2 aims and objectives 186
4.2 WP2 study design & methods 187
4.3 WP2 study setting 197
4.4 WP2 participant eligibility 197
4.5 WP2 participant recruitment 17
4.6 WP2 consent procedures 198
4.7 WP2 data collection 18
4.8 WP2 outputs 18
5. WP3 Protocol: Multi-site ethical approval process 219
5.1 WP3 aim and objectives 219
5.2 WP3 study design 219
5.3 WP3 study setting 20
5.4 WP3 participant eligibility 20
5.5 WP3 participant recruitment 20
5.6 WP3 data collection 20
5.7 WP3 consent procedure 20
5.8 WP3 outputs 20

For peer review only

Protocol version: No. 1
Date: 29th June 2020

6. Overall operations across work packages 24
6.1 Analysis 24
6.2 Data management 24
6.3 Monitoring 25
6.4 Ethics and regulatory approval 25
6.5 Adverse events & safeguarding 263
6.6 Dissemination plan 26
6.7 Study management 26
7. References 288
8. Appendices and topic guides 289

For peer review only

Protocol version: No. 1
Date: 29th June 2020

i. Study team – Roles & Contact details

Name	Role (*Specialism*)	Institution	Contact
Professor Dame Caroline Leigh Watkins	Director	UCLan	CLWatkins@uclan.ac.uk
Denise Forshaw	Deputy Director (*Operations and Governance, Lancashire CTU*)	UCLan	DForshaw@uclan.ac.uk
Professor Catherine Elizabeth Lightbody	Deputy Director (*Nursing; Stroke*)	UCLan	CELightbody@uclan.ac.uk
Dr Pallab Maulik	Deputy Director (*Psychiatry*)	George Institute For Global Health	PMaulik@georgeinstitute.org.in
Professor Jeyaraj Pandian	Deputy Director (*Neurology*)	Christian Medical College	jeyarajpandian@hotmail.com
Dr Liz Boaden	Collaborator (*Stroke*)	UCLan	EBoaden1@uclan.ac.uk
Professor Dominique Cadilhac	Collaborator (*Nursing; Research*)	Monash University, Australia	dominique.cadilhac@monash.edu
Professor Andy Clegg	Collaborator (*Health services*)	UCLan	AClegg3@uclan.ac.uk
Professor Mark Gabbay	Collaborator (*Health services*)	University of Liverpool	M.B.Gabbay@liverpool.ac.uk
Rachel Georgiou	Collaborator (*Stroke*)	UCLan	RGeorgiou@uclan.ac.uk
Dr Jo Gibson	Collaborator (*Stroke*)	UCLan	JGibson4@uclan.ac.uk
Professor Maree Hackett	Collaborator (*Epidemiology*)	UCLan	maree.hackett@sydney.edu.au
Dr Yogeshwar Kalkonde	Collaborator (*Neurology*)	Society for Education, Action and Research in Community Health, India	yvkalkonde@gmail.com
Professor Caroline Sanders	Collaborator (*Patient and Carer engagement*)	The University of Manchester	Caroline.Sanders@manchester.ac.uk
Professor Padma Srivastava	Collaborator (*Stroke*)	All India Institute of Medical Sciences	vasanthapadma123@gmail.com
Professor PN Sylaja	Collaborator (*Stroke*)	Sree Chitra Tirunal Institute for Medical Sciences and Technology	sylajapn@sctimst.ac.in
Kamran Baqai	Research Associate	UCLan	KBaqai1@uclan.ac.uk
Dr Steph Jones	Programme Manager	UCLan	SJones10@uclan.ac.uk
Dr Gordon Prescott	Lead Statistician	UCLan	GPrescott1@uclan.ac.uk
Jo Weldon	Research Associate	UCLan	jcweldon@uclan.ac.uk

Protocol version: No. 1
Date: 29th June 2020

For peer review only - http://bmjopen.bmj.com/site/about/guidelines.xhtml

ii. Study summary

**Title**	**IMPROVI**ng **S**troke care in India **- A**dvancing **T**he **I**NSTRUCT **O**perations and **N**etwork (**IMPROVIS-ATION**)
**Design**	Exploratory studies
**Aim**	Work package 1 (WP1) aim:<br>• To explore and gain understanding of how stroke services are set up and operate in order to identify how to implement a hydration and swallowing care bundle.<br><br>Work package 2 (WP2) aims:<br>• To map the current resources available to manage hydration and swallowing problems and psychosocial support following discharge.<br>• Identify the key components of potential interventions for the management of hydration and swallowing, and psychosocial support post-discharge, and explore potential options for interventions with clinical staff, relatives of stroke patients and stroke patients.<br><br>Work package 3 (WP3) aim:<br>• With key stakeholders, to map out the current ethics approval process, identify challenges, enablers and the feasibility of establishing a stream-lined multi-centre ethics approval process for stroke studies within the Indian Stroke Clinical Trial (INSTRuCT) network.
**Population**	WP1 & 2: Convenience sample of stroke patients and/or their relatives and staff from a range of stroke services.<br><br>WP3: Key stakeholders from INSTRuCT network, Forum for Ethics Review Committees in India (FERCI), and the Indian Council of Medical Research (ICMR)
**Research methods**	Qualitative methods – focus groups and interviews.<br><br>WP1: Focus groups and/or interviews- around 10 members of staff, 10 patients and 10 relatives at 5 sites will be included (n=≤150)<br><br>WP2: Focus groups and/or interviews- around 10 members of staff, 10 patients and 10 relatives at 8 sites will be included. Focus groups will be held on two occasions (n=≤480 overall)<br><br>WP3: Focus groups and/or interviews involving around 10 people will take place at convenient locations with around 60 key stakeholders; identified from across 30 hospital sites and 20-30 key stakeholders from organisations such as FERCI and ICMR (n=≤60).
**Outputs**	WP1:<br>• An understanding of current service provision and processes<br>• An understanding of barriers and facilitators to the implementation of a hydration and swallowing care bundle from a range of perspectives<br><br>WP2:<br>• An understanding of current service provision and resources<br>• An understanding of the needs and preferences of patients and their relatives, and clinical staff in relation to the management of hydration and swallowing and psychosocial support following discharge from hospital<br>• Identification of key components of interventions for supporting patients and their relatives following discharge from hospital

Protocol version: No. 1
Date: 29th June 2020

For peer review only - http://bmjopen.bmj.com/site/about/guidelines.xhtml
For peer review only

	WP3:<br>• An understanding of the current ethics approval processes<br>• Identification of potential challenges, enablers, and feasibility of developing a stream-lined multi-centre ethics approval process for stroke studies within the INSTRuCT network.
**Duration**	1 year (1 August 2020 – 31 July 2021)
**Sponsor**	The University of Central Lancashire
**Funder**	National Institute of Health Research (NIHR)
**Chief Investigator**	Professor Dame Caroline Leigh Watkins
**Collaborating Institutes**	• University of Central Lancashire, UK<br>• University of Manchester, UK<br>• University of Liverpool, UK<br>• Monash University, Australia<br>• Christian Medical College, Ludhiana, India<br>• All India Institute of Medical Sciences, New Delhi, India<br>• Sree Chitra Tirunal Institute of Medical Sciences, Trivandrum, India<br>Research sites<br>• All India Institute of Medical Sciences (AIIMS), Bhopal, India<br>• Baptist Christian Hospital (BCH), Tezpur, India<br>• Indira Gandhi Medical College & Hospital (IGMC), Shimla, India<br>• National Institute of Mental Health and Neurosciences (NIMHANS), Bangalore, India<br>• Zydus Hospital, Ahmedabad, India<br>• Government Hospital Trivandrum, India

For peer review only

For peer review only - http://bmjopen.bmj.com/site/about/guidelines.xhtml

iii. Abbreviations

**CDSCO**	Central Drugs Standard Control Organization
**CI**	Chief Investigator
**FERCI**	Forum for Ethics Research Committees in India
**GCP**	Good Clinical Practice
**HMSC**	Health Ministry Screening Committee
**ICMR**	Indian Council of Medical Research
**IMPROVISE**	IMPROVIng StrokE care in India
**IMPROVIS-ATION**	IMPROVIng Stroke care in India - Advancing The INSTRuCT Operations and Network
**INSTRuCT**	Indian Stroke Clinical Trial Network
**NIHR**	National Institute for Health Research
**NBM**	Nil By Mouth
**NGT**	Nasogastric tube
**PI**	Principal Investigator
**SAE**	Serious Adverse Event
**SLT**	Speech and Language Therapist
**STKG**	Stakeholder Group
**WP**	Work Package

For peer review only

1. Background

Stroke is a recognized global health challenge. Cardiovascular disease including stroke is now the most common cause of death globally (World Health Organisation, 2014). India is one of the three countries with the greatest number of recorded stroke deaths worldwide (Mackay and Mensah, 2004). People in India typically suffer a first stroke at a much younger age than in the UK (India, age 57 (Pandian and Sudhan, 2013); UK, age 72 (male) and age 78 (female) (The Stroke Association 2018), leading to significant socio-economic burden (Dalal, 2006).

In the last 20 years, the UK, along with other higher income countries, has achieved significant improvements in stroke outcomes, including reduced mortality and morbidity. Much of this improvement has been through the introduction of organised stroke unit care. Stroke unit care has great potential to reduce mortality and morbidity worldwide, as it is the most widely applicable and effective intervention (Stroke Unit Trialist Collaboration, 2013).

One element of stroke unit care that is thought to be effective is an organised specialist multidisciplinary team approach and patient-centered care. It has been demonstrated that better adherence to several processes of care in acute stroke is associated with reduced mortality. A recent audit (Bray et al., 2013) showed that the performance of a swallow assessment, and provision of adequate fluid and nutrition, were all associated with lower 30-day mortality. Adherence to these and other care quality indicators may be amenable to improvement via structured intervention.

One way of delivering structured interventions is via care bundles. Care bundles aim to improve standards of care and patient outcomes by promoting the consistent implementation of a group of effective interventions, which when performed together, may have a better outcome than if performed individually (Cadilhac et al., 2017). They can be used to ensure the delivery of the minimum standard of care. One of the key elements of acute stroke care which are suitable for implementation via care bundles are hydration and swallowing.

In this study we will build on the National Institute for Health Research Global Health Research Group funded project Improving Stroke Care in India (IMPROVISE), in which a Care Bundle was

For peer review only - http://bmjopen.bmj.com/site/about/guidelines.xhtml

implemented to prevent dehydration in hospital, and to improve staff competence to assess dysphagia and manage oral intake. This reduced the need for patients screening positive for dysphagia to stay nil by mouth (NBM) to avoid aspiration pneumonia. These elements rely heavily on having appropriately educated and skilled nurses, working within a multidisciplinary team, to perform them effectively. IMPROVISE has introduced training and care pathways to allow staff to follow the initial dysphagia screen with comprehensive assessment to inform in-hospital management of oral intake. In this study we would like to explore and gain an understanding of how stroke services are set up and operate in order to identify how to implement a hydration and swallowing care bundle through interviews and discussions across 5 hospitals.

During exploration of how best to support patients and families to engage in in-hospital care, the issue of psychosocial support post-discharge has been raised; there is little available. Our co-applicants' ATTEND Trial also found that the trial Rehabilitation Therapists were hampered in their efforts to provide rehabilitation support because patients and families wanted instead to focus on psychosocial issues (The ATTEND Collaborative Group, 2017). Although there is a paucity of data on the long-term consequences of stroke on families in India, anecdotal evidence indicates a significant burden, particularly in rural areas, and in less educated families (Dalal, 2006).

In India, most people with stroke have recovery supported by families and their psychosocial problems could differ significantly from those experienced by families in developed countries. In a study of 152 carers (mean age 40·5), 85% had carer strain, with depression in 43% and anxiety in 47% (Raju et al., 2012). Organised support for patients and families is rare but crucial.

In IMPROVIS-ATION we will explore through interviews and discussion the potential for providing post-discharge support psychosocial support as well as hydration and swallowing. This could improve patient complications and reduce psychological distress for both patients and relatives, reducing morbidity and mortality.

During IMPROVISE and through discussions with our India-based collaborators, it was identified that a streamlined ethical approvals process could help to facilitate stroke research studies. Indian research ethics requires individual approval from each site, with Health Ministry Screening Committee (HMSC) approval finally conferred only after all site approvals are gained. Preliminary discussions with members of Indian Stroke Clinical Trial

For peer review only

Network (INSTRuCT) the Forum for Ethics Research Committees in India (FERCI) and the Indian Council for Medical Research (ICMR) suggest that there is the potential for a multi-centre ethics processes which would facilitate stroke and other multi-site clinical research studies in the future.

For peer review only

Protocol version: No. 1
Date: 29th June 2020

For peer review only - http://bmjopen.bmj.com/site/about/guidelines.xhtml

2. Aims of the research

Three exploratory work package studies (WPs) will establish the foundations to underpin future planned work to improve the delivery of acute and community stroke services that support stroke survivors.

Work package 1 (WP1) aim:

- To explore and gain an understanding of how stroke services are set up and operate in order to identify how to implement a hydration and swallowing care bundle.

Work package 2 (WP2) aims:

- To map the current resources available for hydration and swallowing problems, and psychosocial support following discharge.
- To identify the key components of potential interventions for the management of hydration and swallowing, and psychosocial support, post-discharge; and, explore potential options for interventions with clinical staff, stroke patients, and their relatives.

Work package 3 (WP3) aim:

- With key stakeholders, to map out the current ethics approval process, identify challenges, enablers and the feasibility of establishing a stream-lined multi-centre ethics approval process for stroke studies within the INSTRuCT network.

For peer review only - http://bmjopen.bmj.com/site/about/guidelines.xhtml

Figure 1. Study flow chart

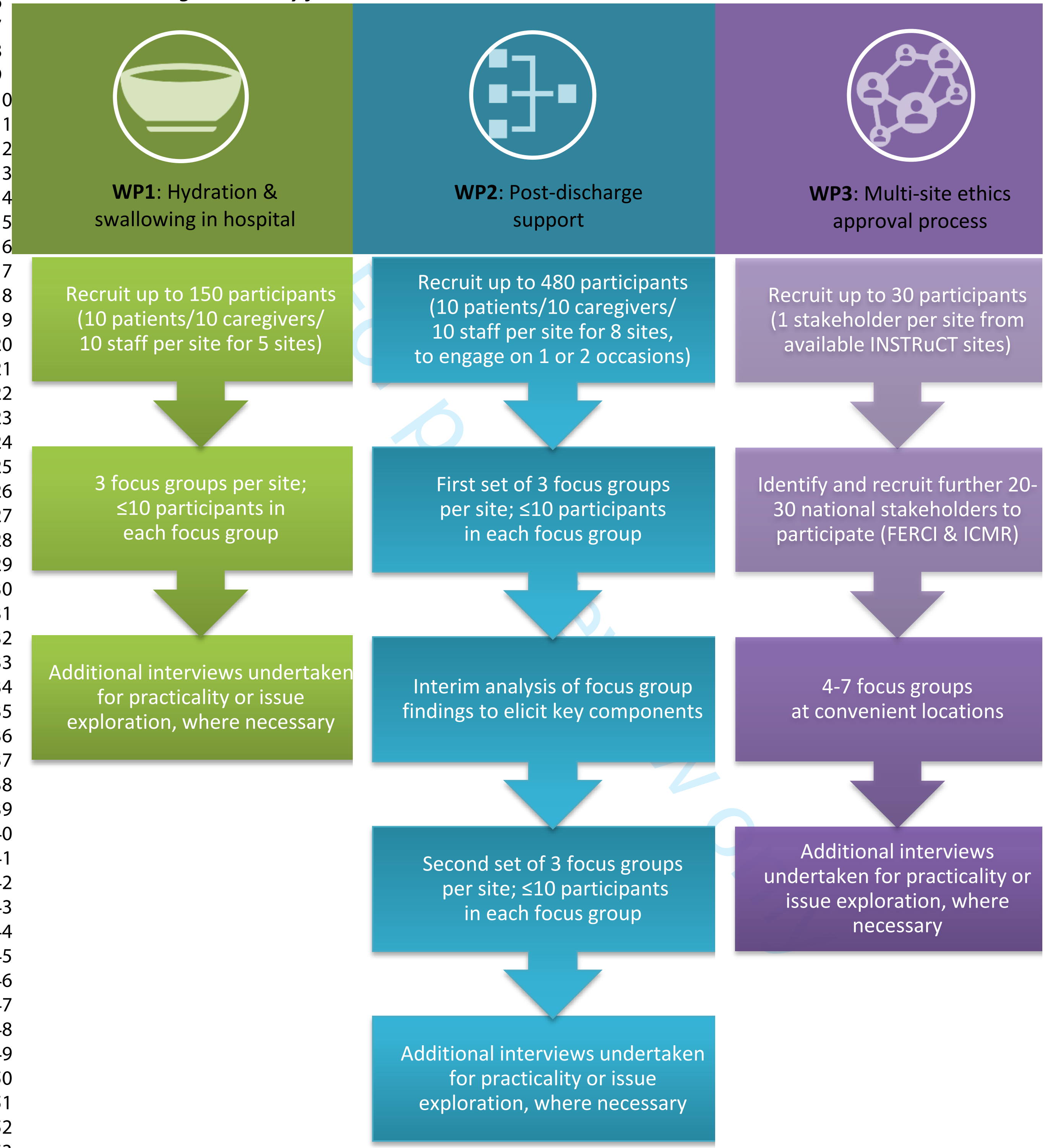

Protocol version: No. 1
Date: 29th June 2020

For peer review only - http://bmjopen.bmj.com/site/about/guidelines.xhtml

2.1 Overall participant timeline

Participants will be recruited between 1 November 2020 and 31 March 2021.

Figure 2. Study period timeline

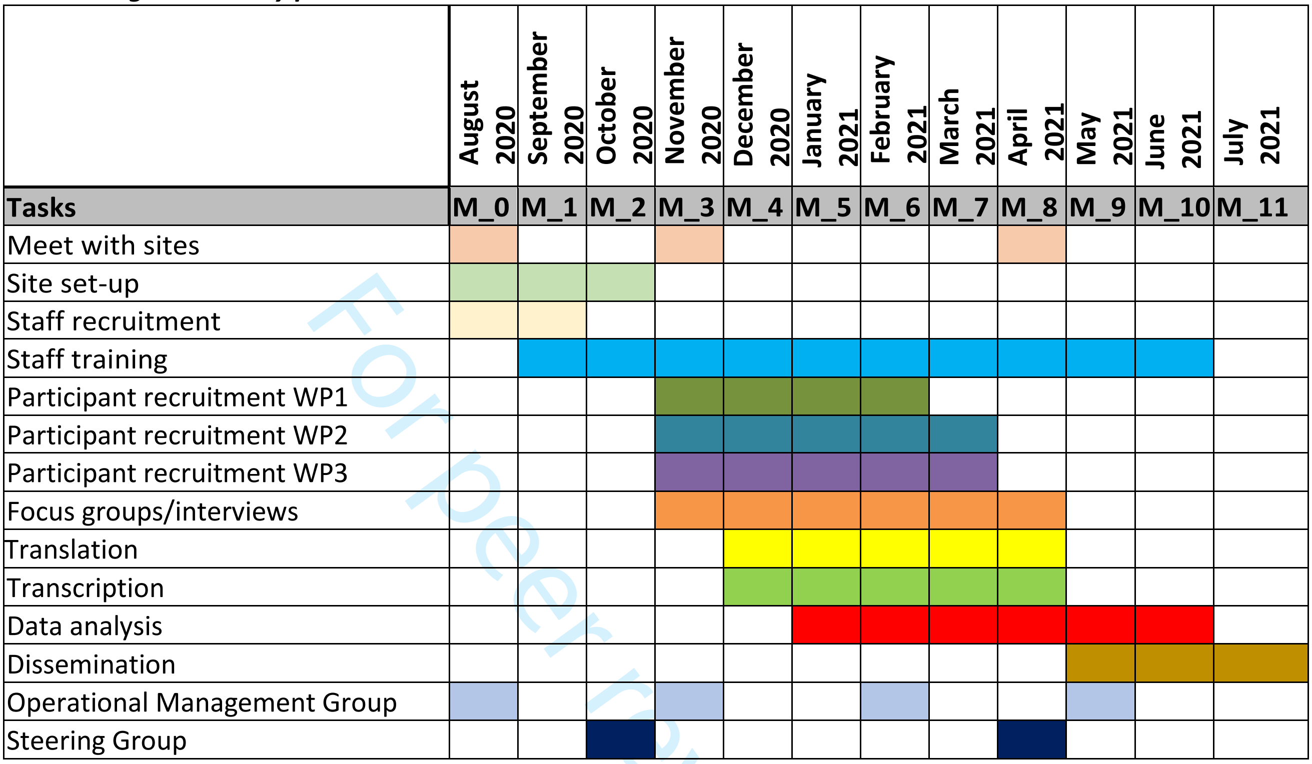

Protocol version: No. 1
Date: 29th June 2020

For peer review only - http://bmjopen.bmj.com/site/about/guidelines.xhtml

3. WP1 Protocol: Hydration and swallowing in hospital

3.1 WP1 aim and objectives

Aim: To explore and gain an understanding through interviews and focus groups of how stroke services are set up and operate, in order to identify how to implement a hydration and swallowing care bundle.

Objectives:

- To explore with stroke patients, families and staff, current screening, assessment and management for hydration and swallowing
- To understand challenges and solutions to the implementation of a hydration and swallowing management care bundle in hospital
- To understand regional differences
- To identify the key components for a potential implementation strategy of the hydration and swallowing management care bundle

3.2 WP1 study design

An exploratory study using qualitative research methods. Guided by a facilitator, focus groups will be conducted in local languages (with translators present to support participant engagement), with a note-taker and a secondary facilitator. Focus groups will explore topics including: experiences of access to and resources available for the assessment and management of hydration and swallowing; what people would want in relation to improving hydration and swallowing assessment and management within their locality and how this could be achieved. Focus groups will be conducted face-to-face or online using Zoom or similar technology. Semi-structured interviews will explore the same topics as in the focus groups; interviews may be undertaken to supplement focus group discussions, to follow up on focus group findings in more detail, and/or to ensure inclusivity where participant attendance at the focus groups is not feasible due to disabilities, clinical commitments and other practicalities. Interviews will also be conducted in local languages (with translators present, if required), and may be conducted either face-to-face or by telephone or using Zoom or similar technology (Topic guides Appendix 12 (staff members); Appendix 13 Patients/relatives).

3.3 WP1 study setting

This study will be conducted at five hospital sites across India:

For peer review only

1. All India Institute of Medical Sciences (AIIMS), Bhopal
2. Baptist Christian Hospital (BCH), Tezpur
3. Indira Gandhi Medical College & Hospital (IGMC), Shimla
4. National Institute of Mental Health and Neurosciences (NIMHANS), Bangalore
5. Zydus Hospital, Ahmedabad

3.4 WP1 participant eligibility

We will identify a convenience sample (using non-restrictive criteria) of patients and/or their relatives, and staff, with experience of stroke from a range of stroke services.

Subjects

We will recruit up to 150 participants Each site will host three focus groups, which include ≤10 participants in each staff, or patient and relative focus group (maximum individual commitment of 2-4 hours).

Patients

- Eligible for inclusion in the study are adult patients (aged ≥18 years) with experience of stroke or TIA.
- Excluded patients are those with no legally acceptable representative to consent

Relatives

- Eligible for inclusion in the study are adult (aged ≥18 years) relatives or caregivers for the person who has had the stroke or TIA.

Staff

Clinical staff (medical, nursing and therapy) will also be invited to participate.

3.5 WP1 consent procedure

The study will follow the consent procedure as listed in ICH GCP section 4.8 Informed Consent of Trial Subjects, Indian Council of Medical Research (ICMR), Ethical Guidelines for Biomedical Research on Human Participants and Indian Good Clinical Practices as laid down by Central Drugs Standard Control Organization (CDSCO).

For peer review only - http://bmjopen.bmj.com/site/about/guidelines.xhtml

Each patient/relative (Appendix 1 (WP1) and Appendix 5 (WP2)) and staff member (Appendix 3 (WP1)and Appendix 7 (WP2)) will be given a Participant Information Sheet (PIS) (which describes the study by a site RA or member of the clinical team. Participants agreeing to take part will be asked to provide written informed consent (Patients/relatives: Appendix 2 (WP1; Appendix 6 (WP2); Staff members: (Appendix 4 (WP1) and Appendix 8 (WP2)) before their focus group/interview; and, prior to any data being collected.

A legally acceptable representative may sign the consent form on behalf of an illiterate patient after verbally explaining the study details to them (illiterate patients to provide their thumb impression alongside signature of the legally acceptable representative). If neither a patient nor their legally acceptable representative are literate, an impartial witness (any person completely unrelated to the clinical study) may explain the details of the PIS to the patient and sign the consent form on behalf of the patient (who will provide their thumb impression alongside the signature of the impartial witness).

A copy of the consent form will be given to the participant. The original copy will be kept in the relevant Site File.

3.6 WP1 data collection

Demographic (age; sex; occupation) and stroke detail (type of stroke; severity of stroke [mild/moderate/severe]; experience of swallowing difficulties [current/recent/previous]; psychosocial difficulties (WP2 only); time since stroke; any disability [modified Rankin scale: MRS]) will be collected for all recruited participants from the patient and/or relative (Appendix 9). If this is not possible, this information will be recorded from the patient's medical records by a member of the clinical team.

Details of relationship to patient and age will be collected for recruited relatives in the consent form.

Job role, grade and place of work will be collected for all recruited staff members in the consent form.

3.7 WP1 outputs

- An understanding of current service provision and processes
- An understanding of barriers and facilitators from a range of perspectives

For peer review only

For peer review only

Protocol version: No. 1
Date: 29th June 2020

For peer review only - http://bmjopen.bmj.com/site/about/guidelines.xhtml

4. WP2 Protocol: Post-discharge support

4.1 WP2 aims and objectives

Aim: Through interviews and focus groups to:

- Map the current resources available for hydration and swallowing problems and psychosocial support following discharge
- Identify the key components of potential interventions for the management of hydration and swallowing, and psychosocial support post-discharge, and explore potential options for interventions with clinical staff, relatives of stroke patients, and stroke patients

Objectives:

- To explore patient pathways post-discharge and understand resources available for care
- To understand patients' and relatives' concerns, contextual challenges, and solutions
- To understand regional differences
- To identify key components of potential interventions

4.2 WP2 study design

An exploratory study using qualitative research methods. Guided by a facilitator, focus groups will be conducted in local languages (with translators present to support participant engagement), with a note-taker and a secondary facilitator. Focus groups will explore topics including: experiences of, access to and resources available for the on-going assessment and management of hydration and swallowing and post-discharge support; what people would want in relation to improving post-discharge support within their locality and how this could be achieved. Focus groups will be conducted face-to-face or online using Zoom or similar technology. Semi-structured interviews will explore the same topics as in the focus groups; interviews may be undertaken to supplement focus group discussions, to follow up on focus group findings in more detail, and/or to ensure inclusivity where participant attendance at the focus groups is not feasible due to disabilities, clinical commitments and other practicalities.

For peer review only

Interviews will also be conducted in local languages (with translators present, if required), and may be conducted either face-to-face or by telephone or using Zoom or similar technology. (Topic guides Appendix 14 (staff members); Appendix 15 Patients/relatives).

4.3 WP2 study setting

This study will be conducted at eight hospital sites in India, the five listed above and:

1. All India Institute of Medical Sciences (AIMS), New Delhi.
2. Christian Medical College (CMC), Ludhiana, Punjab.
3. Sree Chitra Tirunal Institute of Medical Sciences (SCTIMS), Trivandrum, Kerala.

Subjects

We will recruitment a maximum of 480 participants across the 8 sites. Each site will host a maximum of six focus groups, which include ≤10 participants (staff, or patient and relative focus group).

4.4 WP2 participant eligibility

We will identify a convenience sample (using non-restrictive criteria) of patients and/or their relatives, and staff, with experience of stroke from a range of stroke services.

Patients

- Eligible for inclusion in the study are adult patients (aged ≥18 years) discharged from hospital with experience of stroke.
- Excluded patients are those with no legally acceptable representative to consent

Relatives

- Eligible for inclusion in the study are adult (Aged ≥18 years) relatives or caregivers for the person who has had the stroke following discharge home from hospital.

Staff

Clinical staff (medical, nursing and therapy) will also be invited to participate.

For peer review only - http://bmjopen.bmj.com/site/about/guidelines.xhtml

4.5 WP2 participant recruitment

At each hospital site, the site Research Associates (RAs) and/or clinical team will invite patients, and any relatives required to support them, to take part in a focus group or interview; where possible these participants will reflect a range of characteristics, as applicable (age/sex/severity of stroke/experience of swallowing and/or psychosocial difficulties).

The site RAs will also invite a purposive sample of staff participants to represent a range of disciplines and grades of staff involved in the management of hydration and swallowing or post-discharge support.

Participants may choose to contribute to either one or two focus groups and/or interviews (maximum individual commitment of 2-4 hours).

4.6 WP2 consent procedure

As described in WP1.

4.7 WP2 data collection

As described in WP1.

4.8 WP2 outputs

- An understanding of current service provision and resources
- An understanding of the needs and preferences of patients and their relatives, and clinical staff, in relation to the management of hydration and swallowing, and psychosocial support following discharge from hospital
- Identification of the key components of interventions for supporting patients and their relatives following discharge from hospital

For peer review only - http://bmjopen.bmj.com/site/about/guidelines.xhtml

5. WP3 Protocol: Multi-site ethical approval process

5.1 WP3 aim and objectives

Aim: With key stakeholders, to map out the current ethics approval process, identify challenges, enablers and the feasibility of establishing a stream-lined multi-centre ethics approval process for stroke studies within the Indian Stroke Clinical Trial (INSTRuCT) network.

Objectives:

- To understand challenges
- To identify enhanced processes for ethical approval

5.2 WP3 study design

Design: an exploratory study using qualitative research methods.

Focus groups will be guided by a facilitator, conducted in local languages (with translators present to support participant engagement), with a note-taker and a secondary facilitator (if required). Focus groups will be conducted face-to-face at convenient locations or online using Zoom or similar technology. Focus group discussion topics will include: How the current ethics approval processes currently operates; what are the current challenges; are there any changes that the group like to see made to the ethics approval process and how might these be implemented.

Covering the same topics, semi-structured interviews may be undertaken to inform focus group discussions, to follow up on focus group findings in more detail, and/or to ensure inclusivity where participant attendance at focus groups is not feasible due restrictions on movement, other practicalities and clinical commitments. Interviews will also be conducted in local languages (with translators present to support participant engagement) and may be conducted either face-to-face or by telephone or video-conferencing technology (Stakeholder topic guide Appendix 16).

5.3 WP3 study setting

The INSTRuCT network relevant organisations, members of the Forum for Ethics Review Committees in India (FERCI) and the Indian Council of Medical Research (ICMR).

For peer review only - http://bmjopen.bmj.com/site/about/guidelines.xhtml

5.4 WP3 participant eligibility

Key stakeholders will be invited to participate in focus groups and interviews as follows:

- Employed by an appropriate INSTRuCT site, within either stroke or ethics unit
- From relevant organisations, such as the Forum for Ethics Review Committees in India (FERCI) and the Indian Council of Medical Research (ICMR)
- Actively involved in developing multi-centre research ethics processes though IMPROVIS-ATION

5.5 WP3 participant recruitment

We will recruitment a maximum of 60 participants (30 INSTRuCT network site stakeholders (one per site) and up to 30 FERCI and/or ICMR stakeholders). The programme manager will invite site stakeholders to take part, and organisational stakeholders (FERCI/ICMR) will self-nominate themselves to participate after initial approach through the INSTRuCT network.

Between 4-7 focus groups will be conducted at convenient locations, which include ≤10 participants in each focus group (any combination of available stakeholders; maximum individual commitment of 2-4 hours).

The findings from the focus groups and interviews will be synthesised and presented at a final discussion group to identify the key components of any changes and to agree next steps.

5.6 WP3 data collection

Job role, place of work and ethics committee experience information will be collected for all recruited key stakeholders in the consent form.

5.7 WP3 consent procedure

Each identified key stakeholder will be given a PIS describing the study (Appendix 10) by a member of the research team, issued via e-mail. Any key stakeholder agreeing to take part will be asked to provide written informed consent (Appendix 11) prior to their participation in a focus group or interview.

For peer review only - http://bmjopen.bmj.com/site/about/guidelines.xhtml

For peer review only

5.8 WP3 outputs

- An understanding of the current ethics approval processes
- Identification of potential challenges, enablers, and feasibility of developing a stream-lined multi-centre ethics approval process for stroke studies within the INSTRuCT network.

For peer review only

Protocol version: No. 1
Date: 29th June 2020

For peer review only - http://bmjopen.bmj.com/site/about/guidelines.xhtml

6. Overall operations across work packages

6.1 Analysis

Demographic data will be analysed descriptively. Focus groups/Interviews will be transcribed, and then analysed utilising NVivo (a qualitative data analysis computer software package) to support the analysis process. Framework will be used, this permits identification and cross-classification of variables through: identification of key concepts, mapping their range and diversity, interpreting patterns of association and investigating possible reasons. This would give a local service provider perspective on barriers/facilitators to implementing the potential pathways/reforms in the future, ensuring services fit needs. Discussion notes will also be collated and summarised.

6.2 Data management

Data will be treated and stored according to the General Data Protection Regulation (Information Commissioners Office 2018), the Data Protection Act (2018), Caldicott Principles (Caldicott 2013) and the National Institute for Health Research (NIHR) Good Clinical Practice (GCP) Standards.

Written consent forms will be stored in each hospital's Site File (WPs 1 and 2) and at CMC, the Co-ordinating Centre (WP3). All other data will be stored electronically in password-protected files. Members of the research team will have access to project files and data as appropriate to their role.

Participants will be assigned a participant number, this will be used to anonymise their data. Their anonymised data will be typed up into electronic format and stored in a secure OneDrive folder. Audio files from digital voice recorders will be translated, translations checked and transcribed. Once the transcription has been checked the audio file will be deleted. Anonymised transcriptions will be stored on the secure University of Central Lancashire network for five years following completion of the study in-line with the UCLan Data Protection Code of Practice.

Any individuals' names mentioned in the focus group/interview discussions will be removed from transcription. It will not be possible to recognise individuals from information in the completed data analysis or write up of final reports and publications, and any direct quotes used will remain anonymous.

For peer review only - http://bmjopen.bmj.com/site/about/guidelines.xhtml
For peer review only

6.3 Monitoring

The Project Co-ordinator from CMC and Research Programme Manager at the University of Central Lancashire will monitor training, recruitment and consent procedures.

Monitoring will facilitate confirmation of site adherence to this protocol and Good Clinical Practice (GCP) Guidelines, relevant local and regional ethical requirements, data accuracy and quality. The study may also be audited by external government or regulatory authorities.

In WPs 1 and 2, each site is responsible for maintaining a Site File containing a delegation log, a training log, and validity of consent log. In WP3, CMC will retain a Site File containing: a delegation log and validity of consent log.

Access to files will be made available by all participating sites for monitoring and audit purposes. On-site monitoring will be undertaken by the University of Central Lancashire in the form of (i) scheduled site visits during the study; or, (ii) triggered site visits in response to concerns relating to any study research processes.

6.4 Ethics and regulatory approval

The work packages, developed under this protocol, will be designed and implemented to comply with Ethical Principles for Medical Research Involving Human Patients (World Medical Association Declaration of Helsinki), Ethical Guidelines for Biomedical Research on Human Participants by Indian Council of Medical Research (ICMR), UK

Ethical and Research Governance approval will be obtained from individual sites' ethics committees and The University of Central Lancashire. Health Ministry Screening Committee (HMSC) approval will be sought from ICMR. If appropriate, the study will be registered on www.ctri.nic.in (Clinical Trials Registry-India (CTRI)) before study (patient-related) activities commence.

The CI is responsible for communicating any protocol amendments to the site PI. The site PI is responsible for ensuring that amendments are implemented, and that all documents replaced by 'new' versions are destroyed immediately. Site staff need to be informed of, and start using, new documentation as soon as local approvals are in place.

The site PI is responsible for reporting any protocol violations and routine reporting

For peer review only - http://bmjopen.bmj.com/site/about/guidelines.xhtml

outcomes, reporting to the CI and to their own ethical and governing bodies according to the requirements of the study protocol and written approval of the Ethics Committee.

Consent withdrawal

Participants may withdraw from the study without providing a reason. In these circumstances, the participant will be asked if we can retain and use their data. The PIS emphasises participation is voluntary and that people have the right to withdraw up until they take part in a focus group/discussion, or to the point where their data is anonymised after taking part in an interview and the data becomes fully anonymised once the data have been combined for analysis

6.5 Adverse events & safeguarding

Any adverse events directly resulting from the study will be reported to the site PI (who will inform the Programme Manager in the UK), in accordance with ICH Good Clinical Practices.

Any identified safeguarding concerns will be managed in accordance with the policies and procedures at each individual site.

6.6 Dissemination plan

A comprehensive dissemination plan will be developed with stakeholders, including outputs in clinical and academic peer-reviewed journals, conferences and social media outlets.

Study-generated data will be made available to other researchers for secondary research purposes, as agreed by the partnership and in accordance with open access requirements. This process will be managed and assured by the University of Central Lancashire, in accordance with institutional policy and procedures.

6.7 Study management

The study will be managed in accordance with the governance structure and processes in place for the NIHR Global Health Research Group (GHRG).

For peer review only - http://bmjopen.bmj.com/site/about/guidelines.xhtml

Professor Dame Caroline Watkins is the Director of the NIHR GHRG, with support from Deputy Directors: Professor Liz Lightbody, Deputy Director (Nursing Stroke), UCLAN; Professor Jeyaraj Pandian, Deputy Director (Neurology), Christian Medical College; Dr Pallab Maulik, Deputy Director (Psychiatry), The George Institute for Global Health. Members of the group have worked collaboratively to deliver a number of innovative large-scale multi-disciplinary studies in stroke including in the UK and India. Further details of the group are available at uclanglobalhealth.co.uk

The progress of the project will be overseen every three months by the Operational Management Group (OMG), which will ensure effective operational management of the project with reference to agreed work plans, milestones and key deliverables. The OMG consists of the Director, Deputy Directors, Work Package and site leads, and the Programme Manager.

The overall delivery of the programme will be overseen by the Steering Group, which provides oversight, advice and support to the project team. The Steering Group will make recommendations relating to project delivery to the funder and project team, undertake periodic reviews, and is responsible for the content of formal reports to the programme funder (NIHR). The group consists of a Chair, Directors, work package leads, representative of the funder, and other experts independent of the programme team. The Steering Group will meet regularly, approximately every 3-6 months depending on the level of activity in the programme.

7. References

Bray B, Ayis S, Campbell J, Hoffman A, Roughton M, Tyrrell PJ, Wolfe CDA, Rudd AG Associations between the organisation of stroke services, process of care, and mortality in England: prospective cohort study. BMJ. 2013, 346

Cadilhac DA, Andrew NE, Lannin NA, Middleton S, Levi CR, Dewey HM, Grabsch B, Faux S, Hill K, Grimley R, Wong A, Sabet A, Butler E, Bladin CF, Bates TR, Groot P, Castley H, Donnan GA, Anderson CS and Australian Stroke Clinical Registry C. Quality of Acute Care and Long-Term Quality of Life and Survival: The Australian Stroke Clinical Registry. Stroke. 2017;48:1026-1032.

Dalal PM. Burden of stroke: Indian perspective. International Journal of Stroke. 2006; 1:164-6

MacKay J, Mensah G. Atlas of Heart Disease and Stroke. World Health Organisation. 2004.

Pandian JD, Sudhan P. Stroke epidemiology and stroke care services in India. Journal of Stroke. 2013;15(3):128-134.

Raju RS, Kaur P and Pandian JD. Psychosocial problems, quality of life, and caregiver burden among stroke caregivers in India. International Journal of Stroke. 2012;7: 100-101. doi:10.1111/j.1747-4949.

Stroke Unit Trialists' Collaboration. Organised inpatient (stroke unit) care for stroke. Cochrane Database of Systematic Reviews. 2013, Issue 9. Art. No.: CD000197

The ATTEND Collaborative Group. Family-led rehabilitation after stroke in India (ATTEND): A randomised controlled trial. The Lancet, 2017; 390:10094. doi.org/10.1016/S0140-6736(17)31447-2.

The Stroke Association. State of the Nation. Stroke statistics. 2016

World Health Organisation. Global status report on non-communicable diseases. 2014. WHO Press, Switzerland.

For peer review only - http://bmjopen.bmj.com/site/about/guidelines.xhtml

COREQ (COnsolidated criteria for REporting Qualitative research) Checklist

A checklist of items that should be included in reports of qualitative research. You must report the page number in your manuscript where you consider each of the items listed in this checklist. If you have not included this information, either revise your manuscript accordingly before submitting or note N/A.

Topic	Item No.	Guide Questions/Description	Reported on Page No.
**Domain 1: Research team and reflexivity**			
Personal characteristics			
Interviewer/facilitator	1	Which author/s conducted the interview or focus group?	
Credentials	2	What were the researcher's credentials? E.g. PhD, MD	
Occupation	3	What was their occupation at the time of the study?	
Gender	4	Was the researcher male or female?	
Experience and training	5	What experience or training did the researcher have?	
Relationship with participants			
Relationship established	6	Was a relationship established prior to study commencement?	
Participant knowledge of the interviewer	7	What did the participants know about the researcher? e.g. personal goals, reasons for doing the research	
Interviewer characteristics	8	What characteristics were reported about the inter viewer/facilitator? e.g. Bias, assumptions, reasons and interests in the research topic	
**Domain 2: Study design**			
Theoretical framework			
Methodological orientation and Theory	9	What methodological orientation was stated to underpin the study? e.g. grounded theory, discourse analysis, ethnography, phenomenology, content analysis	
Participant selection			
Sampling	10	How were participants selected? e.g. purposive, convenience, consecutive, snowball	
Method of approach	11	How were participants approached? e.g. face-to-face, telephone, mail, email	
Sample size	12	How many participants were in the study?	
Non-participation	13	How many people refused to participate or dropped out? Reasons?	
Setting			
Setting of data collection	14	Where was the data collected? e.g. home, clinic, workplace	
Presence of non-participants	15	Was anyone else present besides the participants and researchers?	
Description of sample	16	What are the important characteristics of the sample? e.g. demographic data, date	
Data collection			
Interview guide	17	Were questions, prompts, guides provided by the authors? Was it pilot tested?	
Repeat interviews	18	Were repeat inter views carried out? If yes, how many?	
Audio/visual recording	19	Did the research use audio or visual recording to collect the data?	
Field notes	20	Were field notes made during and/or after the inter view or focus group?	
Duration	21	What was the duration of the inter views or focus group?	
Data saturation	22	Was data saturation discussed?	
Transcripts returned	23	Were transcripts returned to participants for comment and/or	

For peer review only

Topic	Item No.	Guide Questions/Description	Reported on Page No.
		correction?	
**Domain 3: analysis and findings**			
Data analysis			
Number of data coders	24	How many data coders coded the data?	
Description of the coding tree	25	Did authors provide a description of the coding tree?	
Derivation of themes	26	Were themes identified in advance or derived from the data?	
Software	27	What software, if applicable, was used to manage the data?	
Participant checking	28	Did participants provide feedback on the findings?	
Reporting			
Quotations presented	29	Were participant quotations presented to illustrate the themes/findings? Was each quotation identified? e.g. participant number	
Data and findings consistent	30	Was there consistency between the data presented and the findings?	
Clarity of major themes	31	Were major themes clearly presented in the findings?	
Clarity of minor themes	32	Is there a description of diverse cases or discussion of minor themes?	

Developed from: Tong A, Sainsbury P, Craig J. Consolidated criteria for reporting qualitative research (COREQ): a 32-item checklist for interviews and focus groups. *International Journal for Quality in Health Care*. 2007. Volume 19, Number 6: pp. 349 – 357

Once you have completed this checklist, please save a copy and upload it as part of your submission. DO NOT include this checklist as part of the main manuscript document. It must be uploaded as a separate file.

For peer review only

BMJ Open

Healthcare professionals' perspectives of the provision of, and challenges for, eating, drinking and psychological support post-stroke: findings from semi-structured interviews across India.

Journal:	*BMJ Open*
Manuscript ID	bmjopen-2022-069150.R2
Article Type:	Original research
Date Submitted by the Author:	09-Aug-2023

Complete List of Authors:	Jones, Stephanie ; University of Central Lancashire<br>Injety, Ranjit ; Christian Medical College and Hospital Ludhiana<br>Pandian, Jeyaraj ; Christian Medical College and Hospital Ludhiana, Neurology<br>Ratra, Sanjali; Christian Medical College and Hospital Ludhiana<br>Sylaja, PN; Sree Chitra Tirunal Institute for Medical Sciences and Technology, Neurology<br>Babu, Veena; Sree Chitra Tirunal Institute for Medical Sciences and Technology<br>Srivastava, Padma; All India Institute of Medical Sciences<br>Sharma, Sakshi; All India Institute of Medical Sciences<br>Sharma, Sudhir; Indira Gandhi Medical College<br>Webster, Jemin; Baptist Christian Hospital Tezpur<br>Koirala, Amrit; Baptist Christian Hospital Tezpur<br>Kaushal, Pawna; Indira Gandhi Medical College<br>Kulkarni, Girish Baburao; National Institute of Mental Health and Neuro Sciences<br>Dixit, Anand; National Institute of Mental Health and Neuro Sciences<br>Sharma, Arvind; Zydus Research Center<br>Prajapati, Jagruti; Zydus Research Center<br>Weldon, Jo; University of Central Lancashire<br>Kuroski, Jennifer A; University of Central Lancashire<br>Watkins, Caroline; University of Central Lancashire College of Health and Wellbeing, Faculty of Health and Wellbeing<br>Lightbody, Catherine; University of Central Lancashire, School of Nursing

**Primary Subject Heading**:	Global health
Secondary Subject Heading:	Health services research, Qualitative research
Keywords:	Stroke < NEUROLOGY, STROKE MEDICINE, QUALITATIVE RESEARCH, REHABILITATION MEDICINE

For peer review only - http://bmjopen.bmj.com/site/about/guidelines.xhtml

I, the Submitting Author has the right to grant and does grant on behalf of all authors of the Work (as defined in the below author licence), an exclusive licence and/or a non-exclusive licence for contributions from authors who are: i) UK Crown employees; ii) where BMJ has agreed a CC-BY licence shall apply, and/or iii) in accordance with the terms applicable for US Federal Government officers or employees acting as part of their official duties; on a worldwide, perpetual, irrevocable, royalty-free basis to BMJ Publishing Group Ltd ("BMJ") its licensees and where the relevant Journal is co-owned by BMJ to the co-owners of the Journal, to publish the Work in this journal and any other BMJ products and to exploit all rights, as set out in our licence.

The Submitting Author accepts and understands that any supply made under these terms is made by BMJ to the Submitting Author unless you are acting as an employee on behalf of your employer or a postgraduate student of an affiliated institution which is paying any applicable article publishing charge ("APC") for Open Access articles. Where the Submitting Author wishes to make the Work available on an Open Access basis (and intends to pay the relevant APC), the terms of reuse of such Open Access shall be governed by a Creative Commons licence – details of these licences and which Creative Commons licence will apply to this Work are set out in our licence referred to above.

Other than as permitted in any relevant BMJ Author's Self Archiving Policies, I confirm this Work has not been accepted for publication elsewhere, is not being considered for publication elsewhere and does not duplicate material already published. I confirm all authors consent to publication of this Work and authorise the granting of this licence.

For peer review only

Title: Healthcare professionals' perspectives of the provision of, and challenges for, eating, drinking and psychological support post-stroke: findings from semi-structured interviews across India.

Authors' names and affiliations

Stephanie P Jones[1], Ranjit J Injety[2], Jeyaraj D Pandian[2], Sanjali Ratra[2], P N Sylaja[3], Veena Babu[3], M V Padma Srivastava[4], Sakshi Sharma[4], Sudhir Sharma[5], Pawna Kaushal[5], Jemin J Webster[6], Amrit Koirala[6], Girish Baburao Kulkarni[7], Anand Dixit[7], Arvind Sharma[8], Jagruti Prajapati[8], Jo Catherine Weldon[1], Jennifer A Kuroski[1], Caroline Leigh Watkins[1], Catherine Elizabeth Lightbody[1] on behalf of the NIHR Global Health Research Group on IMPROVIng Stroke CarE in India (IMPROVISE) Collaboration.

[1]University of Central Lancashire, Preston, Lancashire

[2]Christian Medical College and Hospital, Neurology, Ludhiana, India,

[3]Sree Chitra Tirunal Institute for Medical Sciences and Technology, Neurology, Thiruvananthapuram, India,

[4]All India Institute of Medical Sciences, Neurology, New Delhi, India,

[5]IGMC Shimla, Neurology, Shimla, India,

[6]Baptist Christian Hospital, Department of Medicine, Tezpur, India,

[7]NIMHANS Bangalore, Neurology, Bangalore, India,

[8]Zydus Hospitals And Healthcare Research Pvt. Ltd, Neurology, Ahmedabad, India.

Named contact & address/affiliation
Dr Stephanie Jones
E-mail. Sjones10@uclan.ac.uk
Tel. +44 1772 895107
University of Central Lancashire, Preston, Lancashire, UK

Funder: National Institute for Health Research

Key words: Stroke, Post-discharge, Qualitative, India

Word count: 4092

For peer review only - http://bmjopen.bmj.com/site/about/guidelines.xhtml

Abstract

Aim: This qualitative study explores with health professionals the provision of, and challenges for, post-discharge stroke care, focussing on eating, drinking and psychological support across India.

Design: Qualitative semi-structured interviews.

Setting: Seven geographically diverse hospitals taking part in a Global Health Research Programme on Improving Stroke Care in India

Participants: A purposive sample of healthcare professionals with current experience of working with stroke patients.

Results: Interviews with 66 healthcare professionals (23 nurses (14 staff nurses; 7 senior nurse officers; 1 Intensive Care Unit nurse; 1 palliative care nurse)); 16 doctors (10 neurologists; 6 physicians); 10 physiotherapists; 5 speech and language therapists; 4 occupational therapists; 4 dieticians; 2 psychiatrists; and 2 social workers) resulted in three main themes: Integrated inpatient discharge care planning processes; Post-discharge patient and caregiver role and challenges; Patient and caregiver engagement post-discharge.

Conclusions: Discharge planning was integrated and customised, although resources were limited in some sites. Task-shifting compensated for a lack of specialists but was limited by staff education and training. Caregivers faced challenges in accessing and providing post-discharge care. Post-discharge care was mainly hospital-based, supported by tele-services, especially for rural populations. Further research is needed to understand post-discharge care provision and the needs of stroke survivors and their caregivers.

For peer review only - http://bmjopen.bmj.com/site/about/guidelines.xhtml

Strengths and limitations of this study

- We did not seek to interview HCPs based in the community as services varied between the included sites and were not stroke-specific.
- Due to the diversity of the included hospitals we were unable to make comparisons across services.
- It is acknowledged that one in every three patients with stroke do not access appropriate healthcare due to non-affordability, usage of alternative medicines, and difficulty in conveyance to hospital.
- Participants represented a broad range of stroke services; of which, over a third were nurses of varying seniority.
- Interview methods (telephonic or face-to-face) and interviews in local languages maximised participant engagement, and also enabled adherence to COVID-protocols, and inclusion of participants under COVID restrictions at the point of interview.

For peer review only - http://bmjopen.bmj.com/site/about/guidelines.xhtml

Background

Stroke is a significant global health problem and a major cause of mortality and morbidity in high income countries (HICs), and increasingly in low-middle income countries (LMICs) [1]. Seventy percent of strokes occur in LMICs; the subsequent disease burden is greater than in HICs [2]. Life expectancy in India has recently increased to over 60 years of age [3,4], leading to an increase in age-related, non-communicable diseases including stroke [5,6]. In India, the incidence of stroke ranges between 105 and 152/100,000 people per year [7]. Increasing incidence of stroke is observed among younger groups, with first ever strokes occurring around the age of 57 years [8-10], increasing families' burden.

Stroke survivors often experience many disabilities including hemiparesis, cognitive and communication problems, incontinence, dysphagia, and psychological trauma [11], having serious economic and social impacts [12]. In India, the majority of stroke survivors have disabilities, and the burden of long-term care often falls on family members [9]. This is particularly challenging when stroke survivors leave hospital with complex needs; requiring nasogastric tubes, catheters, and support with communication and psychological problems.

Stroke rehabilitation has been found to reduce mortality, decrease institutionalised care [13], and increase functional outcomes [14,15]. Despite the known benefits, India has limited resources to provide post-discharge stroke care [16]. Accessing rehabilitation services is a challenge for many stroke survivors in India. There are enduring barriers including long delays in accessing rehabilitation services, poor transport between home and hospital [17,18], and a lack of trained personnel [9]. There is a lack of data about post-discharge stroke services and the support available for patients and caregivers [14].

The resources for post-stroke rehabilitation, especially the therapy workforce and infrastructure, are very limited in many LMICs [19]. Beyond urban tertiary centres, rehabilitation services are scarcely available. There are <10 skilled rehabilitation practitioners per 1 million people in many LMICs, which does not allow specialization to treat a particular condition or disorders [20]. In India, it estimated that there are only 41,567 PTs, 3,100 OTs, and 1,700 SLTs, and these are geographically concentrated in urban areas leaving much of the nation without resources [15]. Given the scarcity of resources, the rehabilitation needs of the stroke survivors, especially in LMICs, remain largely unmet resulting in a growing need for cost-effective, multidisciplinary rehabilitation services, particularly post-discharge [21].

This qualitative study aimed to explore with health professionals the provision of, and challenges for, post-discharge stroke focussing on eating, drinking and psychological support across India.

For peer review only - http://bmjopen.bmj.com/site/about/guidelines.xhtml

Design

Qualitative semi-structured interviews.

Setting and subjects

Interviews were conducted with a purposive sample of HCPs (doctors, nurses, physiotherapists [PTs], occupational therapists [OTs], speech and language therapists [SLTs], dieticians, psychiatrists and social workers [SWs]) with current experience of working with stroke patients at seven geographically diverse hospitals that were a mix of private, government and teaching, and all taking part in a Global Health Research Programme on Improving Stroke Care in India (All India Institute of Medical Sciences, New Delhi; Baptist Christian Hospital, Tezpur; Christian Medical College, Ludhiana; Indira Gandhi Medical College & Hospital, Shimla; National Institute of Mental Health and Neurosciences, Bangalore; Sree Chitra Tirunal Institute of Medical Sciences, Trivandrum; Zydus Hospital, Ahmedabad) between July and December 2021.

Methods

Interviews explored HCPs' views of stroke patient management in preparation for discharge and post-discharge support for patients and caregivers, focussing on eating, drinking and psychological support.

The interview guide (Supplementary File 1) was informed by published research and developed collaboratively by the research team. While not formally piloted, the interview guide was revised following feedback from Research Associates (RAs) and Principal Investigators (PIs) at the included hospitals, to ensure that questions could be translated into regional languages (Assamese, Gujarati, Hindi and Malayalam). According to participant preference, interviews were conducted in English or regional languages, and took place either face-to-face or by telephone (dependent on current COVID-19 restrictions).

Data collection

All interviews were digitally recorded, translated (as required) and transcribed verbatim. All transcriptions were verified by RAs prior to analysis. Data collection continued until saturation, determined as no new information provided to enable the generation of additional codes from across the participants recruited.

For peer review only - http://bmjopen.bmj.com/site/about/guidelines.xhtml

Patient and Public Involvement

Patients and the public were involved in discussions to agree the priority areas that were the focus of post-discharge support interviews with healthcare professionals (eating, drinking and psychological support).

Analysis

Each transcript was read in full, and data explored using inductive coding and analysed using a reflexive thematic method. Thirty interviews were coded as a group and 36 by individual project members. Confirmative and contradictory results were discussed by the project team for consensus. Data were analysed thematically using NVivo software (version 1.5.2.). This manuscript was prepared in accordance with the Consolidated Criteria for Reporting Qualitative Research (COREQ) guidelines [22] (Supplementary File 2).

Reflexivity

All interviews were undertaken by RAs (AD, AK, JP, PK, SR, SS, VB) who were known by participants, but were not someone who they worked with clinically and lasted between 15 and 47 minutes. Interviews were conducted in Assamese (n=1), Gujarati (n=1), Hindi (n=12), Malayalam, (n=3) and English (n=51). All members of the project team were involved in data analysis. Dependability of the data may vary based on any staffing changes following the research undertaken, and all interviews took place during the COVID-19 pandemic. Interviews were undertaken with participants from seven hospitals across geographically diverse areas. To support the analysis, a supplementary mapping exercise was undertaken to identify hospital and community-based services. The findings from the mapping activity enabled cross-validation of the interview findings.

Ethical considerations

Ethics approval (REF: 2020-9715) was granted by the Health Ministry's Screening Committee (HMSC), India and each hospital's ethics committee. The study conforms to ethical standards of the Indian Council of Medical Research Guidelines [23]. In the UK, ethical approval was granted by the Science, Technology, Engineering and Medicine Ethics Committee, University of Central Lancashire (STEM 939). Ethical approval was granted for both written and/or verbal consent, which was obtained from all participants prior to any interviews.

For peer review only - http://bmjopen.bmj.com/site/about/guidelines.xhtml

Results

Sixty-six HCPs participated, 39 (59%) female, representing a range of health professionals (Table 1). Data saturation was not reached until 66 interviews had been completed and this likely reflects the diversity of the participants, hospital type, hospital settings and patient populations served.

Table 1: Participant demographics

Healthcare Professionals	N (%)
**Nurses**	**23 (35%)**
Staff Nurses	14 (21%)
Senior nurse officers	7 (11%)
Intensive care nurse	1 (1.5%)
Palliative care nurse	1 (1.5%)
**Doctors**	**16 (24%)**
Neurologists	10 (15%)
Physicians	6 (9%)
Physiotherapists	10 (15%)
Speech and language therapists	5 (8%)
Occupational therapists	4 (6%)
Dieticians	4 (6%)
Psychiatrists	2 (3%)
Social workers	2 (3%)

Three themes were identified:

1. Integrated Inpatient Discharge Care Planning Processes (5 sub-themes):
    - 1.1. Healthcare System and Resources
    - 1.2. Staff Identification, and Expectations of Caregivers
    - 1.3. Task-shifting
    - 1.4. Patient and Caregiver Training
    - 1.5. Education and Support in Preparation for Discharge
2. Post-Discharge Patient and Caregiver Roles and Challenges (3 sub-themes):
    - 2.1. Patient and Caregiver Characteristics
    - 2.2. Caregiver Roles
    - 2.3. Caregiver Challenges
3. Patient and Caregiver Engagement post-discharge (2 sub-themes):
    - 3.1. Post-Discharge Support - Hospital

For peer review only - http://bmjopen.bmj.com/site/about/guidelines.xhtml

For peer review only

3.2. Post-Discharge Support - Community-based

Theme 1: Integrated Discharge Care Planning Processes

Discharge planning processes were integrated throughout the patient pathway, beginning soon after admission although varied across sites. Discharge planning was frequently dependent on the availability of specialist staff, resources and facilities, and often resulted in task-shifting (defined as the rational redistribution of tasks among health workforce teams) due to a lack of staff availability/expertise. Participants reported that HCPs provided patients and caregivers with tailored, targeted education, training, and support throughout hospitalisation.

Sub-theme 1.1. Healthcare System and Resources

All sites had localised, inpatient discharge planning processes or protocols in place, but this was largely undocumented.

"We have discharge processes that the team work to but we don't have any written protocol but we are observing the patient from the admission." (01-05)

Staff identified that existing post-discharge management systems could be improved by formal documentation to facilitate a more consistent and comprehensive approach.

"I really feel like there is dire need of formulating a kind of discharge protocol for the patient." (03-02)

Sub-theme 1.2. Staff Identification, and Expectations of Caregivers

Identified by the clinical team, caregivers would most often be the spouse, another immediate or extended family member.

"We will ask about the immediate relatives, either a wife or husband, brother or sister, son or daughter. We also identify the immediate family or caregiver." (06-04)

Training took place with patients and caregivers expecting that caregivers would primarily support and provide post-discharge care.

"*They need to know how to give physiotherapy, occupational therapy, mental support, how to give food, speech related, so, they need to be a doctor, they need to be therapist, they need to be a nurse… If the caregiver is not up to the mark, the rehabilitation fails.*" (06-01)

For peer review only - http://bmjopen.bmj.com/site/about/guidelines.xhtml

On admission, an individualised approach was used to identify the most appropriate caregiver(s) for tailored training and support.

"It depends on the status of the patient according to the medical and surgical condition of patient… We educate the relatives and patients as well as possible." (07-03)

In addition to, or in the absence of, family caregivers, some patients employed the services of professional caregivers.

"Some patients have the capacity of paying, so they hire the physiotherapists, and they call them at their home…Some patients cannot pay but they need physiotherapy." (04-04)

Sub-theme 1.3. Task-shifting

Whilst some sites had well-resourced stroke teams, there was a shortage of dieticians, SWs and therapists (PTs, OTs, SLTs, psychologists) at other sites, where existing staff, predominantly nurses, provided information and support.

"At the time of discharge being a nurse, as we do not have speech and language therapist, then we [nurses] try to put patients in the upright position and ask them to speak as much as they can." (04-02)

"*We are at such a place where we do not get any psychologists, that thing [psychological problems] we [nurses] have to manage ourselves because we are very close to the patients*." (04-04)

Staff often described task-shifting as integral to their professional roles and responsibilities; however, the majority had not undertaken any formal training and had developed knowledge and skills largely through experiential learning.

"No, we didn't get any specific training for this [providing dietary advice]. Whatever we have studied during our nursing training, learned from doctors, and from the internet, on that basis we provide this information." (04-01)

Sub-theme 1.4. Patient and Caregiver Training

Patient and caregiver training usually began at admission and continued until discharge, with staff encouraging caregivers to observe all aspects of patient care, as required, including the preparation and administration of medication, positioning, changing dressings, feeding, the management of nasogastric (NG) tubes, maintaining hygiene, and rehabilitation exercises.

For peer review only - http://bmjopen.bmj.com/site/about/guidelines.xhtml

"We make sure the person who will be taking care of the patient at home is there from the beginning, so that they are very much part of all the processes that happen in the ward: changing dressings, changing position, and feeding and all those things." (02-06)

Staff felt that patients and caregivers should be prepared both mentally and physically for discharge but shared few examples of any formal psychological support or preparation.

"We tell them, start observing from now, because when you go home, you have to do it by yourself. So, they also get ready mentally and physically. (02-02)

Sub-theme 1.5. Education and Support in Preparation for Discharge

To prepare the patient and caregiver for discharge, training delivered by HCPs was predominantly provided through verbal instruction and explanation.

"When the relatives are in the hospital, we teach them verbally and some of the relatives they themselves write down [instructions]." (02-01)

Training was often supplemented by the provision of information, in a variety of formats including leaflets, videos, written instructions, diet charts, and discharge summaries. Some PTs provided pre-recorded videos of exercises, available through websites such as YouTube and/or recorded patients undertaking exercises.

"But then we do use videos like I said, sometimes pictures of exercises, but it's not uniform for all. (03-05)

Whilst training took place throughout patients' hospitalisation, education and information was largely provided within 24-48 hours of discharge. Leaflets were available in some sites, in most local languages, covering a range of topics to support the caregiver. In other sites discharge summaries and diet charts were the only source of written information and instruction.

"*In our stroke unit, we have made pamphlets regarding safe swallow techniques and how to feed the patient."* (06-02)

Accessible written information in appropriate languages was viewed as a valuable source of information, enabling information to be passed from the patient or primary caregiver in hospital to the main caregiver at home.

For peer review only - http://bmjopen.bmj.com/site/about/guidelines.xhtml

"We have pamphlets which are given to the patient depending on the language of their preference… that helps in dispersing information further because the caregiver who is there with the patient in the hospital may not be the same caregiver who is there at home." (01-04)

2. Post-Discharge Patient and Caregiver Roles and Challenges (3 sub-themes):

The patient's stroke and patients'/caregivers' levels of literacy were viewed as important factors in the subsequent 'successes' of post-discharge rehabilitation. HCPs identified multiple challenges in providing education and training, and specific challenges for the patients and caregivers themselves.

Sub-theme 2.1: Patient and Caregiver Characteristics

Female, family members were often the preferred caregivers for both male and female patients.

"W*e prefer training the female relative because the boys or males they usually go out for work.*" (02-01)

Stroke severity, post-stroke complications, cognitive and communication problems influenced the complexity and challenges of education and training for patients and their caregivers.

"*Some patients having pressure sores, second or third degree, then it will be really difficult to handle the wound, the relatives need more information about how to care for the wound, how to reduce the severity, when to change dressings, and how much time between changing the patient's position.*" (07-02)

Patients' and caregivers' educational attainments were viewed as important in effective training delivery. Patients and caregivers from rural locations often had lower levels of literacy that impacted their ability to understand, and to pay for post-discharge care needed.

"Those who come from village they are not educated and are daily wage workers who are below the poverty-line… they really cannot understand the importance of [rehabilitation] exercises." (02-03)

Sub-theme: 2.2. Caregiver Roles

Participants described post-discharge stroke care as largely the role of home-based caregivers (mainly family members) who had responsibility for a range of tasks, including rehabilitation, the prevention of complications and subsequent stroke, and the provision of psychosocial support. Caregivers supported activities of daily living such as maintaining hygiene, food preparation tailored to the patient's swallowing ability and dietary requirements (e.g. diabetes, low salt, lower calorie); the preparation and administration of medication; rehabilitation (recommended by PT or aimed at improving speech and/or

For peer review only - http://bmjopen.bmj.com/site/about/guidelines.xhtml

cognition); facilitating follow-up appointments; seeking help in an emergency; and supporting the patient's psychological/emotional needs.

"They have to do the medication on time. They have to do the proper positioning, proper personal hygiene to the patient, and then they have to maintain the proper diet to the patient, to give nourishment to the patients. The entire role in the hospital, that has to be continued in the home also." (01-06)

Sub-theme 2.3. Caregiver Challenges

Hospital staff provided support and education to an identified caregiver in hospital in preparation for discharge, although these trained caregivers were not always the primary caregiver at home.

"*Sometimes in rural areas women do not come when their husband is admitted in the hospital and the caretaker is different, so all these things happen, and I feel very bad.*" (01-10)

Staff reported that caregivers encountered a multitude of challenges including: financial constraints in healthcare expenses and loss of income; a lack of time to provide care around work commitments; varying levels of literacy, ability to comprehend, and to adhere to instructions; difficulties transporting the patient to hospital appointments and so resorting to nearer sources of help (including from local healthcare centres (with or without trained professionals), informal help (pharmacists), alternative medicine (AUYSH) and unqualified professionals (faith-healers, quacks); and a dearth of community-based services. Challenges were particularly marked among rural populations.

"*One of the negative aspects of patients being in rural areas is that most caregivers go out to work in daytime... They are unable to give enough time for patient care and exercise.*" (02-03)

Theme 3: Patient and Caregiver Engagement post-discharge

Provided at discharge, follow-up appointments were individualised and based on the patient's level of disability, co-morbidities and available post-discharge support. In some sites, dedicated stroke helplines, Stroke Unit contact details, or individual staff members' phone numbers were provided.

Sub-theme 3.1. Post-Discharge Support – Hospital

Staff reported that it was common for patients to have multiple follow-up appointments with their doctor, who was able to provide advice, treatment and referral to other services, as needed. Follow-up appointments usually took place at one-month post-discharge but varied according to patient need.

For peer review only - http://bmjopen.bmj.com/site/about/guidelines.xhtml

However, some patients had challenges in attending follow-up appointments, mainly due to the distance to the hospital from their home and travel costs.

"The patient comes after one month or if the patient is outside of the area, he may come after two months, three months. That is up to the patient. That is up to their condition. That is up to their circumstances." (01-01)

For patients who were asked to, and who were able to, attend follow-up, referral to specialist support was made available if needed or if this was part of on-going treatment.

"Once they come to come back for follow-up in the OPD they are sent to physiotherapy OPD for review. (02-03)

Tele-support was variable in availability and purpose. Staff reported that patients and caregivers were more likely to contact the hospital post-discharge when the patient had complex health needs (from their stroke and/or co-morbidities). The most common reasons for seeking help through teleservices were problems with NG tubes (feeding and blockages); advice about preparing food in different consistencies and condition specific diets (low sugar, low salt); questions about medication (dosage, frequency and side-effects); catheter care; monitoring or changes in blood pressure; clarifications about PT-based exercises; and, psychological problems (including anxiety around recurrent stroke and the impact of stroke on life expectancy).

"The patients are getting discharged, we give them our number. So, at this moment, we are trying to attend to them as much as possible with the phone. So, if they have any problem for example, if there is a catheter problem, or there is NG change we try to solve this through the phone." (02-05)

Some hospitals were able to refer patients with ongoing rehabilitation needs to rehabilitation clinics and networks; and where the patient lived some distance from the hospital to local healthcare facilities.

"There are three options. One is the inpatient programme, once they are medically stable. The second is to get admitted under a rehabilitation package. We have 7, 10 and 30-day packages where they stay in hospital and get rehabilitation. The next option is we have guest houses outside [the hospital] and they can stay there at very minimal prices and they then come on an OPD basis to the physiotherapist. The last option is our network of physios visit them at home or we send them videos through WhatsApp." (03-05)

For peer review only - http://bmjopen.bmj.com/site/about/guidelines.xhtml

For peer review only

At some hospitals there were also post-discharge outreach facilities (including palliative care and community nurses), but at others there were no such resources.

"We have palliative care nurses who do a lot of home visits, twice weekly, if there is any patient who is bedridden and fully dependent, those patients, we connect them to the palliative care nurses. Palliative care nurses visit them at home and change NG tubes, catheters, dressings and many more of those things." (02-01)

"We arrange a particular community level therapist, and we have that contact number too, so they can simply contact us regarding the patient's issues." (06-06)

Sub-theme: 3.2. Post-Discharge Support – Community-based

Outside of hospital-based care, post-discharge support services (such as counselling/psychological support and physiotherapy) were not stroke-specific and were considered by staff to deliver little additional benefit. Broader post-discharge support (outside hospital) was reportedly lacking in availability.

"There are some people who get people to massage [by private physiotherapists] and all the therapy techniques that were taught they will not use, they will use their own technique and do oil massage. So, it doesn't strengthen them, a weakness is there." (02-02)

Patients and caregivers, particularly in rural areas were often reported to have accessed alternative medicine, unqualified 'Doctors' or traditional faith healers post-discharge.

"The community will support more after discharge, they usually do so at the time of rituals. Pujari [priest] will advise many things that are advantageous or disadvantageous." (05-01)

Discussion

To our knowledge, this is the first qualitative study to explore post-discharge stroke care with HCPs providing stroke services across India. This study identified a number of strengths including comprehensive individualised treatment planning, task-shifting where there was a lack of specialists, comprehensive patient and caregiver training, and post-discharge care facilitated by hospital, and community-based services, where available. However, there were also some significant challenges.

Inpatient discharge care-planning processes usually began soon after admission but were often not formally documented, a process which may have facilitated a more consistent and comprehensive

For peer review only - http://bmjopen.bmj.com/site/about/guidelines.xhtml

approach. The provision of tailored and individualised information, education and training for patients and caregivers was often dependant on staff and resource availability, which commonly resulted in task-shifting. Task-shifting describes a situation where a task is transferred to a HCP with a different or lower level of education and training, or to a person specifically trained to perform limited tasks [24]. As not all hospitals in the study had comprehensive MDTs, with reported shortages of dieticians, SWs and therapy staff, in these instances, nurses would often take on the roles of other HCPs despite limited formal training. Whilst task-shifting often enables a more efficient use of available human resources and can constitute a robust strategy to provide stroke care [25] there is currently no documented evidence of stroke-specific task-shifting training for non-neurologist HCPs, with HCPs reporting that they largely developed knowledge and skills through informal or experiential learning.

Caregivers, often female family members, were responsible for all aspects of care and activities of daily living post-discharge, results echoed in previous research [26]. Their ability to deliver required healthcare and psychosocial support was often dependent on access to services (geographically and financially), and the availability of other family members generating income in their (or the stroke survivors) absence. Congruent with other reports, HCPs identified challenges in training and education, and consequently difficulties for caregivers when stroke survivors had more severe strokes, requiring complex care [27]. Whilst caregivers may have experienced increased demands, and in some cases limited preparedness for their new roles, unlike research from developed countries [28], this was viewed by HCPs to be a necessary responsibility rather than a burden.

As post-discharge care is frequently the responsibility of family members in LMICs [29], the preparation of caregivers was a vital aspect of in-hospital discharge planning. Training was usually delivered verbally, sometimes supported by alternative formats provided in local languages to enhance accessibility, comprehension and adherence. However, patient and caregiver characteristics (demographic, socio-economic, stroke severity) and social roles (employment status, family structure and dynamics), all had an impact on the subsequent accessibility, availability and provision of post-discharge care. Caregiver literacy was viewed as an importance aspect of successful post-discharge care. Previous research has demonstrated that low functional literacy may contribute to poor health status through a reduced ability to use written instructions and advice, and reduced adherence to medication and rehabilitation regimens [30]. Low literacy levels were viewed as a particular challenge in rural areas.

Post-discharge support provided by hospitals included multiple follow-up appointments, the frequency and number determined by the patient's condition. Post-discharge care included further service referrals (to rehabilitation clinics, local healthcare centres or post-discharge outreach facilities where available). However, post-discharge support beyond hospital settings was lacking in the community, as a result

For peer review only - http://bmjopen.bmj.com/site/about/guidelines.xhtml

alternative medicine, unlicensed practitioners and faith-healers were often sought, particularly among rural populations. In addition, other non stroke-specific rehabilitation services with limited patient benefit were relied upon. Complementary and alternative medicine (CAM) approaches for post-stroke recovery are frequently accessed in India and often include ayurvedic massage, herbal medicine, marma therapy, reiki therapy, homeopathy, intravenous fluids, and opium [31].

Further data and research are needed to understand the support available and needs of stroke survivors and caregivers, to inform the development of innovative post-stroke interventions that are accessible, patient-centered, and culturally sensitive [18].

This study has a number of limitations. Firstly, the study did not seek to interview HCPs based in the community as these services varied between the included sites and were not stroke-specific. Due to the diversity of the included hospitals we were unable to make comparisons across services. Hospital staff broadly lacked awareness of the spectrum of stroke care services available in-hospital (outside of their own profession) and in the community. Finally, it is acknowledged that one in every 3 patients with stroke do not access appropriate healthcare due to non-affordability, usage of alternative medicines, and difficulty in conveyance to hospital [9].

We have also identified several strengths: Participants represented a broad range of stroke services; of which, over a third were nurses of varying seniority. Interviews were conducted in local languages to maximise engagement and by providing participants with a choice of interview method (telephonic or face-to-face), this enabled adherence to COVID-protocols, and supported inclusion of participants under COVID restrictions at the point of interview.

Conclusion

Discharge planning was integrated and customised but in some sites there were limited resources.

Task-shifting among HCPs compensated for a lack of specialists, but limitations were identified in staff education and training. Caregivers faced many challenges in both accessing and providing post-discharge care. Post-discharge care was mainly hospital-based, supported by tele-services, especially for rural populations. Further research is needed to gain an understanding of post-discharge care provision and the needs of stroke survivors and their caregivers.

Funding statement

This research was funded by the National Institute for Health Research (NIHR) [Global Health Research Group on Improving Stroke Care, University of Central Lancashire (16/137/16)] using UK aid from the UK Government to support global health research. The views expressed in this publication are those of the author(s) and not necessarily those of the NIHR or the UK government.

For peer review only - http://bmjopen.bmj.com/site/about/guidelines.xhtml

Competing interests statement

There are no conflicts of interest to declare.

Data availability statement

No additional data available.

Author contributions

Authors CEL, PMV, PJD, SPN and CLW contributed to the study conception and study design. Data collection was undertaken by PK, SR, SS, AK, AD, JP, VB . Analysis was performed by SJ, RI, JW, WK, PK, SR, SS, AK, AD, JP, CEL, PMV, PJD, SPN, CLW, JW, AS, SS, GK. The first draft of the manuscript was written by SJ, RI and JW. All authors commented on previous versions of the manuscript. All authors read and approved the final manuscript.

Acknowledgements

We would like to thank the health professionals who took the time to take part in this study. We would also like to thank the members of the NIHR Global Health Research Group on IMPROVIng Stroke CarE in India (IMPROVISE) Collaboration.

References

1. Feigin VL, Forouzanfar MH, Krishnamurthi R, et al. Global and regional burden of stroke during 1990-2010: findings from the Global Burden of Disease Study 2010. *Lancet*. 2014;383(9913):245-254. doi:10.1016/s0140-6736(13)61953-4

2. Roth GA, Abate D, Abate KH, et al. Global, regional, and national age-sex-specific mortality for 282 causes of death in 195 countries and territories, 1980–2017: a systematic analysis for the Global Burden of Disease Study 2017. *The Lancet*. 2018;392(10159):1736-1788. doi:10.1016/S0140-6736(18)32203-7

3. Yamunadevi A, Sulaja S. Old Age Mortality in India –An Exploration from Life Expectancy at Age 60. *International Journal of Asian Social Science*. 2016;6(12):698-704. doi:10.18488/journal.1/2016.6.12/1.12.698.704

4. Horton R, Das P. Indian health: the path from crisis to progress. *The Lancet*. Published online 2011. doi:10.1016/S0140-6736(10)62179-4

5. Dalal P, Bhattacharjee M, Vairale J, Bhat P. UN millennium development goals: Can we halt the stroke epidemic in India? *Annals of Indian Academy of Neurology*. 2007;10(3):130. doi:10.4103/0972-2327.34791

6. Naik K. Challenges in delivering stroke care in India. *Indian j health sci*. 2016;9(3):245. doi:10.4103/2349-5006.196342

For peer review only - http://bmjopen.bmj.com/site/about/guidelines.xhtml

7. Jones SP, Baqai K, Clegg A, et al. Stroke in India: A systematic review of the incidence, prevalence, and case fatality. *International Journal of Stroke*. 2022;17(2):132-140. doi:10.1177/17474930211027834

8. Pandian JD, Jaison A, Deepak SS, et al. Public awareness of warning symptoms, risk factors, and treatment of stroke in northwest India. *Stroke*. 2005;36(3):644-648. doi:10.1161/01.STR.0000154876.08468.a0

9. Pandian JD, Sudhan P. Stroke epidemiology and stroke care services in India. *J Stroke*. 2013;15(3):128-134. doi:10.5853/jos.2013.15.3.128

10. Tripathi M, Padhy UP, Vibha D, et al. Predictors of refractory epilepsy in north India: a case-control study. *Seizure*. 2011;20(10):779-783. doi:10.1016/j.seizure.2011.07.010

11. Lloyd J, Pinto AM, Nair S, Tarey S. A Qualitative Study on Palliative Needs of Stroke Patients in an Indian Tertiary Care Setting - Doctors' Perspective. *Indian J Palliat Care*. 2019;25(1):84-91. doi:10.4103/IJPC.IJPC_161_18

12. Walsh ME, Galvin R, Loughnane C, Macey C, Horgan NF. Community re-integration and long-term need in the first five years after stroke: results from a national survey. *Disability and Rehabilitation*. 2015;37(20):1834-1838. doi:10.3109/09638288.2014.981302

13. Langhorne P, Ramachandra S, Stroke Unit Trialists' Collaboration. Organised inpatient (stroke unit) care for stroke: network meta-analysis. *Cochrane Database Syst Rev*. 2020;4:CD000197. doi:10.1002/14651858.CD000197.pub4

14. Lindley RI, Anderson CS, Billot L, et al. Family-led rehabilitation after stroke in India (ATTEND): a randomised controlled trial. *The Lancet*. 2017;390(10094):588-599. doi:10.1016/S0140-6736(17)31447-2

15. Pandian JD, Gandhi DBC, Lindley RI, Bettger JP. Informal Caregiving. *Stroke*. 2016;47(12):3057-3062. doi:10.1161/STROKEAHA.116.013701

16. Sylaja PN, Srivastava MVP, Shah S, et al. The SARS-CoV-2/COVID-19 pandemic and challenges in stroke care in India. *Ann N Y Acad Sci*. 2020;1473(1):3-10. doi:10.1111/nyas.14379

17. Banerjee TK, Das SK. Fifty years of stroke researches in India. *Ann Indian Acad Neurol*. 2016;19(1):1-8. doi:10.4103/0972-2327.168631

18. Kamalakannan S, Gudlavalleti Venkata M, Prost A, et al. Rehabilitation Needs of Stroke Survivors After Discharge From Hospital in India. *Arch Phys Med Rehabil*. 2016;97(9):1526-1532.e9. doi:10.1016/j.apmr.2016.02.008

19. World Health Organization, World Heart Federation, World Stroke Organization. Global atlas on cardiovascular disease prevention and control. *Global atlas on cardiovascular disease prevention and control*. Published online 2011:155-155.

20. World Health Organization. *Global Diffusion of EHealth: Making Universal Health Coverage Achievable: Report of the Third Global Survey on EHealth*. World Health Organization; 2017.

21. Bonita R, Beaglehole R. Stroke prevention in poor countries: time for action. *Stroke*. 2007;38(11):2871-2872. doi:10.1161/STROKEAHA.107.504589

For peer review only - http://bmjopen.bmj.com/site/about/guidelines.xhtml

22. Tong A, Sainsbury P, & Craig J. Consolidated criteria for reporting qualitative research (COREQ): A 32-item checklist for interviews and focus groups. *International Journal for Quality in Health Care*, 2017;19(6), 349–357. https://doi.org/10.1093/intqhc/ mzm042

23. Guidelines | Indian Council of Medical Research | Government of India. Indian Council of Medical Research. Published 2017. Accessed June 29, 2022. https://main.icmr.nic.in/content/guidelines-0

24. Joshi R, Alim M, Kengne AP, et al. Task shifting for non-communicable disease management in low and middle income countries--a systematic review. *PLoS One*. 2014;9(8):e103754. doi:10.1371/journal.pone.0103754

25. Lindsay P, Furie KL, Davis SM, Donnan GA, Norrving B. World Stroke Organization global stroke services guidelines and action plan. *International journal of stroke : official journal of the International Stroke Society*. 2014;9 Suppl A100. doi:10.1111/ijs.12371

26. Mandowara B, Patel AN, Amin AA, Phatak A, Desai S. Burden Faced by Caregivers of Stroke Patients Who Attend Rural-based Medical Teaching Hospital in Western India. *Ann Indian Acad Neurol*. 2020;23(1):38-43. doi:10.4103/aian.AIAN_406_18

27. Rigby H, Gubitz G, Phillips S. A Systematic Review of Caregiver Burden following Stroke. *International Journal of Stroke*. 2009;4(4):285-292. doi:10.1111/j.1747-4949.2009.00289.x

28. LeLaurin JH, Lamba AH, Eliazar-Macke ND, et al. Postdischarge Intervention for Stroke Caregivers: Protocol for a Randomized Controlled Trial. *JMIR Res Protoc*. 2020;9(11):e21799. doi:10.2196/21799

29. Das SK, Banerjee TK, Biswas A, et al. A Prospective Community-Based Study of Stroke in Kolkata, India. *Stroke*. 2007;38(3):906-910. doi:10.1161/01.STR.0000258111.00319.58

30. Easton P, Entwistle VA, Williams B. Health in the "hidden population" of people with low literacy. A systematic review of the literature. *BMC Public Health*. 2010;10(1):459. doi:10.1186/1471-2458-10-459

31. Pandian JD, Toor G, Arora R, et al. Complementary and Alternative Medicine Treatments Among Stroke Patients in India. *Topics in Stroke Rehabilitation*. 2012;19(5):384-394. doi:10.1310/tsr1905-384

For peer review only

For peer review only - http://bmjopen.bmj.com/site/about/guidelines.xhtml

Supplementary File 1: WP2 Interview Guide

Q1. Can you tell me about your role?

Q2. Can you tell me about how your service currently manages patients when they have been discharged from hospital?

Q3. What options do patients have if they need support for their stroke after they have been discharged from hospital?

Q4. Are there any services available to support patients or their relatives in relation to their eating and drinking after they have been discharged from hospital?

Q5. Are there any services available to support patients or their relatives in relation to their psychological adjustment to the stroke after they have been discharged from hospital?

Q6. What works well about support that is available for patients in the community? Are there any aspects that do not work as well?

Q7. What role do the patient's family/relatives have in caring for patients when they have been discharged from hospital?

Q8. Do the patient's family/relatives receive any information about how to provide care for their relative who has had a stroke?

Q9. Do the patient's family/relatives receive any information about how to provide care for their relative who has had a stroke?

Q10. What works well about involving the patient's family/relatives once they are in the community?

Q11. Are there any aspects that do not work well?

Q12. Is there anything else that you would like to add?

Thank you for taking the time to take part in this interview.

For peer review only

Study title: **The National Institute for Health Research Global Health Research Group on improving stroke care in India at The University of Central Lancashire - Advancing the INSTRuCT Operations and Network.**

Short title: ***IMPROVIng Stroke care in India - Advancing The INSTRuCT Operations and Network*** **(IMPROVIS-ATION)**

Protocol version: No. 1
Date: 29th June 2020

For peer review only

Protocol version: No. 1
Date: 29th June 2020

For peer review only - http://bmjopen.bmj.com/site/about/guidelines.xhtml

Contents

i. Study team – Roles & Contact details 4
ii. Study summary 5
iii. Abbreviations 7
1. Background 8
2. Aims of the research 11
Figure 1. Study flow chart 12
2.1 Overall participant timeline 13
Figure 2. Study period timeline 13
3. WP1 Protocol: Hydration and swallowing in hospital 14
3.1 WP1 aim and objectives 14
3.2 WP1 study design 14
3.3 WP1 study setting 14
3.4 WP1 participant eligibility 15
3.5 WP1 consent procedure 15
3.6 WP1 data collection 15
3.7 WP1 outputs 156
4. WP2 Protocol: Post-discharge support 186
4.1 WP2 aims and objectives 186
4.2 WP2 study design & methods 187
4.3 WP2 study setting 197
4.4 WP2 participant eligibility 197
4.5 WP2 participant recruitment 17
4.6 WP2 consent procedures 198
4.7 WP2 data collection 18
4.8 WP2 outputs 18
5. WP3 Protocol: Multi-site ethical approval process 219
5.1 WP3 aim and objectives 219
5.2 WP3 study design 219
5.3 WP3 study setting 20
5.4 WP3 participant eligibility 20
5.5 WP3 participant recruitment 20
5.6 WP3 data collection 20
5.7 WP3 consent procedure 20
5.8 WP3 outputs 20

For peer review only - http://bmjopen.bmj.com/site/about/guidelines.xhtml

6. Overall operations across work packages 24
6.1 Analysis 24
6.2 Data management 24
6.3 Monitoring 25
6.4 Ethics and regulatory approval 25
6.5 Adverse events & safeguarding 263
6.6 Dissemination plan 26
6.7 Study management 26
7. References 288
8. Appendices and topic guides 289

For peer review only

i. Study team – Roles & Contact details

Name	Role (*Specialism*)	Institution	Contact
Professor Dame Caroline Leigh Watkins	Director	UCLan	CLWatkins@uclan.ac.uk
Denise Forshaw	Deputy Director (*Operations and Governance, Lancashire CTU*)	UCLan	DForshaw@uclan.ac.uk
Professor Catherine Elizabeth Lightbody	Deputy Director (*Nursing; Stroke*)	UCLan	CELightbody@uclan.ac.uk
Dr Pallab Maulik	Deputy Director (*Psychiatry*)	George Institute For Global Health	PMaulik@georgeinstitute.org.in
Professor Jeyaraj Pandian	Deputy Director (*Neurology*)	Christian Medical College	jeyarajpandian@hotmail.com
Dr Liz Boaden	Collaborator (*Stroke*)	UCLan	EBoaden1@uclan.ac.uk
Professor Dominique Cadilhac	Collaborator (*Nursing; Research*)	Monash University, Australia	dominique.cadilhac@monash.edu
Professor Andy Clegg	Collaborator (*Health services*)	UCLan	AClegg3@uclan.ac.uk
Professor Mark Gabbay	Collaborator (*Health services*)	University of Liverpool	M.B.Gabbay@liverpool.ac.uk
Rachel Georgiou	Collaborator (*Stroke*)	UCLan	RGeorgiou@uclan.ac.uk
Dr Jo Gibson	Collaborator (*Stroke*)	UCLan	JGibson4@uclan.ac.uk
Professor Maree Hackett	Collaborator (*Epidemiology*)	UCLan	maree.hackett@sydney.edu.au
Dr Yogeshwar Kalkonde	Collaborator (*Neurology*)	Society for Education, Action and Research in Community Health, India	yvkalkonde@gmail.com
Professor Caroline Sanders	Collaborator (*Patient and Carer engagement*)	The University of Manchester	Caroline.Sanders@manchester.ac.uk
Professor Padma Srivastava	Collaborator (*Stroke*)	All India Institute of Medical Sciences	vasanthapadma123@gmail.com
Professor PN Sylaja	Collaborator (*Stroke*)	Sree Chitra Tirunal Institute for Medical Sciences and Technology	sylajapn@sctimst.ac.in
Kamran Baqai	Research Associate	UCLan	KBaqai1@uclan.ac.uk
Dr Steph Jones	Programme Manager	UCLan	SJones10@uclan.ac.uk
Dr Gordon Prescott	Lead Statistician	UCLan	GPrescott1@uclan.ac.uk
Jo Weldon	Research Associate	UCLan	jcweldon@uclan.ac.uk

Protocol version: No. 1
Date: 29th June 2020

For peer review only - http://bmjopen.bmj.com/site/about/guidelines.xhtml

ii. **Study summary**

**Title**	**IMPROVI**ng **S**troke care in India **- A**dvancing **T**he **I**NSTRUCT **O**perations and **N**etwork (**IMPROVIS-ATION**)
**Design**	Exploratory studies
**Aim**	Work package 1 (WP1) aim:<br>• To explore and gain understanding of how stroke services are set up and operate in order to identify how to implement a hydration and swallowing care bundle.<br><br>Work package 2 (WP2) aims:<br>• To map the current resources available to manage hydration and swallowing problems and psychosocial support following discharge.<br>• Identify the key components of potential interventions for the management of hydration and swallowing, and psychosocial support post-discharge, and explore potential options for interventions with clinical staff, relatives of stroke patients and stroke patients.<br><br>Work package 3 (WP3) aim:<br>• With key stakeholders, to map out the current ethics approval process, identify challenges, enablers and the feasibility of establishing a stream-lined multi-centre ethics approval process for stroke studies within the Indian Stroke Clinical Trial (INSTRuCT) network.
**Population**	WP1 & 2: Convenience sample of stroke patients and/or their relatives and staff from a range of stroke services.<br><br>WP3: Key stakeholders from INSTRuCT network, Forum for Ethics Review Committees in India (FERCI), and the Indian Council of Medical Research (ICMR)
**Research methods**	Qualitative methods – focus groups and interviews.<br><br>WP1: Focus groups and/or interviews- around 10 members of staff, 10 patients and 10 relatives at 5 sites will be included (n=≤150)<br><br>WP2: Focus groups and/or interviews- around 10 members of staff, 10 patients and 10 relatives at 8 sites will be included. Focus groups will be held on two occasions (n=≤480 overall)<br><br>WP3: Focus groups and/or interviews involving around 10 people will take place at convenient locations with around 60 key stakeholders; identified from across 30 hospital sites and 20-30 key stakeholders from organisations such as FERCI and ICMR (n=≤60).
**Outputs**	WP1:<br>• An understanding of current service provision and processes<br>• An understanding of barriers and facilitators to the implementation of a hydration and swallowing care bundle from a range of perspectives<br><br>WP2:<br>• An understanding of current service provision and resources<br>• An understanding of the needs and preferences of patients and their relatives, and clinical staff in relation to the management of hydration and swallowing and psychosocial support following discharge from hospital<br>• Identification of key components of interventions for supporting patients and their relatives following discharge from hospital

Protocol version: No. 1
Date: 29th June 2020

For peer review only - http://bmjopen.bmj.com/site/about/guidelines.xhtml

	WP3:<br>• An understanding of the current ethics approval processes<br>• Identification of potential challenges, enablers, and feasibility of developing a stream-lined multi-centre ethics approval process for stroke studies within the INSTRuCT network.
**Duration**	1 year (1 August 2020 – 31 July 2021)
**Sponsor**	The University of Central Lancashire
**Funder**	National Institute of Health Research (NIHR)
**Chief Investigator**	Professor Dame Caroline Leigh Watkins
**Collaborating Institutes**	• University of Central Lancashire, UK<br>• University of Manchester, UK<br>• University of Liverpool, UK<br>• Monash University, Australia<br>• Christian Medical College, Ludhiana, India<br>• All India Institute of Medical Sciences, New Delhi, India<br>• Sree Chitra Tirunal Institute of Medical Sciences, Trivandrum, India<br>Research sites<br>• All India Institute of Medical Sciences (AIIMS), Bhopal, India<br>• Baptist Christian Hospital (BCH), Tezpur, India<br>• Indira Gandhi Medical College & Hospital (IGMC), Shimla, India<br>• National Institute of Mental Health and Neurosciences (NIMHANS), Bangalore, India<br>• Zydus Hospital, Ahmedabad, India<br>• Government Hospital Trivandrum, India

For peer review only - http://bmjopen.bmj.com/site/about/guidelines.xhtml

iii. Abbreviations

**CDSCO**	Central Drugs Standard Control Organization
**CI**	Chief Investigator
**FERCI**	Forum for Ethics Research Committees in India
**GCP**	Good Clinical Practice
**HMSC**	Health Ministry Screening Committee
**ICMR**	Indian Council of Medical Research
**IMPROVISE**	IMPROVIng StrokE care in India
**IMPROVIS-ATION**	IMPROVIng Stroke care in India - Advancing The INSTRuCT Operations and Network
**INSTRuCT**	Indian Stroke Clinical Trial Network
**NIHR**	National Institute for Health Research
**NBM**	Nil By Mouth
**NGT**	Nasogastric tube
**PI**	Principal Investigator
**SAE**	Serious Adverse Event
**SLT**	Speech and Language Therapist
**STKG**	Stakeholder Group
**WP**	Work Package

For peer review only

For peer review only - http://bmjopen.bmj.com/site/about/guidelines.xhtml

1. Background

Stroke is a recognized global health challenge. Cardiovascular disease including stroke is now the most common cause of death globally (World Health Organisation, 2014). India is one of the three countries with the greatest number of recorded stroke deaths worldwide (Mackay and Mensah, 2004). People in India typically suffer a first stroke at a much younger age than in the UK (India, age 57 (Pandian and Sudhan, 2013); UK, age 72 (male) and age 78 (female) (The Stroke Association 2018), leading to significant socio-economic burden (Dalal, 2006).

In the last 20 years, the UK, along with other higher income countries, has achieved significant improvements in stroke outcomes, including reduced mortality and morbidity. Much of this improvement has been through the introduction of organised stroke unit care. Stroke unit care has great potential to reduce mortality and morbidity worldwide, as it is the most widely applicable and effective intervention (Stroke Unit Trialist Collaboration, 2013).

One element of stroke unit care that is thought to be effective is an organised specialist multidisciplinary team approach and patient-centered care. It has been demonstrated that better adherence to several processes of care in acute stroke is associated with reduced mortality. A recent audit (Bray et al., 2013) showed that the performance of a swallow assessment, and provision of adequate fluid and nutrition, were all associated with lower 30-day mortality. Adherence to these and other care quality indicators may be amenable to improvement via structured intervention.

One way of delivering structured interventions is via care bundles. Care bundles aim to improve standards of care and patient outcomes by promoting the consistent implementation of a group of effective interventions, which when performed together, may have a better outcome than if performed individually (Cadilhac et al., 2017). They can be used to ensure the delivery of the minimum standard of care. One of the key elements of acute stroke care which are suitable for implementation via care bundles are hydration and swallowing.

In this study we will build on the National Institute for Health Research Global Health Research Group funded project Improving Stroke Care in India (IMPROVISE), in which a Care Bundle was

For peer review only - http://bmjopen.bmj.com/site/about/guidelines.xhtml

implemented to prevent dehydration in hospital, and to improve staff competence to assess dysphagia and manage oral intake. This reduced the need for patients screening positive for dysphagia to stay nil by mouth (NBM) to avoid aspiration pneumonia. These elements rely heavily on having appropriately educated and skilled nurses, working within a multidisciplinary team, to perform them effectively. IMPROVISE has introduced training and care pathways to allow staff to follow the initial dysphagia screen with comprehensive assessment to inform in-hospital management of oral intake. In this study we would like to explore and gain an understanding of how stroke services are set up and operate in order to identify how to implement a hydration and swallowing care bundle through interviews and discussions across 5 hospitals.

During exploration of how best to support patients and families to engage in in-hospital care, the issue of psychosocial support post-discharge has been raised; there is little available. Our co-applicants' ATTEND Trial also found that the trial Rehabilitation Therapists were hampered in their efforts to provide rehabilitation support because patients and families wanted instead to focus on psychosocial issues (The ATTEND Collaborative Group, 2017). Although there is a paucity of data on the long-term consequences of stroke on families in India, anecdotal evidence indicates a significant burden, particularly in rural areas, and in less educated families (Dalal, 2006).

In India, most people with stroke have recovery supported by families and their psychosocial problems could differ significantly from those experienced by families in developed countries. In a study of 152 carers (mean age 40·5), 85% had carer strain, with depression in 43% and anxiety in 47% (Raju et al., 2012). Organised support for patients and families is rare but crucial.

In IMPROVIS-ATION we will explore through interviews and discussion the potential for providing post-discharge support psychosocial support as well as hydration and swallowing. This could improve patient complications and reduce psychological distress for both patients and relatives, reducing morbidity and mortality.

During IMPROVISE and through discussions with our India-based collaborators, it was identified that a streamlined ethical approvals process could help to facilitate stroke research studies. Indian research ethics requires individual approval from each site, with Health Ministry Screening Committee (HMSC) approval finally conferred only after all site approvals are gained. Preliminary discussions with members of Indian Stroke Clinical Trial

For peer review only

Network (INSTRuCT) the Forum for Ethics Research Committees in India (FERCI) and the Indian Council for Medical Research (ICMR) suggest that there is the potential for a multi-centre ethics processes which would facilitate stroke and other multi-site clinical research studies in the future.

For peer review only

Protocol version: No. 1
Date: 29th June 2020

For peer review only - http://bmjopen.bmj.com/site/about/guidelines.xhtml

2. Aims of the research

Three exploratory work package studies (WPs) will establish the foundations to underpin future planned work to improve the delivery of acute and community stroke services that support stroke survivors.

<u>Work package 1 (WP1) aim:</u>

- To explore and gain an understanding of how stroke services are set up and operate in order to identify how to implement a hydration and swallowing care bundle.

<u>Work package 2 (WP2) aims:</u>

- To map the current resources available for hydration and swallowing problems, and psychosocial support following discharge.
- To identify the key components of potential interventions for the management of hydration and swallowing, and psychosocial support, post-discharge; and, explore potential options for interventions with clinical staff, stroke patients, and their relatives.

<u>Work package 3 (WP3) aim:</u>

- With key stakeholders, to map out the current ethics approval process, identify challenges, enablers and the feasibility of establishing a stream-lined multi-centre ethics approval process for stroke studies within the INSTRuCT network.

Figure 1. Study flow chart

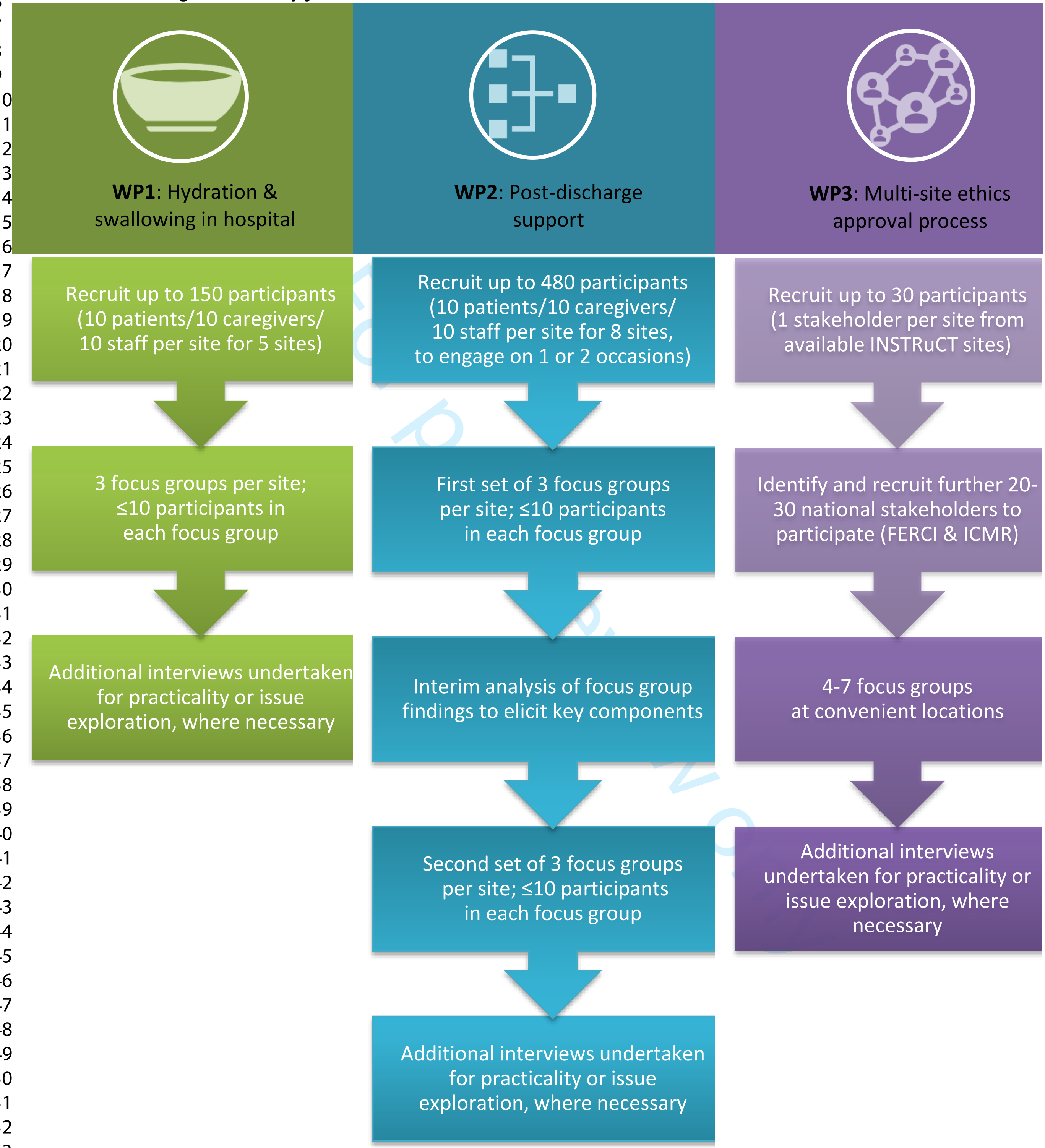

Protocol version: No. 1
Date: 29th June 2020

2.1 Overall participant timeline

Participants will be recruited between 1 November 2020 and 31 March 2021.

Figure 2. Study period timeline

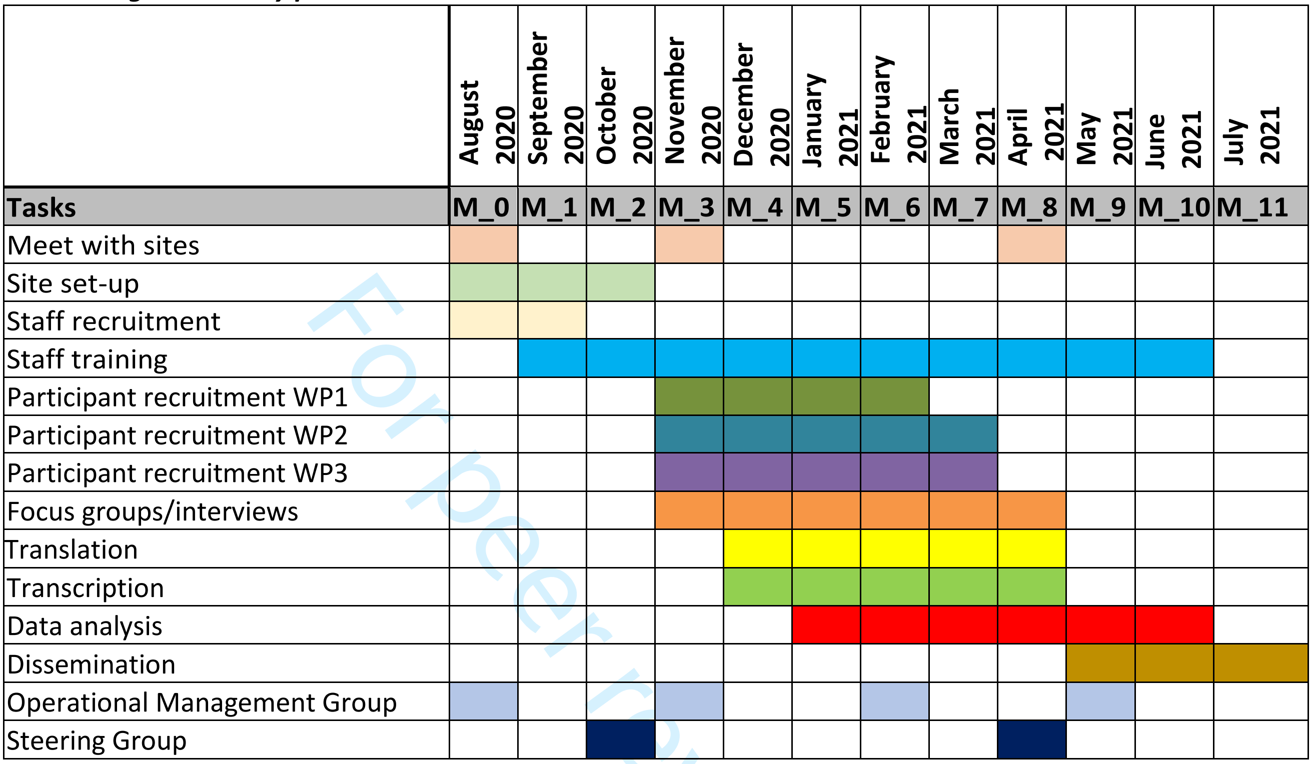

For peer review only

Protocol version: No. 1
Date: 29th June 2020

For peer review only - http://bmjopen.bmj.com/site/about/guidelines.xhtml

3. WP1 Protocol: Hydration and swallowing in hospital

3.1 WP1 aim and objectives

Aim: To explore and gain an understanding through interviews and focus groups of how stroke services are set up and operate, in order to identify how to implement a hydration and swallowing care bundle.

Objectives:

- To explore with stroke patients, families and staff, current screening, assessment and management for hydration and swallowing
- To understand challenges and solutions to the implementation of a hydration and swallowing management care bundle in hospital
- To understand regional differences
- To identify the key components for a potential implementation strategy of the hydration and swallowing management care bundle

3.2 WP1 study design

An exploratory study using qualitative research methods. Guided by a facilitator, focus groups will be conducted in local languages (with translators present to support participant engagement), with a note-taker and a secondary facilitator. Focus groups will explore topics including: experiences of access to and resources available for the assessment and management of hydration and swallowing; what people would want in relation to improving hydration and swallowing assessment and management within their locality and how this could be achieved. Focus groups will be conducted face-to-face or online using Zoom or similar technology. Semi-structured interviews will explore the same topics as in the focus groups; interviews may be undertaken to supplement focus group discussions, to follow up on focus group findings in more detail, and/or to ensure inclusivity where participant attendance at the focus groups is not feasible due to disabilities, clinical commitments and other practicalities. Interviews will also be conducted in local languages (with translators present, if required), and may be conducted either face-to-face or by telephone or using Zoom or similar technology (Topic guides Appendix 12 (staff members); Appendix 13 Patients/relatives).

3.3 WP1 study setting

This study will be conducted at five hospital sites across India:

For peer review only

1. All India Institute of Medical Sciences (AIIMS), Bhopal
2. Baptist Christian Hospital (BCH), Tezpur
3. Indira Gandhi Medical College & Hospital (IGMC), Shimla
4. National Institute of Mental Health and Neurosciences (NIMHANS), Bangalore
5. Zydus Hospital, Ahmedabad

3.4 WP1 participant eligibility

We will identify a convenience sample (using non-restrictive criteria) of patients and/or their relatives, and staff, with experience of stroke from a range of stroke services.

Subjects

We will recruit up to 150 participants Each site will host three focus groups, which include ≤10 participants in each staff, or patient and relative focus group (maximum individual commitment of 2-4 hours).

Patients

- Eligible for inclusion in the study are adult patients (aged ≥18 years) with experience of stroke or TIA.
- Excluded patients are those with no legally acceptable representative to consent

Relatives

- Eligible for inclusion in the study are adult (aged ≥18 years) relatives or caregivers for the person who has had the stroke or TIA.

Staff

Clinical staff (medical, nursing and therapy) will also be invited to participate.

For peer review only

3.5 WP1 consent procedure

The study will follow the consent procedure as listed in ICH GCP section 4.8 Informed Consent of Trial Subjects, Indian Council of Medical Research (ICMR), Ethical Guidelines for Biomedical Research on Human Participants and Indian Good Clinical Practices as laid down by Central Drugs Standard Control Organization (CDSCO).

Each patient/relative (Appendix 1 (WP1) and Appendix 5 (WP2)) and staff member (Appendix 3 (WP1)and Appendix 7 (WP2)) will be given a Participant Information Sheet (PIS) (which describes the study by a site RA or member of the clinical team. Participants agreeing to take part will be asked to provide written informed consent (Patients/relatives: Appendix 2 (WP1; Appendix 6 (WP2); Staff members: (Appendix 4 (WP1) and Appendix 8 (WP2)) before their focus group/interview; and, prior to any data being collected.

A legally acceptable representative may sign the consent form on behalf of an illiterate patient after verbally explaining the study details to them (illiterate patients to provide their thumb impression alongside signature of the legally acceptable representative). If neither a patient nor their legally acceptable representative are literate, an impartial witness (any person completely unrelated to the clinical study) may explain the details of the PIS to the patient and sign the consent form on behalf of the patient (who will provide their thumb impression alongside the signature of the impartial witness).

A copy of the consent form will be given to the participant. The original copy will be kept in the relevant Site File.

3.6 WP1 data collection

Demographic (age; sex; occupation) and stroke detail (type of stroke; severity of stroke [mild/moderate/severe]; experience of swallowing difficulties [current/recent/previous]; psychosocial difficulties (WP2 only); time since stroke; any disability [modified Rankin scale: MRS]) will be collected for all recruited participants from the patient and/or relative (Appendix 9). If this is not possible, this information will be recorded from the patient's medical records by a member of the clinical team.

Details of relationship to patient and age will be collected for recruited relatives in the consent form.

Job role, grade and place of work will be collected for all recruited staff members in the consent form.

3.7 WP1 outputs

- An understanding of current service provision and processes
- An understanding of barriers and facilitators from a range of perspectives

For peer review only - http://bmjopen.bmj.com/site/about/guidelines.xhtml

For peer review only

For peer review only

Protocol version: No. 1
Date: 29th June 2020

For peer review only - http://bmjopen.bmj.com/site/about/guidelines.xhtml

4. WP2 Protocol: Post-discharge support

4.1 WP2 aims and objectives

Aim: Through interviews and focus groups to:

- Map the current resources available for hydration and swallowing problems and psychosocial support following discharge
- Identify the key components of potential interventions for the management of hydration and swallowing, and psychosocial support post-discharge, and explore potential options for interventions with clinical staff, relatives of stroke patients, and stroke patients

Objectives:

- To explore patient pathways post-discharge and understand resources available for care
- To understand patients' and relatives' concerns, contextual challenges, and solutions
- To understand regional differences
- To identify key components of potential interventions

4.2 WP2 study design

An exploratory study using qualitative research methods. Guided by a facilitator, focus groups will be conducted in local languages (with translators present to support participant engagement), with a note-taker and a secondary facilitator. Focus groups will explore topics including: experiences of, access to and resources available for the on-going assessment and management of hydration and swallowing and post-discharge support; what people would want in relation to improving post-discharge support within their locality and how this could be achieved. Focus groups will be conducted face-to-face or online using Zoom or similar technology. Semi-structured interviews will explore the same topics as in the focus groups; interviews may be undertaken to supplement focus group discussions, to follow up on focus group findings in more detail, and/or to ensure inclusivity where participant attendance at the focus groups is not feasible due to disabilities, clinical commitments and other practicalities.

For peer review only - http://bmjopen.bmj.com/site/about/guidelines.xhtml

Interviews will also be conducted in local languages (with translators present, if required), and may be conducted either face-to-face or by telephone or using Zoom or similar technology. (Topic guides Appendix 14 (staff members); Appendix 15 Patients/relatives).

4.3 WP2 study setting

This study will be conducted at eight hospital sites in India, the five listed above and:

1. All India Institute of Medical Sciences (AIMS), New Delhi.
2. Christian Medical College (CMC), Ludhiana, Punjab.
3. Sree Chitra Tirunal Institute of Medical Sciences (SCTIMS), Trivandrum, Kerala.

Subjects

We will recruitment a maximum of 480 participants across the 8 sites. Each site will host a maximum of six focus groups, which include ≤10 participants (staff, or patient and relative focus group).

4.4 WP2 participant eligibility

We will identify a convenience sample (using non-restrictive criteria) of patients and/or their relatives, and staff, with experience of stroke from a range of stroke services.

Patients

- Eligible for inclusion in the study are adult patients (aged ≥18 years) discharged from hospital with experience of stroke.
- Excluded patients are those with no legally acceptable representative to consent

Relatives

- Eligible for inclusion in the study are adult (Aged ≥18 years) relatives or caregivers for the person who has had the stroke following discharge home from hospital.

Staff

Clinical staff (medical, nursing and therapy) will also be invited to participate.

Protocol version: No. 1
Date: 29th June 2020

For peer review only - http://bmjopen.bmj.com/site/about/guidelines.xhtml

4.5 WP2 participant recruitment

At each hospital site, the site Research Associates (RAs) and/or clinical team will invite patients, and any relatives required to support them, to take part in a focus group or interview; where possible these participants will reflect a range of characteristics, as applicable (age/sex/severity of stroke/experience of swallowing and/or psychosocial difficulties).

The site RAs will also invite a purposive sample of staff participants to represent a range of disciplines and grades of staff involved in the management of hydration and swallowing or post-discharge support.

Participants may choose to contribute to either one or two focus groups and/or interviews (maximum individual commitment of 2-4 hours).

4.6 WP2 consent procedure

As described in WP1.

4.7 WP2 data collection

As described in WP1.

4.8 WP2 outputs

- An understanding of current service provision and resources
- An understanding of the needs and preferences of patients and their relatives, and clinical staff, in relation to the management of hydration and swallowing, and psychosocial support following discharge from hospital
- Identification of the key components of interventions for supporting patients and their relatives following discharge from hospital

For peer review only - http://bmjopen.bmj.com/site/about/guidelines.xhtml

5. WP3 Protocol: Multi-site ethical approval process

5.1 WP3 aim and objectives

Aim: With key stakeholders, to map out the current ethics approval process, identify challenges, enablers and the feasibility of establishing a stream-lined multi-centre ethics approval process for stroke studies within the Indian Stroke Clinical Trial (INSTRuCT) network.

Objectives:

- To understand challenges
- To identify enhanced processes for ethical approval

5.2 WP3 study design

Design: an exploratory study using qualitative research methods.

Focus groups will be guided by a facilitator, conducted in local languages (with translators present to support participant engagement), with a note-taker and a secondary facilitator (if required). Focus groups will be conducted face-to-face at convenient locations or online using Zoom or similar technology. Focus group discussion topics will include: How the current ethics approval processes currently operates; what are the current challenges; are there any changes that the group like to see made to the ethics approval process and how might these be implemented.

Covering the same topics, semi-structured interviews may be undertaken to inform focus group discussions, to follow up on focus group findings in more detail, and/or to ensure inclusivity where participant attendance at focus groups is not feasible due restrictions on movement, other practicalities and clinical commitments. Interviews will also be conducted in local languages (with translators present to support participant engagement) and may be conducted either face-to-face or by telephone or video-conferencing technology (Stakeholder topic guide Appendix 16).

5.3 WP3 study setting

The INSTRuCT network relevant organisations, members of the Forum for Ethics Review Committees in India (FERCI) and the Indian Council of Medical Research (ICMR).

For peer review only

5.4 WP3 participant eligibility

Key stakeholders will be invited to participate in focus groups and interviews as follows:

- Employed by an appropriate INSTRuCT site, within either stroke or ethics unit
- From relevant organisations, such as the Forum for Ethics Review Committees in India (FERCI) and the Indian Council of Medical Research (ICMR)
- Actively involved in developing multi-centre research ethics processes though IMPROVIS-ATION

5.5 WP3 participant recruitment

We will recruitment a maximum of 60 participants (30 INSTRuCT network site stakeholders (one per site) and up to 30 FERCI and/or ICMR stakeholders). The programme manager will invite site stakeholders to take part, and organisational stakeholders (FERCI/ICMR) will self-nominate themselves to participate after initial approach through the INSTRuCT network.

Between 4-7 focus groups will be conducted at convenient locations, which include ≤10 participants in each focus group (any combination of available stakeholders; maximum individual commitment of 2-4 hours).

The findings from the focus groups and interviews will be synthesised and presented at a final discussion group to identify the key components of any changes and to agree next steps.

5.6 WP3 data collection

Job role, place of work and ethics committee experience information will be collected for all recruited key stakeholders in the consent form.

5.7 WP3 consent procedure

Each identified key stakeholder will be given a PIS describing the study (Appendix 10) by a member of the research team, issued via e-mail. Any key stakeholder agreeing to take part will be asked to provide written informed consent (Appendix 11) prior to their participation in a focus group or interview.

For peer review only

For peer review only - http://bmjopen.bmj.com/site/about/guidelines.xhtml

5.8 WP3 outputs

- An understanding of the current ethics approval processes
- Identification of potential challenges, enablers, and feasibility of developing a stream-lined multi-centre ethics approval process for stroke studies within the INSTRuCT network.

For peer review only

Protocol version: No. 1
Date: 29th June 2020

For peer review only - http://bmjopen.bmj.com/site/about/guidelines.xhtml

6. Overall operations across work packages

6.1 Analysis

Demographic data will be analysed descriptively. Focus groups/Interviews will be transcribed, and then analysed utilising NVivo (a qualitative data analysis computer software package) to support the analysis process. Framework will be used, this permits identification and cross-classification of variables through: identification of key concepts, mapping their range and diversity, interpreting patterns of association and investigating possible reasons. This would give a local service provider perspective on barriers/facilitators to implementing the potential pathways/reforms in the future, ensuring services fit needs. Discussion notes will also be collated and summarised.

6.2 Data management

Data will be treated and stored according to the General Data Protection Regulation (Information Commissioners Office 2018), the Data Protection Act (2018), Caldicott Principles (Caldicott 2013) and the National Institute for Health Research (NIHR) Good Clinical Practice (GCP) Standards.

Written consent forms will be stored in each hospital's Site File (WPs 1 and 2) and at CMC, the Co-ordinating Centre (WP3). All other data will be stored electronically in password-protected files. Members of the research team will have access to project files and data as appropriate to their role.

Participants will be assigned a participant number, this will be used to anonymise their data. Their anonymised data will be typed up into electronic format and stored in a secure OneDrive folder. Audio files from digital voice recorders will be translated, translations checked and transcribed. Once the transcription has been checked the audio file will be deleted. Anonymised transcriptions will be stored on the secure University of Central Lancashire network for five years following completion of the study in-line with the UCLan Data Protection Code of Practice.

Any individuals' names mentioned in the focus group/interview discussions will be removed from transcription. It will not be possible to recognise individuals from information in the completed data analysis or write up of final reports and publications, and any direct quotes used will remain anonymous.

For peer review only - http://bmjopen.bmj.com/site/about/guidelines.xhtml

6.3 Monitoring

The Project Co-ordinator from CMC and Research Programme Manager at the University of Central Lancashire will monitor training, recruitment and consent procedures.

Monitoring will facilitate confirmation of site adherence to this protocol and Good Clinical Practice (GCP) Guidelines, relevant local and regional ethical requirements, data accuracy and quality. The study may also be audited by external government or regulatory authorities.

In WPs 1 and 2, each site is responsible for maintaining a Site File containing a delegation log, a training log, and validity of consent log. In WP3, CMC will retain a Site File containing: a delegation log and validity of consent log.

Access to files will be made available by all participating sites for monitoring and audit purposes. On-site monitoring will be undertaken by the University of Central Lancashire in the form of (i) scheduled site visits during the study; or, (ii) triggered site visits in response to concerns relating to any study research processes.

6.4 Ethics and regulatory approval

The work packages, developed under this protocol, will be designed and implemented to comply with Ethical Principles for Medical Research Involving Human Patients (World Medical Association Declaration of Helsinki), Ethical Guidelines for Biomedical Research on Human Participants by Indian Council of Medical Research (ICMR), UK

Ethical and Research Governance approval will be obtained from individual sites' ethics committees and The University of Central Lancashire. Health Ministry Screening Committee (HMSC) approval will be sought from ICMR. If appropriate, the study will be registered on www.ctri.nic.in (Clinical Trials Registry-India (CTRI)) before study (patient-related) activities commence.

The CI is responsible for communicating any protocol amendments to the site PI. The site PI is responsible for ensuring that amendments are implemented, and that all documents replaced by 'new' versions are destroyed immediately. Site staff need to be informed of, and start using, new documentation as soon as local approvals are in place.

The site PI is responsible for reporting any protocol violations and routine reporting

For peer review only - http://bmjopen.bmj.com/site/about/guidelines.xhtml

outcomes, reporting to the CI and to their own ethical and governing bodies according to the requirements of the study protocol and written approval of the Ethics Committee.

<u>Consent withdrawal</u>

Participants may withdraw from the study without providing a reason. In these circumstances, the participant will be asked if we can retain and use their data. The PIS emphasises participation is voluntary and that people have the right to withdraw up until they take part in a focus group/discussion, or to the point where their data is anonymised after taking part in an interview and the data becomes fully anonymised once the data have been combined for analysis

6.5 Adverse events & safeguarding

Any adverse events directly resulting from the study will be reported to the site PI (who will inform the Programme Manager in the UK), in accordance with ICH Good Clinical Practices.

Any identified safeguarding concerns will be managed in accordance with the policies and procedures at each individual site.

6.6 Dissemination plan

A comprehensive dissemination plan will be developed with stakeholders, including outputs in clinical and academic peer-reviewed journals, conferences and social media outlets.

Study-generated data will be made available to other researchers for secondary research purposes, as agreed by the partnership and in accordance with open access requirements. This process will be managed and assured by the University of Central Lancashire, in accordance with institutional policy and procedures.

6.7 Study management

The study will be managed in accordance with the governance structure and processes in place for the NIHR Global Health Research Group (GHRG).

For peer review only - http://bmjopen.bmj.com/site/about/guidelines.xhtml

For peer review only

Professor Dame Caroline Watkins is the Director of the NIHR GHRG, with support from Deputy Directors: Professor Liz Lightbody, Deputy Director (Nursing Stroke), UCLAN; Professor Jeyaraj Pandian, Deputy Director (Neurology), Christian Medical College; Dr Pallab Maulik, Deputy Director (Psychiatry), The George Institute for Global Health. Members of the group have worked collaboratively to deliver a number of innovative large-scale multi-disciplinary studies in stroke including in the UK and India. Further details of the group are available at uclanglobalhealth.co.uk

The progress of the project will be overseen every three months by the Operational Management Group (OMG), which will ensure effective operational management of the project with reference to agreed work plans, milestones and key deliverables. The OMG consists of the Director, Deputy Directors, Work Package and site leads, and the Programme Manager.

The overall delivery of the programme will be overseen by the Steering Group, which provides oversight, advice and support to the project team. The Steering Group will make recommendations relating to project delivery to the funder and project team, undertake periodic reviews, and is responsible for the content of formal reports to the programme funder (NIHR). The group consists of a Chair, Directors, work package leads, representative of the funder, and other experts independent of the programme team. The Steering Group will meet regularly, approximately every 3-6 months depending on the level of activity in the programme.

For peer review only

For peer review only - http://bmjopen.bmj.com/site/about/guidelines.xhtml

7. References

Bray B, Ayis S, Campbell J, Hoffman A, Roughton M, Tyrrell PJ, Wolfe CDA, Rudd AG Associations between the organisation of stroke services, process of care, and mortality in England: prospective cohort study. BMJ. 2013, 346

Cadilhac DA, Andrew NE, Lannin NA, Middleton S, Levi CR, Dewey HM, Grabsch B, Faux S, Hill K, Grimley R, Wong A, Sabet A, Butler E, Bladin CF, Bates TR, Groot P, Castley H, Donnan GA, Anderson CS and Australian Stroke Clinical Registry C. Quality of Acute Care and Long-Term Quality of Life and Survival: The Australian Stroke Clinical Registry. Stroke. 2017;48:1026-1032.

Dalal PM. Burden of stroke: Indian perspective. International Journal of Stroke. 2006; 1:164-6

MacKay J, Mensah G. Atlas of Heart Disease and Stroke. World Health Organisation. 2004.

Pandian JD, Sudhan P. Stroke epidemiology and stroke care services in India. Journal of Stroke. 2013;15(3):128-134.

Raju RS, Kaur P and Pandian JD. Psychosocial problems, quality of life, and caregiver burden among stroke caregivers in India. International Journal of Stroke. 2012;7: 100-101. doi:10.1111/j.1747-4949.

Stroke Unit Trialists' Collaboration. Organised inpatient (stroke unit) care for stroke. Cochrane Database of Systematic Reviews. 2013, Issue 9. Art. No.: CD000197

The ATTEND Collaborative Group. Family-led rehabilitation after stroke in India (ATTEND): A randomised controlled trial. The Lancet, 2017; 390:10094. doi.org/10.1016/S0140-6736(17)31447-2.

The Stroke Association. State of the Nation. Stroke statistics. 2016

World Health Organisation. Global status report on non-communicable diseases. 2014. WHO Press, Switzerland.

Protocol version: No. 1
Date: 29th June 2020

For peer review only - http://bmjopen.bmj.com/site/about/guidelines.xhtml

COREQ (COnsolidated criteria for REporting Qualitative research) Checklist

A checklist of items that should be included in reports of qualitative research. You must report the page number in your manuscript where you consider each of the items listed in this checklist. If you have not included this information, either revise your manuscript accordingly before submitting or note N/A.

Topic	Item No.	Guide Questions/Description	Reported on Page No.
**Domain 1: Research team and reflexivity**			
Personal characteristics			
Interviewer/facilitator	1	Which author/s conducted the interview or focus group?	
Credentials	2	What were the researcher's credentials? E.g. PhD, MD	
Occupation	3	What was their occupation at the time of the study?	
Gender	4	Was the researcher male or female?	
Experience and training	5	What experience or training did the researcher have?	
Relationship with participants			
Relationship established	6	Was a relationship established prior to study commencement?	
Participant knowledge of the interviewer	7	What did the participants know about the researcher? e.g. personal goals, reasons for doing the research	
Interviewer characteristics	8	What characteristics were reported about the inter viewer/facilitator? e.g. Bias, assumptions, reasons and interests in the research topic	
**Domain 2: Study design**			
Theoretical framework			
Methodological orientation and Theory	9	What methodological orientation was stated to underpin the study? e.g. grounded theory, discourse analysis, ethnography, phenomenology, content analysis	
Participant selection			
Sampling	10	How were participants selected? e.g. purposive, convenience, consecutive, snowball	
Method of approach	11	How were participants approached? e.g. face-to-face, telephone, mail, email	
Sample size	12	How many participants were in the study?	
Non-participation	13	How many people refused to participate or dropped out? Reasons?	
Setting			
Setting of data collection	14	Where was the data collected? e.g. home, clinic, workplace	
Presence of non-participants	15	Was anyone else present besides the participants and researchers?	
Description of sample	16	What are the important characteristics of the sample? e.g. demographic data, date	
Data collection			
Interview guide	17	Were questions, prompts, guides provided by the authors? Was it pilot tested?	
Repeat interviews	18	Were repeat inter views carried out? If yes, how many?	
Audio/visual recording	19	Did the research use audio or visual recording to collect the data?	
Field notes	20	Were field notes made during and/or after the inter view or focus group?	
Duration	21	What was the duration of the inter views or focus group?	
Data saturation	22	Was data saturation discussed?	
Transcripts returned	23	Were transcripts returned to participants for comment and/or	

For peer review only

Topic	Item No.	Guide Questions/Description	Reported on Page No.
		correction?	
**Domain 3: analysis and findings**			
Data analysis			
Number of data coders	24	How many data coders coded the data?	
Description of the coding tree	25	Did authors provide a description of the coding tree?	
Derivation of themes	26	Were themes identified in advance or derived from the data?	
Software	27	What software, if applicable, was used to manage the data?	
Participant checking	28	Did participants provide feedback on the findings?	
Reporting			
Quotations presented	29	Were participant quotations presented to illustrate the themes/findings? Was each quotation identified? e.g. participant number	
Data and findings consistent	30	Was there consistency between the data presented and the findings?	
Clarity of major themes	31	Were major themes clearly presented in the findings?	
Clarity of minor themes	32	Is there a description of diverse cases or discussion of minor themes?	

Developed from: Tong A, Sainsbury P, Craig J. Consolidated criteria for reporting qualitative research (COREQ): a 32-item checklist for interviews and focus groups. *International Journal for Quality in Health Care*. 2007. Volume 19, Number 6: pp. 349 – 357

Once you have completed this checklist, please save a copy and upload it as part of your submission. DO NOT include this checklist as part of the main manuscript document. It must be uploaded as a separate file.

For peer review only
